# The effectiveness of abstinence‐based and harm reduction‐based interventions in reducing problematic substance use in adults who are experiencing homelessness in high income countries: A systematic review and meta‐analysis: A systematic review

**DOI:** 10.1002/cl2.1396

**Published:** 2024-04-21

**Authors:** Chris O'Leary, Rob Ralphs, Jennifer Stevenson, Andrew Smith, Jordan Harrison, Zsolt Kiss, Harry Armitage

**Affiliations:** ^1^ Manchester Metropolitan University Manchester UK; ^2^ Independent Consultant Cambridge UK; ^3^ ZK Analytics Oxford UK

## Abstract

**Background:**

Homelessness is a traumatic experience, and can have a devastating effect on those experiencing it. People who are homeless often face significant barriers when accessing public services, and have often experienced adverse childhood events, extreme social disadvantage, physical, emotional and sexual abuse, neglect, low self‐esteem, poor physical and mental health, and much lower life expectancy compared to the general population. Rates of problematic substance use are disproportionately high, with many using drugs and alcohol to deal with the stress of living on the street, to keep warm, or to block out memories of previous abuse or trauma. Substance dependency can also create barriers to successful transition to stable housing.

**Objectives:**

To understand the effectiveness of different substance use interventions for adults experiencing homelessness.

**Search Methods:**

The primary source of studies for was the 4th edition of the Homelessness Effectiveness Studies Evidence and Gaps Maps (EGM). Searches for the EGM were completed in September 2021. Other potential studies were identified through a call for grey evidence, hand‐searching key journals, and unpacking relevant systematic reviews.

**Selection Criteria:**

Eligible studies were impact evaluations that involved some comparison group. We included studies that tested the effectiveness of substance use interventions, and measured substance use outcomes, for adults experiencing homelessness in high income countries.

**Data Collection and Analysis:**

Descriptive characteristics and statistical information in included studies were coded and checked by at least two members of the review team. Studies selected for the review were assessed for confidence in the findings. Standardised effect sizes were calculated and, if a study did not provide sufficient raw data for the calculation of an effect size, author(s) were contacted to obtain these data. We used random‐effects meta‐analysis and robust‐variance estimation procedures to synthesise effect sizes. If a study included multiple effects, we carried out a critical assessment to determine (even if only theoretically) whether the effects are likely to be dependent. Where dependent effects were identified, we used robust variance estimation to determine whether we can account for these. Where effect sizes were converted from a binary to continuous measure (or vice versa), we undertook a sensitivity analysis by running an additional analysis with these studies omitted. We also assessed the sensitivity of results to inclusion of non‐randomised studies and studies classified as low confidence in findings. All included an assessment of statistical heterogeneity. Finally, we undertook analysis to assess whether publication bias was likely to be a factor in our findings. For those studies that we were unable to include in meta‐analysis, we have provided a narrative synthesis of the study and its findings.

**Main Results:**

We included 48 individual papers covering 34 unique studies. The studies covered 15, 255 participants, with all but one of the studies being from the United States and Canada. Most papers were rated as low confidence (*n* = 25, or 52%). By far the most common reason for studies being rated as low confidence was high rates of attrition and/or differential attrition of study participants, that fell below the What Works Clearinghouse liberal attrition standard. Eleven of the included studies were rated as medium confidence and 12 studies as high confidence. The interventions included in our analysis were more effective in reducing substance use than treatment as usual, with an overall effect size of –0.11 SD (95% confidence interval [CI], −0.27, 0.05). There was substantial heterogeneity across studies, and the results were sensitive to the removal of low confidence studies (−0.21 SD, 95% CI [−0.59, 0.17] − 6 studies, 17 effect sizes), the removal of quasi‐experimental studies (−0.14 SD, 95% CI [−0.30, 0.02] − 14 studies, 41 effect sizes) and the removal of studies where an effect size had been converted from a binary to a continuous outcome (−0.08 SD, 95% CI [−0.31, 0.15] − 10 studies, 31 effect sizes). This suggests that the findings are sensitive to the inclusion of lower quality studies, although unusually the average effect increases when we removed low confidence studies. The average effect for abstinence‐based interventions compared to treatment‐as‐usual (TAU) service provision was –0.28 SD (95% CI, −0.65, 0.09) (6 studies, 15 effect sizes), and for harm reduction interventions compared to a TAU service provision is close to 0 at 0.03 SD (95% CI, −0.08, 0.14) (9 studies, 30 effect sizes). The confidence intervals for both estimates are wide and crossing zero. For both, the comparison groups are primarily abstinence‐based, with the exception of two studies where the comparison group condition was unclear. We found that both Assertative Community Treatment and Intensive Case Management were no better than treatment as usual, with average effect on substance use of 0.03 SD, 95% CI [−0.07, 0.13] and –0.47 SD, 95% CI [−0.72, −0.21] 0.05 SD, 95% CI [−0.28, 0.39] respectively. These findings are consistent with wider research, and it is important to note that we only examined the effect on substance use outcomes (these interventions can be effective in terms of other outcomes). We found that CM interventions can be effective in reducing substance use compared to treatment as usual, with an average effect of –0.47 SD, 95% CI (−0.72, −0.21). All of these results need to be considered in light of the quality of the underlying evidence. There were six further interventions where we undertook narrative synthesis. These syntheses suggest that Group Work, Harm Reduction Psychotherapy, and Therapeutic Communities are effective in reducing substance use, with mixed results found for Motivational Interviewing and Talking Therapies (including Cognitive Behavioural Therapy). The narrative synthesis suggested that Residential Rehabilitation was no better than treatment as usual in terms of reducing substance use for our population of interest.

**Authors' Conclusions:**

Although our analysis of harm reduction versus treatment as usual, abstinence versus treatment as usual, and harm reduction versus abstinence suggests that these different approaches make little real difference to the outcomes achieved in comparison to treatment as usual. The findings suggest that some individual interventions are more effective than others. The overall low quality of the primary studies suggests that further primary impact research could be beneficial.

## 1 PLAIN LANGUAGE SUMMARY

Some services work to reduce problematic substance use by adults experiencing homelessness.

### 1.1 The review in brief

People experiencing homelessness sometimes have problems with substance use. This review looked at different services that try to help reduce substance use, to see if they worked. It found that giving people vouchers if they did not use substances worked better than conventional services (which might include no provision or alternative services), and that some evidence that other services might work. Assertative Community Treatment (ACT) and Intensive Case Management (ICM) did not make any difference.

### 1.2 What is this review about?

Substance use is higher among adults experiencing homelessness. Substance use can have terrible consequences, for example, drug overdose is a major cause of death for people experiencing homelessness. Many services try to reduce or stop substance, and it is important to know whether these do work. This review has looked at the available evidence to see whether a service is harm reduction based or abstinence based makes a difference to whether it works, whether these services work overall, and which individual services work.

### 1.3 What is the aim of this review?

This Campbell systematic review examines the effects of different interventions on substance use in adults experiencing homelessness. This review summarises 48 papers covering 9 different services.

### 1.4 What are the main findings of this review?

Most of the papers come from the United States and Canada, and there were no papers from the UK. Each paper compared a new substance use service (the intervention being evaluated) to conventional services, or doing nothing to help. Some of the studies used high quality methods, but most did not.

Overall, we found that the new substance use services that were tested in the 48 papers covered by our review worked better than conventional services.

We looked at whether harm reduction‐based services work better than conventional services, whether abstinence‐based services work better than conventional services, and whether harm reduction‐based services work better than abstinence‐based. This analysis suggests that these different approaches make little real difference to the outcomes achieved. Contingency Management (CM), where vouchers are given to someone to stop using substances, works much better than conventional services. Group Work, Harm Reduction Psychotherapy, and Therapeutic Communities might work reducing substance use, with mixed results found for Motivational Interviewing and Talking Therapies (including Cognitive Behavioural Therapy). The evidence suggested that Residential Rehabilitation, ACT, and ICM did not make a difference compared to conventional services.

Only three papers looked at women experiencing homelessness, and overall three quarters of those involved in the studies were men. This is partly because men are more likely to use substances, and are more likely to experience street homelessness. But the lack of women in these studies is a significant gap.

### 1.5 What do the findings of this review mean?

Most of the papers included are from the US and Canada, and there are big differences between homelessness there and in the UK or Europe. We need more evidence from the UK about what types of substance use services work for people experiencing homelessness. We also need more evidence about women's homelessness, and which services work best for women experiencing homelessness to reduce their substance use.

### 1.6 How up‐to‐date is this review?

Papers published before September 2021 are included in this review.

## 2 BACKGROUND

### 2.1 The problem, condition or issue

Homelessness is a significant social problem and public health concern (MacKnee, 2002). Rising levels of homelessness are reported in many high‐income countries. Homelessness rates have grown significantly across the European Union in the last decade, though the level of growth slowed during the COVID‐19 pandemic (Feantsa, 2022). In England, all forms of homelessness rose between 2008 and 2017 (O'Leary, 2020), and it is estimated that in January 2020, some 280,000 people were homeless in England (Shelter, 2021). Recent published data based on snapshot counts suggests that the number of people who are sleeping rough in England fell each year between 2017 and 2020. It is likely that the count for 2020 will have been affected by government responses to COVID‐19 (MHCLG, 2021). The most recent State of Homelessness in America report stated that in January 2020 over 580,000 were experiencing homelessness in the United States, and that rates of homelessness had grown by 2% over the previous year (National Alliance to End Homelessness, 2022). In Canada, around 35,000 people are homeless each night, with between 250,000 and 300,000 experiencing homelessness a year (Wong, 2020). In Japan, official statistics suggest that the number of people experiencing street homelessness has been falling over the past two decades, and now stands at under 5000. However, it is likely that this underestimates the level of homelessness, and the actual number of people without homes is not known (Fujii, 2022). Despite these country level estimates, it is difficult to get an overall picture of the scale of homelessness because of differences in definitions and measures (OECD, 2020).

Homelessness is a complex and multifaceted social issue. There are significant differences in how homelessness is understood and experienced, and how these differences are conceptualised and described, both between developed countries and more widely. While there is often a focus on the most visible forms of homelessness – people experiencing street homelessness or sleeping rough – homelessness is much wider and more complex, and there are many different forms of homelessness. Globally, there are ongoing policy and practice debate around the causes of homelessness. Glen Bramley and Suzanne Fitzpatrick state that there is significant debate between a focus on individual‐level risks or causes, and structural or systemic causes (such as labour market conditions, housing supply, and poverty). These foci vary between countries and over time, though increasingly it is recognised that both might have explanatory power (Bramley, 2018). There are also policy debates around the types of interventions that might address homelessness, and whether these should be focused on structural interventions such as increasing housing supply or reducing poverty, or preventing/addressing homelessness at the level of the individual.

Homelessness is a traumatic experience and can have a devastating effect (O'Leary, 2022). Several studies, some of which are cited below, have highlighted that more visible and extreme forms of homelessness are often associated with adverse childhood events (Koh & Montgomery, 2021), extreme social disadvantage (Mabhala et al., 2017), physical, emotional and sexual abuse (Green et al., 2012; Henny et al., 2007), neglect (Mar et al., 2014), low self‐esteem, poor physical and mental health (Vallesi et al., 2021), and much lower life expectancy compared to the general population (ONS, 2020). People experiencing homelessness often face severe and multiple disadvantages (Bramley, 2020) and need significant levels of support (Dobson, 2019). There is a growing recognition that homelessness, drug and alcohol misuse, poor mental health, and offending behaviours are experienced by the same individuals (Bramley, 2020). Evidence increasingly shows that longer periods of homelessness are associated with greater severity of these issues (Mayock, 2011).

Adults experiencing homelessness often face significant barriers accessing services, and can fall through the cracks between different services they need to access (Dobson, 2019). Those experiencing the more visible and extreme forms of homelessness have repeated contact with a range of publicly funded services, particularly health (Aldridge, 2018), criminal justice (Bramley, 2020), and local government (Dobson, 2019). For example, this population is five times more likely to attend accident and emergency, and three times more likely to be admitted to hospital, than their housed peers (Cornes, 2018). The existing evidence of effectiveness of interventions for this population is mixed (Bramley, 2020; Luchenski, 2018). Most of the extant evidence that examines the effectiveness of interventions around homelessness included in the Homelessness Evidence and Gap Map (Jain et al., 2023) is focused on individual‐level interventions. They are typically aimed at addressing the harms caused by homelessness or reducing homelessness, rather than prevention.

### 2.2 The relationship between homelessness and substance use

Problematic substance use is disproportionately high among people experiencing or at risk of homelessness (Aldridge, 2018; Magwood, 2020). Many people experiencing both homelessness and problematic substance use are dependent on the substances they use (Chen, 2006; Martijn, 2006; Thompson, 2009). It can be the case that substance use is aimed at alleviating the stress of life on the streets (Klee, 1998a, 1998b; Thompson, 2005), to keep warm (Ayerst, 1999), self‐medicating because of physical and mental health problems (Fountain, 2002; Homeless Link, 2014; Klee, 1998a, 1998b), or to block out memories of previous trauma or abuse (Carver, 2020). Substance dependency presents one of the key challenges faced by those who want to transition from homelessness into stable housing (Tsemberis, 2011).

People experiencing homelessness who use drugs are particularly vulnerable to new synthetic drugs. For example, synthetic cannabinoids, commonly known as ‘Spice’ or ‘K2’ has become synonymous with homeless populations in many countries and was attributed to over 60 drug‐related deaths, many of whom were homeless, in New Zealand in 2018 (New Zealand Drug Foundation, 2018). In the United States, people experiencing homelessness have higher mortality rates by (synthetic) opioid overdose than national averages (National Health Care for the Homeless Council, 2017).

Drug overdose is a major cause of death among people experiencing homelessness (Bauer, 2016). Mortality among people experiencing homelessness who use drugs is high and has been increasing in the past decade. In England and Wales, drug‐related deaths of homeless people increased by 52% between 2012 and 2017 (ACMD, 2019). Recent Office for National Statistics (ONS, 2020) data reported that 37% of the 778 deaths of people experiencing homelessness were related to drug poisoning in 2019. In comparison, drug poisoning accounted for 0.8% of all deaths in the general population in 2019 (ONS, 2020). According to The Dying Homeless Project, the offer of hotel accommodation during the pandemic was successful at preventing deaths from COVID‐19, but deaths still rose by 37%. They recorded 976 deaths across England, Scotland, Wales, and Northern Ireland in 2020 – a 37% increase in the numbers reported in the 2019 study. Over a third (36%) were related to drug and alcohol use; compared to less than 3% directly from COVID‐19 (Museum of Homelessness, 2021).

### 2.3 The intervention

There are a range of interventions that aim to stop, reduce or prevent problematic substance use, both generally and specifically for people experiencing homelessness. These interventions originate in different areas of social policy, including healthcare, criminal justice, education, and housing. We have set out the key interventions to be covered by this review in a typology in Table 1, and provide a description of each intervention in the appendix.

**Table 1:** Intervention typology.

	Abstinence‐based (low tolerance)		Harm reduction based (high tolerance)
Psychosocial interventions	Therapeutic communities	Motivational interviewing	Harm reduction pyschotherapy
	Self‐help (12‐step, AA, NA)	Talking therapies	Education regarding safer injecting techniques & not sharing
	Residential rehabilitation	Assertive outreach	Group work (including RAMP)
	Abstinence based day programmes	Motivational Enhancement Therapy (MET)	Harm reduction based day programmes
	Trauma therapies (including EDMR)	Assertative Community Treatment	
		Intensive Case Management	
		Behavioural Couples Therapy	
		Self‐help (Smart Recovery)	
Treatment through medication	Detoxification	Agonist pharmacotherapy/blockers	Opioid Substitution Treatment (OST)/Maintenance therapy
			NX provision
			Naloxone
			Rapid prescribing
			Testing for BBVs
			Needle exchanges
Non‐medication intervention	Contingency Management (testing, treatment engagement)		Heroin Assisted Therapy (HAT)
	Recovery Housing		Safe Injecting space/DCRs

### 2.4 Treatment approaches: Harm reduction versus abstinence‐based recovery models

An enduring debate is whether interventions that adopt harm reduction or abstinence‐based approaches are more effective. Abstinence‐based interventions start with an assumption that the only way to avoid problematic substance use is to avoid drugs and alcohol completely, and thus abstaining is a requirement of continued participation in the service or intervention. Harm reduction approaches do not impose an abstinence requirement, although the individual may choose to be abstinent. These interventions seek to reduce the risks or harms associated with problematic substance use. The debate around the comparative effectiveness of these approaches is sometimes framed as an either/or dichotomy (Dennis, 2020); other times it is framed as a need to integrate both in a continuum of approaches, ranging from high tolerance harm reduction to low or zero tolerance abstinence‐based approaches.

There is a more substantive empirical literature about the effectiveness of abstinence‐based approaches than exists around harm reduction. This is because most of the effectiveness literature is from North America (and particularly the United States), where abstinence has historically played a more central role in substance misuse treatment compared to Europe, the UK and elsewhere (Bujarski, 2013). It is also reflective of abstinence being a core criterion of recovery (Von Greiff, 2020), particularly for medical‐centred interventions (Demartini, 2014).

Abstinence can be differentiated as either a goal or anticipated outcome of a substance use intervention, or as a conditional requirement for access to non‐substance misuse treatment services such as housing and mental health services. This systematic review is focused on the latter. It is also important to differentiate between abstinence that is required of a person experiencing homelessness, and abstinence as a goal that an individual sets themselves.

### 2.5 How the intervention might work

Abstinence‐based and harm reduction‐based interventions draw on different causal assumptions about how individuals might address or manage their substance use, even where they might have similar goals or outcomes in relation to abstaining from substance use. Harm reduction approaches are more developed in the literature in terms of the underlying causal explanations. In a paper about the ‘active ingredients’ of effective substance misuse treatments, Rudolf Moos (Moos, 2007) identifies four potential theories of change underpinning different treatments, concluding that little is known about how these different social processes generate reductions in substance misuse. Drawing on this limited theoretical work, in the protocol for this study (O'Leary, 2022) we identified two possible ‘theories of change’ at play. These two theories were social control (which underpins many abstinence‐based interventions) and self‐control (which underpins harm reduction‐based interventions). As part of the review process, we examined each of the included studies to assess whether and what theoretical justifications were provided for the intervention. This assessment is set out in the findings section.

### 2.6 Why it is important to do this review

Homelessness is a significant and growing social and public policy issue in many high‐income countries, where rates of all kinds of homelessness have increased over the last quarter of a century. Homelessness has a devastating effect on those experiencing it, on the wider community, and is costly for public services. There is disagreement around which interventions are most effective in preventing and reducing homelessness, particularly in relation to people experiencing more visible and extreme forms of homelessness (such as street homelessness). People experiencing street homelessness in particular also face issues around problematic substance use, offending behaviour, and/or mental health issues, and make extensive and expensive demands on public services, notably in health (Aldridge, 2018) and criminal justice, in part because people experiencing street homelessness are considerably more likely than the general population to be victims of crime (O'Leary, 2004). People experiencing homelessness often face issues accessing services (Cornes, 2018), and that evidence of the effectiveness of interventions is somewhat mixed (Luchenski, 2018).

Policy makers interested in using the evidence to determine whether and what types of interventions are most effective therefore face considerable challenges in navigating and interpreting the extant effectiveness evidence base. This systematic review aimed to provide a single synthesis of the evidence base to aid policy makers in their decisions.

## 3 OBJECTIVES

This systematic review identified, appraised, and synthesised studies that evaluated the impact of programmes aimed at stopping or reducing substance use in people experiencing homelessness. We aimed to answer the following research questions:

(1) How effective are interventions designed to reduce substance use in adults who are experiencing homelessness compared to treatment‐as‐usual (TAU)?
(2) What is the effect of abstinence‐based interventions on substance use outcomes in adults who are experiencing homelessness?
(3) What is the effect of harm‐based interventions on substance use outcomes in adults who are experiencing homelessness?
(4) Are abstinence‐based interventions more or less effective than harm reduction‐based interventions?
(5) What is the effect of individual interventions designed to reduce substance use in adults who are experiencing homelessness, compared to treatment as usual and each other?
(6) How do participant and study characteristics moderate the effect of interventions designed to reduce substance use in adults who are experiencing homelessness? Specifically:
  - For which substances are interventions most effective?
  - For whom do the interventions work best?
  - Over what period of time are interventions most effective?
  - How does the length of follow up period moderate effectiveness?

The remainder of this report is structured as follows. Section Selection Criteria, 4 describes the methods used in the review. Section Data Collection and Analysis, 5 presents the results of the review, including an overview of the search results, the characteristics of included studies, the results of the critical appraisal and the meta‐analytic results. Section Main Results, 6 presents a discussion of the results, including in comparison to the previous literature reviews and Section Authors' Conclusions, 7 presents our conclusions for policy and practice.

## 4 METHODS

As set out in the protocol for this review (O'Leary, 2022), the methods used followed those described in the Campbell and Cochrane Collaboration guidelines to the production of systematic reviews (Higgins, 2022; Kugley, 2017). Our primary outcome of interest was substance use. The primary source of studies for potential inclusion in this review was the Homelessness Effectiveness Studies Evidence and Gaps Maps (EGM), specifically the 4th edition (Singh, 2022). We synthesised the included studies using meta‐analysis and meta‐regression, using a typology of interventions created at the beginning of the review, validated with an expert panel of academics, policy makers, experts by experience, and practitioners.

### 4.1 Criteria for considering studies for this review

#### 4.1.1 Types of studies

We included impact evaluations that employed study designs which included some form of comparison, which included all studies categorised as either ‘Randomised Controlled Trials’ or ‘non‐experimental designs with a comparison group’ from the studies in the EGM. Because of time and resource limitations, we only included studies published in English.

#### 4.1.2 Types of participants

There are a number of definitions of homelessness available, reflecting differences between countries and over time. There are also different forms of homelessness, taking into account the length of time someone has been experiencing homelessness, distinctions between living on the street or in their vehicles, or having a temporary place to stay. We used the definition of homelessness used in a recently published Campbell Collaboration protocol. This definition is:

‘Homelessness is defined as those individuals who are sleeping rough’ (sometimes defined as street homeless), those in temporary accommodation (such as shelters and hostels), those in insecure accommodation (such as those facing eviction or in abusive or unsafe environments) and those in inadequate accommodation (environments which are unhygienic and/or overcrowded). (Keenan, 2020, p. 13)

Our focus was on adults (men and women aged 18 years and over), undertaken in any high‐income country. Studies of families or children were excluded from the review. These were excluded because in many countries (particularly the UK), there are different legal frameworks that apply to homeless families and children, and thereby access to different types of services, and different outcomes expected. We did not include studies that included people below age 18, even if some of the population was equal to or older than age 18.

#### 4.1.3 Types of interventions

The review synthesised findings about the impact of a range of interventions aimed at reducing, stopping, or preventing problematic substance use or harms associated with problematic substance use. In developing the protocol for the review and drawing on the extant effectiveness literature, we created a typology of such interventions. This typology is set out in Table 1. The typology was discussed and validated with an expert panel of academics, policy makers, experts by experience, and practitioners involved in problematic substance use treatments targeted at people experiencing homelessness, held in November 2020. Interventions listed in the left‐hand column of the typology in Table 1 are abstinence‐based; interventions in the right‐hand column are based on harm reduction approaches:

- **Abstinence‐based** – interventions that *require* participants to abstain from substance use, or whose goal is to achieve abstinence from substance use.
- **Harm reduction** – interventions that seek to reduce the harm caused by substance use but which *do not require* abstinence.

Interventions listed in the centre column are often practiced using both abstinence‐based and harm reduction approaches. These interventions are relevant to addressing the first objective of this review (how effective are interventions designed to reduce substance use in adults who are experiencing homelessness), but were excluded from analysis intended to address the second objective around the effectiveness of harm reduction versus abstinence based interventions.

The evidence synthesised in this review relates to adults experiencing homelessness. Many of the interventions covered by this review are used more generally and with other populations, and as such there may be evidence which is not specific to adults experiencing homelessness. It is therefore possible that there is evidence that specific interventions work in general, but no evidence that they work for adults experiencing homelessness.

We provide an overview of the level of evidence in the included studies for each of the interventions listed in this typology in Table 5 in the results section.

The definitions of each of the interventions included in the typology can be found in Supporting Information: Appendix 1.

Interventions aimed at reducing problematic substance abuse can also be categorised descriptively by type. The review team identified three such categorisations, namely psychosocial, treatment through medication (pharmacological), and non‐medical interventions. These descriptive categories are not material to the analysis underatken or to addressing the review objectives, but were nevertheless useful.

We also extracted information on the experience of the comparison group in each individual study (e.g., no intervention, treatment as usual, wait‐list, other treatment).

#### 4.1.4 Types of outcome measures

We included impact evaluations that included a substance use outcome. Substance use is measured in a number of ways in primary studies, for example:

- Number of days per month substances are used
- Self‐reported use of drugs
- Self reported use of alcohol
- Results of drug testing

We identified a range of continuous and binary outcomes in the reviewed studies. Where possible, we converted these into the same metric (e.g., Hedges' *g*) for the purpose of meta‐analysis (Borenstein, 2010). Where effect sizes were converted from a binary to continuous measure, we undertook a sensitivity analysis to investigate the effect of the inclusion of studies with a converted effect size in the meta‐analysis (described further below).

Our primary focus of this review is on the effectiveness of substance use interventions in relation to substance use outcomes. In particular, we are interested in the relative effectiveness of abstinence based and harm reduction‐based interventions.

While the primary objective of abstinence‐based interventions is to reduce, stop, or prevent problematic substance use, this is not the primary objective of harm reduction interventions. These interventions do not place an abstinence requirement on individuals engaging in treatment, but can and do empower service users to make personal abstinence decisions. Nevertheless, many intervention studies that cover harm reduction interventions do measure substance use outcomes, often involving a direct comparison between harm reduction interventions of interest and abstinence‐based comparisons.

#### 4.1.5 Duration of follow‐up

We excluded studies which measured outcomes beyond 36 months.

#### 4.1.6 Types of settings

We included studies that took place in any type of setting that employed abstinence‐based or harm reduction‐based interventions. We included studies that took place in high‐income countries only, recognising the substantive differences between high‐ and low‐income countries in terms of access to resources and services, and drivers of homelessness (Magwood, 2020). We used the same definition of high‐income country as White's (2018) EGM, that is, high‐income countries according to the World Bank classification (World Bank).

### 4.2 Search methods for identification of studies

The primary source of studies for potential inclusion in this review was the Homelessness Effectiveness Studies EGM, published by the Campbell Collaboration and the Centre for Homelessness Impact. There have been several editions of this EGM, and the review here draws on the 4th edition (Singh, 2022), for which searches were completed in September 2021. We also conducted supplementary searches, specifically through a call for grey evidence and hand‐searching key journals.

#### 4.2.1 Electronic searches

White et al. (2021) describe the process for identifying and searching for the studies included in the EGMs and we do not repeat this here.

The review team shortlisted studies for this review by identifying primary studies and systematic reviews from the EGM (2020 and 2022 editions) (by filtering using the previously coded classifications) which appeared to meet the inclusion criteria for this review. Systematic reviews from the EGM were unpacked, and their constituent studies added to the shortlist for screening. We also identified another systematic review, Moledina et al. (2021), during the searching process.

#### 4.2.2 Searching other resources

In November 2020 the review team issued a call for grey evidence (with a deadline of 8th January 2021) which was disseminated through the review team's and review funder's social media channels, inviting people with lived experience, researchers, commissioners, service providers and wider stakeholders to submit relevant grey evidence for consideration in the review. Specifically, the call was for evidence that is:

(1) empirical, based on research that:
  (a) elevates the voice of people with experience of homelessness.
  (b) measures the impact of interventions (before and after, quasi‐experimental, randomised controlled trial [RCT]).
  (c) identifies the barriers to, and facilitators of, successful implementation of interventions.
(2) is about interventions aimed at reducing or stopping problematic substance misuse, using harm reduction or abstinence‐based approaches;
(3) is not published in a book or academic journal; and
(4) is specific to the UK, or England, Northern Ireland, Scotland or Wales.

We received eight studies in response to the call, none of which were impact evaluations or systematic reviews, and hence were not eligible for inclusion in the review.

The reviewers hand searched key journals, using similar search terms and date ranges as Singh and White (2022). While some may have already been searched as part of the evidence and gap map, this targeted journal search and more substance use and treatment focused search aimed to further ensure the capture of all existing literature and evidence. The hand searched journals included:

*Drug Alcohol Dependency*

*Psychiatric Services Journal*

*American Journal of Public Health*

*BMC Medicine*

*Journal of Substance Abuse Treatment*

*European Journal of Homelessness*

*Housing Studies*

*Social Policy and Administration*

*Addiction Research and Theory*

*Drugs Education Prevention and Policy*

*International Journal of Drug Policy*

*Addiction*

### 4.3 Data collection and analysis

#### 4.3.1 Description of methods used in primary research

We included studies that used a design rated at levels 3, 4 and 5 of the Maryland Scientific Methods scale, that is, experimental (randomised) studies and non‐experimental studies that included a baseline measure of the outcome. The studies measured the effectiveness of an intervention designed to reduce problematic substance use against another intervention or a control group (no intervention, treatment as usual, wait‐list).

#### 4.3.2 Data extraction and management

Two reviewers extracted data from eligible studies. This included details of the study, quantitative data required for meta‐analysis, and confidence in the study's findings (using the Campbell Collaboration's critical appraisal tool for primary studies – White et al., 2020). Coding disagreements were discussed and passed to the lead reviewer for resolution. We then extracted data for the following, where it was not already present in the EGM spreadsheet:

- Publication details (e.g., authors, year, source, study location)
- Intervention details, including basis (e.g., abstinence‐based or harm reduction‐based) and typology classification
- Substance and substance classification (e.g., alcohol, cannabis and synthetic cannabinoids, opiates and opioids, stimulants, CNS depressants, hallucinogens)
- Participant details, including classification (e.g., age, gender)
- Study design
- Comparison (e.g., other intervention, treatment as usual, waitlist control)
- Outcome description, definition and measurement (including measurement duration)
- Sample sizes of treatment and control groups
- Data to calculate Odds Ratios, Rate Ratios or Standardised Mean Difference (SMD)
- Confidence assessment

In addition, one reviewer examined each study to assess whether they did the following:

(1) Present a clear theory of change linking the intervention(s) being tested to substance use outcomes, and if so, what that was.
(2) Present an explicit justification provided for either abstinence‐based approach or harm reduction approach.
(3) Describe whether the intervention draws on social control theory, self‐control theory, both or neither (as defined in the previous section on how the intervention might work).

We defined a clear theory of change as presenting information on the following as a minimum, drawing on the Better Evaluation guidance (Betterevaluation.org, ND):

- Present an action theory: the authors present an explanation of intervention activities that need to be undertaken and level of success needed to produce the intended impact on substance use. There should be an explanation of why these activities have been designed as such and why the particular level of success is needed in order to see change and the final intended impact.
- Present a change theory: there is an explanation of one or more causal mechanisms by which change is intended to come about for individuals or groups (even if not presented explicitly as a ‘causal mechanism’).

#### 4.3.3 Assessment of risk of bias in included studies

Studies selected for this review were assessed for confidence of findings using a critical appraisal tool for primary studies developed by the Campbell Collaboration for use with maps and reviews. We used this tool over the Cochrane risk of bias as this was the tool used by White et al. (2019) to rate the confidence findings in their EGM, the primary source of studies for our review. Details of these tools and the assessment process are set out in White et al. (2019), and the tool itself can be found in Supporting Information: Appendix 2. The tool for primary studies has seven items which relate to (1) study design, (2) blinding, (3) power calculations, (4) attrition, (5) description of the intervention, (6) outcome definition and (7) baseline balance. Each of these seven items is rated as implying high, medium or low confidence in study findings. Overall quality is assessed using the ‘weakest link in the chain’ principle, so that confidence in study findings can only be as high as the lowest rating given to any of the critical items.

For studies that came from the EGM, we used the critical appraisal results undertaken by the EGM authors, with the exception of two cases where we disagreed with the original rating. For those cases, we agreed with the review funder that we would present our rating in this review instead of the original rating. However, we also identified a number of studies from our additional searching that needed critical appraisal. The team undertook independent critical appraisals of 25% of the newly included studies in order to make sure that everyone was interpreting the criteria in the tool in the same way. This process demonstrated that the team were interpreting the criteria in the same way. For the remaining studies, each study was coded by one person with checking of results by another team member.

#### 4.3.4 Measures of treatment effect

The included studies reported on continuous, ordinal, dichotomous and count‐based outcome measures. We calculated most standardised effect sizes using the David‐Wilson Practical Meta‐Analysis Effect Size Calculator (Wilson, 2019). Included studies presented the following types of statistical information that we extracted to calculate standardised effect sizes:

(1) To calculate a SMD for continuous data: means and SDs, means and SEs, unstandardised regression coefficients and SDs/SEs or 95% confidence intervals (CIs), results from *f*‐tests, results from *t*‐tests.
(2) To calculate odds ratio and risk ratio for binary outcomes: binary proportions, logistic regression coefficients and SEs or 95% CIs.

Three of the included papers, the authors did not present the rates of substance use in the treatment and control group, only their own rate ratios. We are unaware of a formula to convert a rate ratio to other types of effect size and conceptually we do not consider these comparable. Therefore, we present these studies individually and do not include them in any meta‐analysis. Therefore, for these studies, we have reported author‐calculated rate ratios and associated 95% CIs only, that is, the rate of an event in one group divided by the other. These papers were all associated with the At Home/Chez Soi RCT study in Canada.

**Figure 1:**
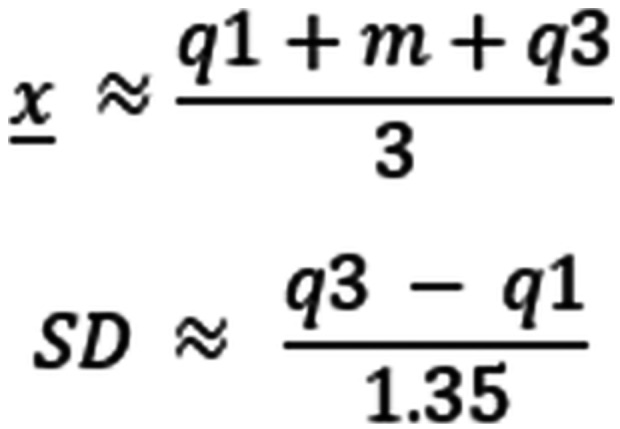


One study only presented medians and interquartile ranges (IQR) (Schumacher, 2000). In order to calculate effect sizes for these studies, we used the formula in Wan (2014) to calculate an appropriate mean and SD that was then used to calculate Hedge's *g*. This study was not included in any meta‐analysis. The formulas can be found below, where m is the median, is the approximate mean, q1 is the first quartile of the IQR and q3 is the third quartile of the IQR: (Figure 1).

**Figure 2:**
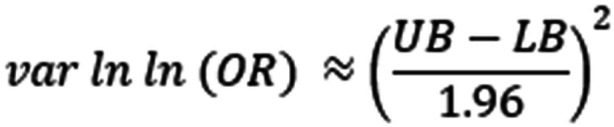


A few studies provided author‐calculated odds ratios with associated 95% CIs. In order to approximate the variance of the log(OR), needed to include the studies in a meta‐analysis, we used the following formula where UB and LB refer to the upper and lower bounds of the CIs of the log odds ratio: (Figure 2).

Finally, one of the studies reported results from a Poisson regression, a type of regression developed to model counts or frequencies. In this case, we used the Poisson Regression Effect size calculator to calculate both a Cohen's SMD and Rate Ratio.

In all cases, we compared the calculated effect sizes to the authors summaries of results to check that there were no significant differences between our effect size estimates and authors' conclusions. However, we found that it was common practice in this literature to consider only statistical significance of the results rather than magnitude of the effect with a discussion of statistical uncertainty that doesn't focus on a binary cut‐off. Therefore, in some cases we may report a moderate or large effect size with CIs crossing zero, despite the original authors reporting no effect on substance use.

**Figure 3:**
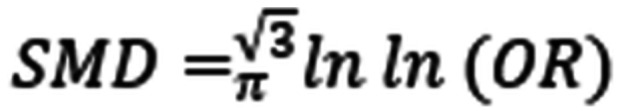


The majority of papers reported on outcomes based on a continuous scale and so the main effect size metric that was used for the purposes of the meta‐analyses was the SMD, with its 95% CI. Within this, Hedges' *g* was used to correct for any small sample bias. However, a number of studies reported on a binary outcome for which we were able to calculate a log odds ratio. We considered on a case‐by‐case basis whether it was sensible to convert the odds ratio to a SMD for the purpose of combining in one meta‐analysis, considering whether the binary outcome had been dichotomised from a continuous scale, for example, blood alcohol level. Where we did convert an odds ratio to a SMD, we relied on the assumptions described in the Cochrane handbook (Higgins, 2022) and used the following formula: (Figure 3).

Where we did not convert the Odds Ratio to a SMD, we present these results narratively in the results section.

As described in the Cochrane handbook, the standardisation of continuous outcomes into SMD does not correct for differences in the direction of the scale between studies. The majority of the included outcome scales used in the included studies in our review indicated a higher score or number for increased substance use, for example, days of drinking in the past month or score on the Addiction Severity Index. However, for others a decrease in substance use was indicated by a higher number, for example, number of days abstinent. We therefore multiplied the SMD and associated CI by –1 in these latter cases to ensure that all the scales point in the same direction.

#### 4.3.5 Unit of analysis issues

We assessed for unit of analysis errors in the included studies in the review. However, all but one of the studies in review randomised and analysed data at the individual level. Smelson (2018) was the only cluster randomised study, that randomised case workers and the group of people they worked with to a programme group or to an implementation as usual group. Their analytical approach accounted for clustering of observations within individuals and case managers and therefore we did not need to make any adjustments to the estimated standard errors in the included studies.

#### 4.3.6 Criteria for determination of independent findings

Dependent effects can occur when a study reports results for multiple measures of the same outcome construct for the same sample, the same outcome measure at multiple time points, when a study has multiple treatment arms compared to a common control group, or multiple studies evaluate the same programme and report on the same outcome. This is problematic as estimating an average effect using standard meta‐analytic models rely on the statistical assumption of independence of each included effect size (Gleser, 2007). Extracting data for all results can also significantly increase the amount of work.

Once we identified our pool of included studies, we mapped the programmes being evaluated, the outcome measures used in each study and the follow‐up time(s) to identify possible dependent effects. We implemented the following strategies to address dependent effects, drawing on (Piggot, 2020), with an aim to balance capturing as much relevant information from each study as possible with the limited timeframe for the review:

- Multiple follow‐up points: If a study reported results for the same outcome at multiple follow‐up points, we extracted data for a maximum of two time points that also meet the inclusion criteria of following up at 36 months or less and calculated standardised effect sizes for both. Reporting of multiple follow‐up points was very common in our pool of included studies (described in more detail in the results section). We extracted a short‐term follow‐up (the closest to the 6‐month follow‐up point) and a longer‐term follow‐up point. We extracted the closest result to a 6‐month follow‐up as this is a key point of interest for policymakers for these sorts of interventions. Given that multiple included studies reported more than two post‐6‐month follow‐up points, we mapped the longer‐term follow‐ups across all studies in the review and then extracted the longer‐term follow‐up point that was closest to the most common across each study (24 months).
- Multiple measures of the same construct: if a study reported results for multiple outcomes measuring the same or similar construct, we extracted one measure within two groups of outcome measures from each study: one self‐reported measure of substance use and one objective substance use measure (e.g., results of a drug test), using the most commonly used measures across the selected studies. Within these two categories of outcomes, if there were multiple self‐report measures or multiple objective measures in a single study, we extracted based on which is most common across the review. This is with the exception of measures broken down by type of substance. If a study presented a general measure of substance use, we extracted that over individual measures of specific substances that may also have been used. However, if a study only presented multiple measures of substance use broken down by type of substance, we extracted all data. We included in the same meta‐analysis using robust variance estimation (as described above) and explored variation by type of outcome measure.
- Multiple studies on the same programme: if different studies reported on the same programme but used different samples (e.g., from different regions), we included all in the review and include both in the same meta‐analysis where possible, treating them as independent samples. This is provided that the effect sizes were measured relative to a different control group. If multiple studies report on the same outcome(s) with overlapping samples, we chose the study with the larger sample size for inclusion in the review.
- Multiple treatment arms compared to a common comparison group: if a study reported results for multiple treatment arms and all interventions met our inclusion criteria, we extracted data for all arms and include in the same meta‐analysis using robust variance estimation where possible.
- Multiple specifications: if a study reported multiple estimates using different specifications for the same outcome, we chose the one that the authors present as their primary estimate. In all cases, we prioritised the intention to treat estimate from RCTs where possible as a more conservative and realistic estimate of programme impact.
- Multiple papers on the same study: if we identify multiple reports on the same study (e.g., a journal article and a working paper), we will include the most recent version.

#### 4.3.7 Dealing with missing data

There were six papers that did not report sufficient statistical information to calculate standardised effect sizes for at least some of the outcomes of interest for this review. In all of these cases, we contacted the authors to obtain additional information. However, for all but one of the six (Essock, 2006) we did not receive the necessary data and therefore some of the effects from these studies are excluded from statistical analyses. We were able to calculate some effect sizes for some of the relevant outcomes for Braucht (1995) and Upshur (2015). We present the characteristics of these studies and results of the critical appraisal in the overview tables below. The six papers are:

Burnam (1995) – unable to calculate effect sizes. Study included narratively in section of report on residential rehabilitation.

Braucht (1995) – study states that it collected substance use outcome data, but only service use data reported. Study included narratively in section of report on ACT/ICM.

Clifasefi (2020) – unable to calculate effect sizes. Study included narratively in section of report on group work.

Essock (2006) – authors provided data and study included in meta analysis for all interventions, and for ACT/ICM.

Stahler (1995) – unable to calculate effect sizes. Study included narratively in the section of the report on residential rehabilitation.

Upshur (2015) – some missing outcome data, but able to calculate effect sizes for other outcomes. Study included in meta analysis for ACT/ICM.

#### 4.3.8 Assessment of heterogeneity

We report standardised effect sizes and CIs for each individual study (where possible) and for each meta‐analysis we were able to undertake, we manually inspected the forest plots for heterogeneity. Given that our datasets included multiple effect sizes clustered into studies, it was likely that effects belonging to the same study would be more similar to each other than effects for different studies. We therefore calculated *I* ^2^ using the two variance components from the meta‐analytic outputs, for the between‐cluster heterogeneity and the within‐cluster heterogeneity. The *I* ^2^ values indicate how much of the total variance can be attributed to the total amount of heterogeneity, or the sum of between‐ and within‐cluster heterogeneity. We also present the proportion of heterogeneity that we estimate can be attributed to between and within cluster heterogeneity separately. Finally, we also present the absolute estimates for the two variance components, for the between‐cluster heterogeneity () and the within‐cluster heterogeneity ().

#### 4.3.9 Assessment of reporting biases

Our search strategy included searches for grey literature, which should help to mitigate any publication bias which might be observed if we were to only include published studies (as published studies are likely to report larger than average effects; Borenstein, 2010). However, we also undertook additional analysis to assess whether publication bias was likely to be a factor in our findings. This included a funnel plot to determine whether the summary effects in the meta‐analysis are subject to publication bias, and if this appears to be the case, further tests (e.g., Duval and Tweedie' Trim and Fill) to determine a ‘best estimate of the unbiased effect size’ (Borenstein, 2009, p. 286).

#### 4.3.10 Data synthesis

Our final set of studies and effect sizes were characterised by a range of types of dependencies, meaning a complex data set containing correlated effect sizes and sampling errors. Many studies reported effects for multiple measures of substance use and reported effects across multiple follow‐up times, or both. Several studies included multiple treatment arms compared to a common control group. Several studies, most notably the Chez Soi study, which was conducted across five cities in Canada, were reported on in numerous papers that referred to subsets of the population or individual city level results in the case of Chez Soi. Because of this variation of types of outcomes within studies, there was reason to expect that there would be within‐study heterogeneity in effect sizes.

For this reason, we used the Correlated and Hierarchical Effects (CHE) model (Pustejovsky, 2022) in combination with Robust Variance Estimation, using the metafor (Viechtbauer, 2010) and clubSandwich (Pustejovsky, 2022) packages in R. This model assumes that there is a constant correlation among groups of effect sizes from the same study, and also allows for between‐study and within‐study heterogeneity in true effect sizes. We did not have access to information on the likely correlation among effect sizes in our papers, and so we used an assumption of rho = 0.6 in all meta‐analyses, and tested the sensitivity of the results to higher and lower values of rho.

We present the results of the meta‐analysis on forest plots. In the cases where we include a large number of effect sizes within the same analysis that are dependent, we present pooled estimates that are aggregated to the study level for the forest plots, as the full set of individual effect sizes were not legible.

#### 4.3.11 Subgroup analysis and investigation of heterogeneity

We did not have a sufficient number of studies and effect sizes to include all our moderators of interest in the same analysis, which we pre‐specified as our preferred approach in the protocol. We therefore estimated single variable meta‐regression models for some of our moderator of interest and interpret each of these with caution. Before doing this, we explored the relationship between our moderating variables of interest in our pool of included studies to understand if any were correlated.

We used a no‐intercept specification so that coefficients represent average effect sizes for the corresponding category, as well as testing the difference between moderating variable values using an intercept model.

#### 4.3.12 Sensitivity analysis

Where possible, we undertook sensitivity analysis to determine the effect on our overall findings of:

- Non‐randomised studies
- Studies with effect sizes which have been converted from binary to continuous
- Studies classified as low confidence in findings

We undertook this sensitivity analyses by repeating the meta‐analysis, omitting in turn each of the groups of studies described above, to determine their effect on overall findings.

#### 4.3.13 Treatment of qualitative research

We did not include qualitative research in the review.

[1] https://www.betterevaluation.org/en/managers_guide/step_2/describe_theory_of_change

[1] Level descriptions taken from https://whatworksgrowth.org/resources/the-scientific-maryland-scale/

## 5 RESULTS

### 5.1 Description of studies

#### 5.1.1 Results of the search

We identified 328 papers out of a total 562 papers that were relevant from the EGM. In addition, we identified a total of 130 papers from supplementary searches of relevant journals and unpacking of systematic reviews and from our call for evidence. We excluded 259 papers at this title and abstract screening stage, meaning that we progressed 199 papers for full‐text screening. We attempted to identify full texts for all of these, however we were unable to identify the full‐text PDFs of six papers.

We excluded 145 of the 193 papers screened at full‐text. We recorded the first reason that we identified for exclusion. The most common reasons for exclusion were that the papers did not evaluate an intervention in our typology (*n* = 49), they did not target our population group of interest (*n* = 12), they did not use an RCT or quasi‐experimental design (*n* = 33) or they did not measure a substance use outcome (*n* = 45). In addition, we identified five papers that reported on ongoing studies and therefore were not eligible for inclusion, one paper that was published before 1990.

**Figure 4:**
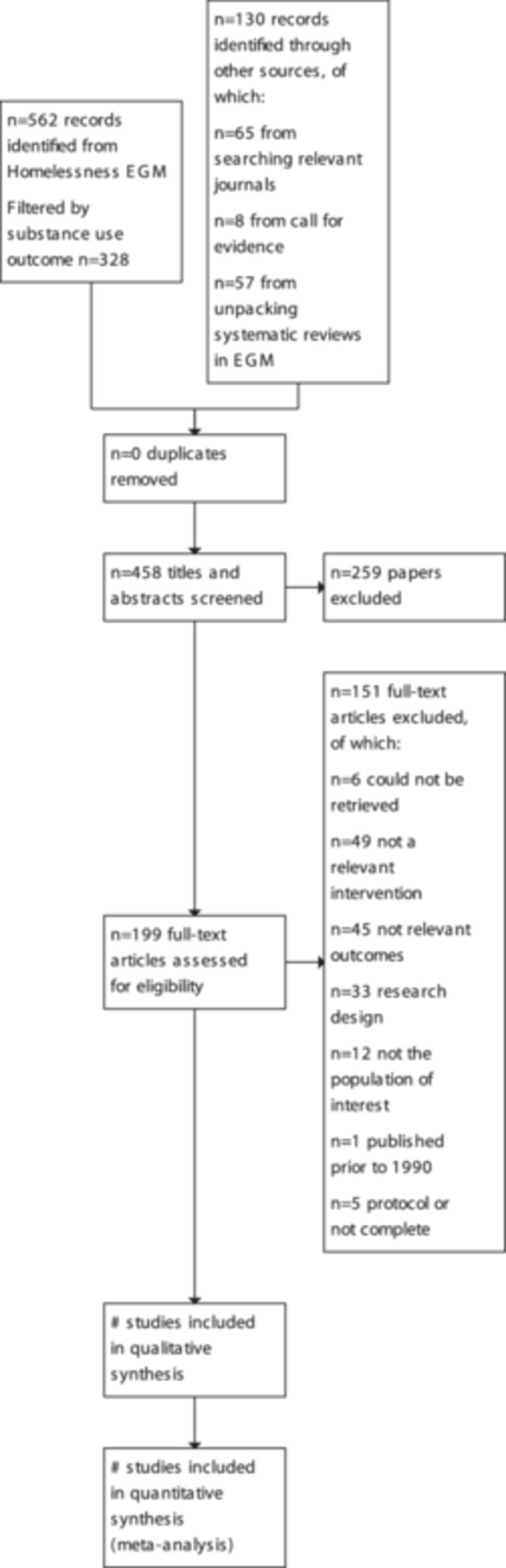
The PRISMA diagram.

This left us with 48 included papers in the review. These papers correspond to 34 unique studies. The overall process and outcome of the search and screening process is set out in Figure 4.

#### 5.1.2 Included studies

Forty‐eight papers, covering 34 separate studies, were included in this review. Table 2 provides a descriptive account of the 48 included papers.

**Table 2:** Types of comparisons in the included studies.

Type of comparison	Number of papers	Relevant research questions
Abstinence‐based intervention vs. abstinence‐based TAU comparison	7	1,2,5,6
Abstinence‐based intervention vs. abstinence‐based intervention (new interventions)	7	5
Abstinence‐based intervention vs. unknown TAU or new intervention condition	2	1,2,5,6
Harm‐reduction intervention vs. abstinence‐based TAU comparison	18	1,3,4,5,6
Harm‐reduction intervention vs. harm reduction TAU comparison	1	5
Harm‐reduction intervention vs. unknown TAU or new intervention condition	7	1,3,5,6
Harm reduction intervention vs. abstinence‐based comparison (new interventions)	4	4,5
Intervention group and comparison group unclear (comparison of a new intervention to another new intervention)	1	5
Unclear intervention group, abstinence‐based comparison (new intervention)	1	5
Total studies	48	

#### 5.1.3 Linked papers

Several studies were covered by more than one paper. Most notable of these is the Canadian At Home/Chez Soi Housing First with ACT/ICM, which is covered by nine separate papers. We have included one of these papers (Aubry, 2016) in our analysis, and the other papers are presented narratively in the findings section on research question 5 with respect to ACT/ICM.

Four papers examine a contingency management and day therapy intervention in Birmingham, Alabama. These four papers – Milby (2000, 2003); Schumacher (2000, 2003) – differ in terms of the outcomes and follow up periods covered. The four are included in analysis set out in the findings section on research question 5 with respect to contingency management interventions. Three papers are included in the meta analysis, and one Schumacher (2000) is presented narratively.

Two papers cover a study on the HUD‐VASH funded by the Department of Veteran Affairs in the United States. These two are Rosenheck (2003) and O'Connell (2012). Rosenheck (2003) is included in the meta analysis for the overall effectiveness of substance use interventions (research question 1) and also for research question 5 with respect to ACT/ICM, in which O'Connell (2012) is presented narratively.

Finally, three papers covered a study of variations of ACT in St. Louis, Missouri. These three papers – Morse (2006, 2008); Fletcher (2008) – differ in terms of follow up period, and Morse et al. (2008) includes a different treatment group. The two Morse papers are included in the meta analysis for research question 1; Morse (2006) is included in the meta analysis for research question 5 with respect to ACT/ICM, in which Morse (2008) and Fletcher (2008) are presented narratively.

#### 5.1.4 Characteristics of the evidence base

We included 48 papers in our systematic review, that report on 34 unique studies. In this section, we describe the characteristics of these papers and studies.

##### Settings

We included 37 papers that reported on 31 studies from the United States. We included ten papers that report on two studies from Canada. Nine of these papers from Canada report results from the same cross‐city RCT of the Chez‐Soi/At Home trial of Housing First combined with ACT or ICM (Aubry, 2016, 2019; Chung, 2018; Kirst, 2015; Somers, 2015, 2017; Stergiopoulos, 2015, 2016, 2019). These were included because both ACT and ICM are interventions with a primary substance use objective and involve direct service provision. We included one study from France (Tinland, 2020). None of our included studies took place in the United Kingdom.

##### Study designs

The evidence base for the systematic review consists mainly of RCTs. Twenty‐six (*n* = 26) of our included papers reported on an individually randomised, two‐armed parallel RCT, while 14 reported on a multi‐arm, individually randomised parallel RCT. Just one of our included papers was a parallel group cluster‐RCT, randomised at the level of case manager (Smelson, 2018), and we also included one cross‐over RCT (Tucker, 2017). We included six quasi‐experimental papers that included baseline data on substance use for the both the intervention and a comparison group (Cherner, 2017; Clifasefi, 2020; Harpaz‐Rotem, 2011; Morse, 2008; Stahler, 2005; Young, 2009) but did not use randomisation to allocate people to an intervention or control group. The quality of these studies is discussed further in the section on the critical appraisal.

##### Population

Our primary population of interest was people experiencing homelessness who were of the age of 18 or over, as described in our inclusion criteria section. Given our focus on interventions targeting substance use, most of our included studies also primarily focused on individuals experiencing homelessness and problematic substance use. We categorised 11 of our papers as stating that they worked primarily with people with problematic substance use and experiencing homelessness. Eight of the papers focused on people experiencing homelessness who were also experiencing mental ill health. One paper focused on working with veterans who were homeless and engaging in problematic substance use (Kashner, 2002), while another focused on working with men who have sex with men (MSM) and who were experiencing homelessness (Reback, 2010). However, a number of the included studies explicitly offered interventions to people with a range of needs or experiences and we categorised 27 papers as working with individuals facing multiple and significant challenges beyond their experience with homelessness and problematic substance use. This includes, for example, Conrad (1998) that offered residential rehabilitation to veterans who were homeless, engaging in problematic substance use, and also had diagnosed mental health issues.

We have only included studies that explicitly cover participants who are experiencing homelessness (or whose sample predominately includes people experiencing homelessness). Studies that focus on our interventions of interest but do not explicitly target adults experiencing homelessness will not have been included, and as such for some interventions explored here there is a wider effectiveness literature.

##### Intervention and comparisons

Our interest was in interventions with a primary objective of reducing, stopping or preventing problematic substance use or harms related to problematic substance use. We excluded from the study papers that were only about shelters and hostels, housing and supported housing interventions, as the primary objective of these interventions is to provide shelter. While several of the included papers do include accommodation‐based services, these were included because the study also covered an explicit substance use intervention. We also excluded studies where the intervention of interest involved case management only, unless the study explicitly stated the specific substance use intervention to which case managers referred research participants.

As discussed in the methods section, we aimed to categorise each paper by whether they tested abstinence‐based intervention(s) or harm reduction intervention(s) according to our definitions. We also aimed to do the same for the control groups. Seventeen of the included papers tested one or more abstinence‐based interventions according to our definition, while 29 tested a harm reduction intervention. We were unable to categorise two of the included papers and these are therefore excluded from our analysis that focuses on comparisons between these two categories of interventions. It is important to note that very few these studies explicitly described themselves as testing either an abstinence‐based or harm reduction‐based intervention, discussed further in the next section.

We categorised 16 of the papers as using a control group clearly offering primarily abstinence‐based services to the participants and two papers as offering a harm reduction‐based service to the participants. There were another 20 papers where the description of the control group appeared to be primarily abstinence based on the description or based on the team's knowledge of the context and therefore, we categorised these as having an abstinence‐based control group. There were 10 cases where we were unsure of whether the offer to control group participants was primarily abstinence‐based or harm reduction based and these are therefore excluded from our analysis that focuses on comparisons between these two categories of interventions. Thirty‐three of the included papers compared a substance use intervention to a TAU offer for people experiencing homelessness, while 15 compared a substance use intervention to a new intervention. The types and number of comparisons made in our included papers can be found in Table 2 below.

Our typology set out in Table 1 included 28 separate substance use interventions that are used in relation to adults experiencing homelessness. Of these 28, we were able to identify effectiveness studies in relation to nine individual interventions. The number of included effectiveness studies for these 9 interventions range from 17 for ACT, to one for group work. Table 3 sets out the number of included studies for each of the nine interventions for which analysis was undertaken, and whether this intervention was classified as harm reduction or abstinence based.

**Table 3:** Overview of primary intervention tested in included papers.

Intervention	Abstinance or harm reduction based	Number of papers
Assertative Community Treatment (ACT)	HR	17
Contingency management	AB	9
Group work	HR	1
Harm reduction psychotherapy	HR	2
Intensive Case Management (ICM)	HR	9
Motivational Interviewing	HR	2
Residential rehabilitation	AB	4
Talking Therapies (CBT)	HR	2
Therapeutic Communities	AB	2

##### Outcomes

We only included studies in our systematic review that included at least one measure of substance use. However, within this category there was a range of measures used, including self‐reported and objective measures. Common outcome measures included:

- GAIN‐SS Substance Use (Global Appraisal of Individual Needs– Short Screener)
- Addiction Severity Index (Alcohol or Drugs)
- Results of urine testing for alcohol or other substances
- Self‐reported number of days in the past × days using alcohol and/or other substances

In addition, included studies reported on a range of follow‐up points after the start of the intervention. As described in the methods section, we only extracted up to two follow‐up points, a short‐term follow‐up point (from 0 up to 6 months after the start of the intervention) and a long‐term follow‐up point (between 6 and 25 months since the start of the intervention).

##### Other

All of the 48 included papers were published in journals; we did not identify any includable dissertations, reports or books.

##### Use of theory in the included studies

As part of the review, we examined each of the included studies to explore whether they explained how the intervention might work. Each study was coded by the lead reviewer. This coding covered: whether an explanation was provided for how the intervention would work; whether a justification was provided for abstinence or harm reduction approach; and, whether the two theories of change outlined above – social‐control or self‐control – were used.

Of the 48 included studies, 10 provided a clear explanation of how the intervention might work. For example, Burnam (1995), in an examination of residential versus day abstinence‐based programmes, states that the intervention is based on the social model recovery approach, and includes a short description of the principles underlying the intervention. In an evaluation of an intervention that combined behavioural therapy and case management, Adeline Nyamathi and colleagues (Nyamathi, 2017) refer to the theoretical model underpinning the design of the intervention, and also refer to previous relevant effectiveness studies. Rebeck et al. (2010) include a sentence that describes how their intervention of interest – contingency management – might work.

Of these 10 papers, only 1 includes an explicit logic model. Kendon Conrad and colleagues (Conrad, 1998) provide a logic model and description of how the intervention that combines case management with residential treatment might work. The logic model diagram includes several statements about the rationale for change. The authors also reference their previous work on this intervention, which provides an in‐depth explanation of how the intervention might work.

Of the 10 included studies that provide a clear explanation of how the intervention might work, 4 are multi‐component interventions that include Housing First and a substance use intervention such as ACT or ICM. In each of these, an explicit explanation is provided for how Housing First might work, but not in relation to the substance use intervention. In most of these cases, substance use is a secondary outcome of interest, with primary outcomes being around housing stability.

Twenty‐six of the included studies provide references to previous effectiveness studies as explanation of how the intervention might work. Several of these refer to previous evidence by the study authors. A number of these studies also include multi‐component interventions that combine Housing First with a substance use intervention, and again the explanatory focus is on Housing First, though often substance use was a secondary outcome for these studies. Aubry (2016), Cherner (2017) and Chung (2018) provide different examinations of the Canadian ‘At Home/Chez Soi’ Housing First pilot; each provide specific references to the extant evidence base on the effectiveness of Housing First, but little explanation is provided around the relevant substance use intervention. In each case, the primary outcomes of interest are around housing stability, with substance use being a secondary outcome. There are a further 10 included studies that provide no explanation of how the intervention might work.

There were two further areas of interest in this analysis. First, the lead reviewer coded each study as to whether explicit justification was provided for taking a harm reduction or abstinence‐based approach. Only 2 of the 47 included studies provided such a justification, both with the same lead author, namely Susan Collins. In a paper about an intervention called HaRT‐A (Harm Reduction Treatment for Alcohol), Collins and colleagues (Collins, 2019) provide a detailed justification for taking a harm reduction approach, including reasons why such an approach is often preferred by people experiencing homelessness. In a second paper, focusing on a variation of the HaRT‐A intervention (Collins, 2021), explicit justification for the harm reduction approach is also provided.

Finally, the included studies were reviewed to assess whether they provide any reference to social control and self‐control as the underlying theory of change for substance use interventions. There was no evidence, from any of the included studies, to suggest support for the review team's hypotheses about these two theories.

Overall, the assessment set out here suggests that, in relation to the 48 studies included in this review, only a small number provide insight into how the substance use intervention of interest might work to reduce, stop, or prevent problematic substance use by people experiencing homelessness. Most of the studies included refer to previous effectiveness studies, and 10 provide no theoretical explanation at all. Based on these studies, the review team concludes that these interventions are under‐theorised in terms of the causal mechanisms that might lead to reductions in substance use for this population.

#### 5.1.5 Excluded studies

Of the 193 papers subjected to full review, 145 were excluded from the review. Most (*n* = 49) were excluded from the study because they did not evaluate an intervention of interest. In many cases, the intervention was not adequately described in the paper abstract, and it was not possible to determine whether the paper covered an intervention of interest without reviewing the article in full.

Forty‐five papers were excluded because they did not measure relevant outcomes, or did not measure outcomes specifically for adults experiencing homelessness. Again, the review team found that outcomes measured were often not included in the title and abstracts reviewed. Thirty‐three papers were excluded at full review because of research design issues, including lack of comparator for the intervention of interest. Nine papers were excluded because study participants were not aged 18 or over. A number of papers in the effectiveness EGM use terms such as ‘youth’, ‘young adults’, or ‘adolescents’ without being explicit in the abstract about the age range of participants. As there is no single agreed age range associated with these terms, full review was necessary to determine whether the paper met our age inclusion criteria. A summary of the reasons for exclusion is provided in Figure 4.

### 5.2 Risk of bias in included studies

As described above, we used a critical appraisal tool that rated each paper in the review on the study design, use of masking or blinding, use of power or sample size calculations, overall and differential attrition at each follow‐up point, clear presentation of the intervention being evaluated, definition and reliability of outcome measures and baseline balance on observable characteristics (White, 2019). The ratings on each of these criteria were then used to make a judgement about the overall confidence that we have in the findings of the paper, according to a set of decision rules. We report these results at the level of individual paper in the review (*n* = 48) rather than the study level (*n* = 34) as the rating may vary by paper. Twenty‐eight (*n* = 28) of the papers were appraised by the original EGM team while 20 papers which were not included in the original EGM were appraised by this review team.

**Figure 5:**
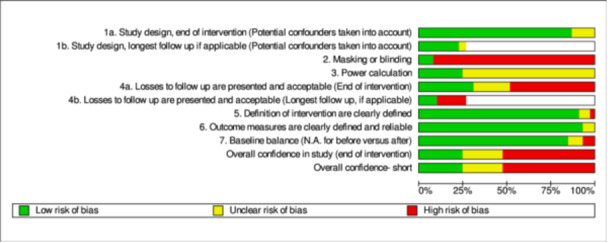
The Summary of risk of bias assessment by criterion.

Figure 5 presents an overview of the results of the critical appraisal for each criterion in the review. The majority of the papers were rated as low confidence (*n* = 25, or 52%). As described further below, by far the most common reason for studies being rated as low confidence was high rates of attrition and/or differential attrition of study participants, that fell below the What Works Clearinghouse (WWC) liberal attrition standard[1]. Eleven of the included studies were rated as medium confidence and 12 studies as high confidence.

The vast majority of papers included in our review were RCTs and therefore most were rated as high confidence on the use of a study design that took into account confounding factors (*n* = 42). Six studies used a quasi‐experimental approach (*n* = 6), for example, through the use of a comparison group matched to an intervention group with baseline data on substance use in both the intervention and control group, and were therefore rated as medium confidence on design. Most papers did not include information to suggest that their sample size was determined by power or sample size calculations (*n* = 36). The vast majority of papers did not report that they used masking/blinding of outcome assessors or masking/blinding of the team to the intervention allocation during analysis (*n* = 44) and were therefore rated as low on this criterion.

**Figure 6:**
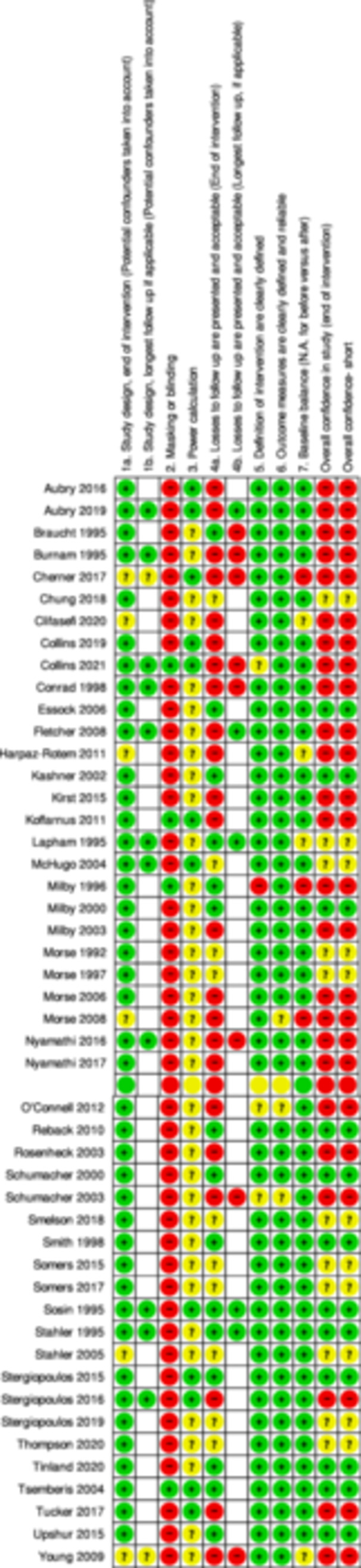
The study‐level risk of bias assessment.

Most of the included papers reported high rates of attrition (overall and differential between intervention and control) of participants. Given that we extracted data on up to two follow‐up points from each study (a short and a long‐term follow‐up point, depending on what was measured in the paper), we rated both follow‐up points on their overall and differential attrition. On the shorter‐term or first follow‐up point, 23 studies were rated as low confidence given losses to follow‐up, 9 studies (*n* = 10) were rated as medium confidence given losses to follow‐up and 15 as high confidence. At the longer‐term follow‐up point (not measured in all papers), 8 out of 12 papers were rated as low confidence due to the loss to follow‐up and 4 out of 12 as high confidence (not shown in the Figure 6 below).

Almost all of the studies were rated as high confidence in terms of their description of the intervention being tested (*n* = 44) and their clear definition of a substance use outcome that also referenced validation (*n* = 45). The three studies that were rated as being of medium confidence on the outcome measures criterion provided a brief description of the measure but did not present clear information on how the outcome measure had been previously validated or demonstrated to be reliable. Finally, 41 of the 48 papers reported balance on observable characteristics between the intervention and control groups at baseline (*n* = 41) and therefore were rated as high confidence on this criterion.

### 5.3 Assessment of reporting biases

We explored the potential presence of publication bias by producing a funnel plot for the studies that were included in our analysis for research question 1, that is, 45 effect sizes from 15 studies reported in 16 papers that evaluated the question of how effective were interventions designed to reduce substance use in adults who are experiencing homelessness compared to TAU. A funnel plot displays the relationship between the effect size (on the x axis) and the size of a study (on the y axis), in this case using the standard error of the effect size. The use of different colours on the plot is to indicate that effect sizes came from the same study.

**Figure 7:**
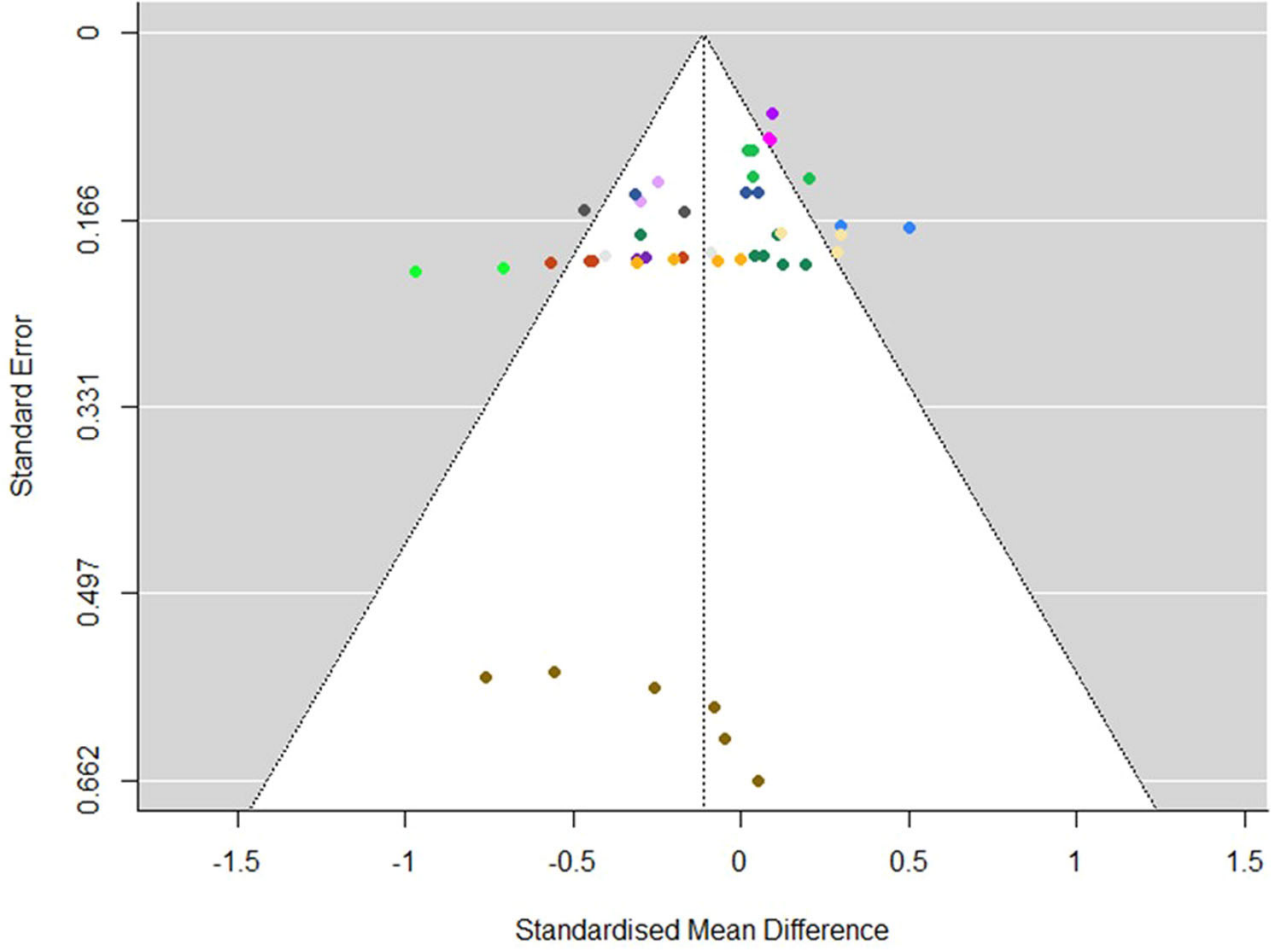
Funnel plot for substance use outcomes.

A collection of studies towards the bottom of the funnel plot on the right‐hand side with fewer on the left is typically used as evidence in favour of publication bias. Visual inspection of the funnel plot in Figure 7 does not indicate an obvious asymmetry towards either side of the plot. Therefore, the funnel plot does not indicate the presence of publication bias. However, we caution that the number of studies included in the analysis and therefore the funnel plot is low and a funnel plot alone is not conclusive evidence of no publication bias within the literature.

We ultimately only identified journal articles that met our inclusion criteria. Although we took steps to minimise the risk of missing unpublished and relevant studies for the review, we do recognise that there may be dissertations and theses, for example, which we have not located in this review. We therefore acknowledge that publication bias could potentially still be an issue. The steps we took to minimise the risks from reporting bias included an extensive search of sources of both published and grey literature and a call for grey evidence to identify studies that may have been missed by our search.

### 5.4 Synthesis of results

#### 5.4.1 Research question 1: How effective are interventions designed to reduce substance use in adults who are experiencing homelessness compared to TAU?

To answer our first research question, we looked at the subset of studies that compared any intervention in our typology to a TAU control group. There were a possible 34 included papers, corresponding to 24 studies, that were relevant for this analysis. From these, we were able to combine 45 effect sizes from 15 studies reported in 16 papers, using a CHE meta‐analysis with cluster robust estimation. There were a number of papers and studies that we did not include in the meta‐analysis, for the reasons below:

- We were unable to calculate an SMD for Burnam (1995), Stahler (1995) and Clifasefi (2020) and these three studies therefore are not included in this analysis (the full list of studies is presented in the Missing data section in the methods section. We did not have a measure of variance for Smelson (2018) so could not include in the analysis).
- We only include one paper from the At Home – Chez Soi study in the meta‐analysis, Aubry (2016), which combines results for all the cities involved in the trial. The results from the other eight papers included in the review, which break down results by city or by sub‐group, are presented narratively in the section on the impact of ACT and ICM on substance use.
- We do not include Upshur (2015) as the authors present change from baseline results only which should not be combined in a meta‐analysis with other studies reporting comparison of post‐intervention values, as recommended by the Cochrane handbook.
- We do not include Braucht (1995) as the only outcome that we were able to calculate effect sizes for measured service use rather than substance use directly, specifically, the number of services that clients received. We report this result narratively in the section on ACT and ICM.
- We do not include O'Connell (2012) or Fletcher (2008) as these are associated longitudinal papers to Rosenheck (2003) and Morse (2006, 2008) respectively. For both these studies, we had already included a long‐term follow‐up point in the meta‐analysis. We therefore report results of these two papers narratively in the section on ACT and ICM.

Finally, we did not think it was appropriate to convert odds ratio to SMD for Nyamathi (2016) or Thompson (2020) and so these two studies are not included in the meta‐analysis. These results are presented narratively in the sections on ICM and motivational interviewing respectively.

**Figure 8:**
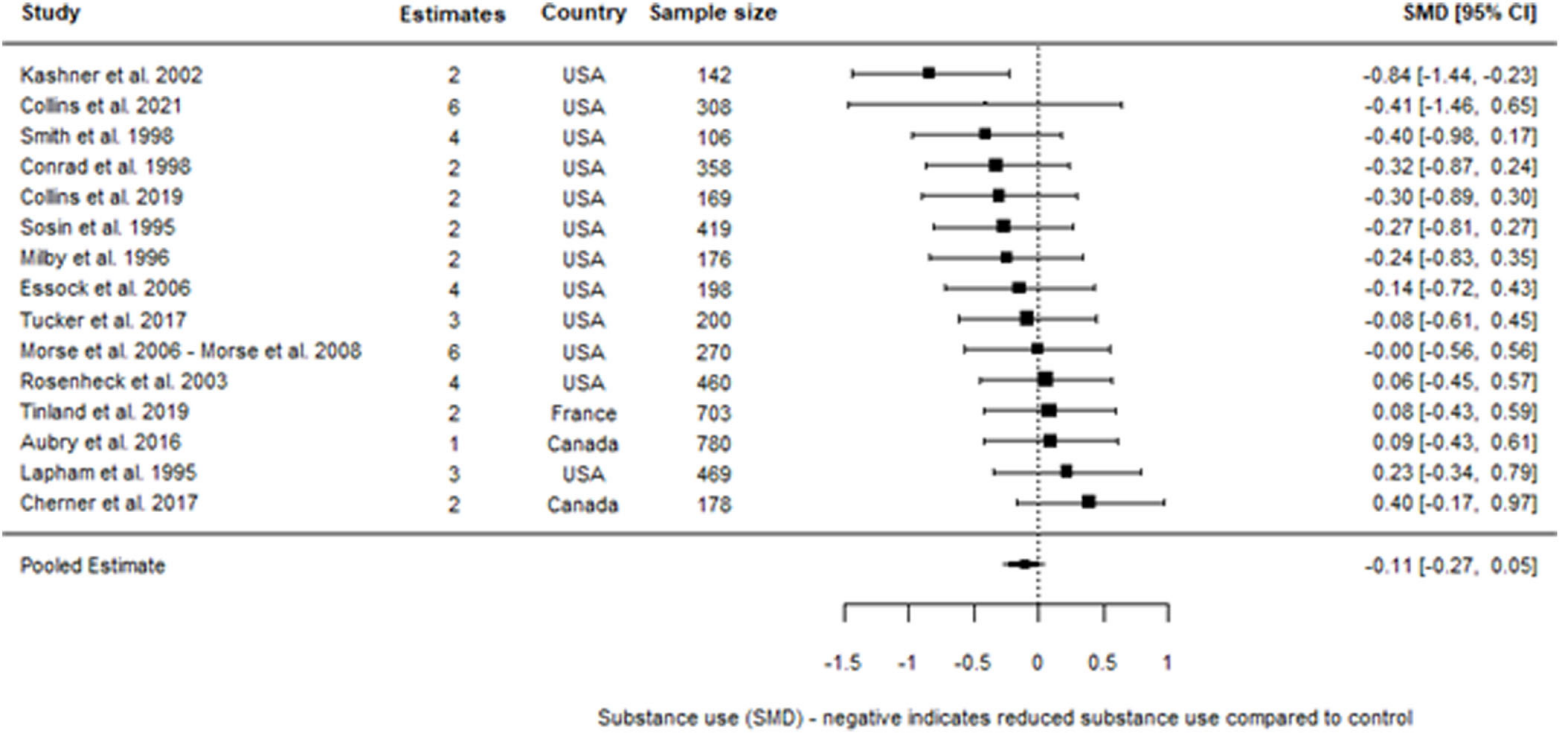
Average effect of homelessness interventions on substance use compared to TAU service provision.

Figure 8 below shows the meta‐analysis results to answer research question 1. Please note that we have averaged the individual effect size from each study for the purposes of displaying on the forest plot as there were too many individual effects to display clearly. Estimates refers to the number of effect sizes included for each study in the meta‐analysis. The mean number of effect sizes per study was 3 with the number of effects ranging from 1 to 6. Multiple effect sizes from single studies resulted from multiple follow‐up periods, multiple treatment arms against a common control and multiple measures of the same construct (substance use). All of the included studies were from the USA, with the exception of the two studies from Canada (Aubry, 2016 – the at Home/Chez Soi programme; Cherner (2017), a combined housing first and ICM programme in Ottawa) and study from France (Tinland, 2020). They tested a range of types of interventions from our typology, covering ACT, CM, Residential Rehabilitation, Motivational Interviewing techniques combined with a range of other interventions and ICM. The sample size column indicates the total number of people involved in the study across intervention and control group at baseline.

We calculated an average effect of –0.11 SD (95% confidence intervals [CI], −0.27, 0.05), indicating on average a reduction in substance use for people experiencing homelessness that participate in the included programmes compared to people experiencing TAU service provision. However; there is substantial heterogeneity across studies, which can be observed by looking at the forest plot. We calculated prediction intervals, that is, the expected range of true effects in similar studies in future settings (IntHout et al., 2016). We estimated these to be –0.11 SD (−0.69, 0.46), which contain a much larger range of treatment effects than the CIs, with a range of values on both sides of the line of no effect (indicating possible reduced or increased substance use as a result of being involved in these types of programmes). This heterogeneity is to be expected given the range of interventions being tested, the complex diversity of needs of the people involved across different studies and the mix of follow‐up periods (explored further below). Unfortunately, due to a limited number of included effect sizes and studies, we were unable to include these possible explanatory variables as moderators in the same analysis.

The *I* ^2^ value in this context indicates how much of the total variance can be attributed to the total amount of heterogeneity, which is the sum of between‐ and within‐cluster heterogeneity. We calculated an *I* ^2^ value of 71%, with 61% due to between‐study heterogeneity and 10% from within‐study heterogeneity (and the remaining 29% from sampling variation).

#### 5.4.2 Sensitivity analysis

We tested the sensitivity of the results of this meta‐analysis to:

(1) Different values of rho (the correlation among groups of effect sizes)
(2) Removal of low confidence studies
(3) Removal of quasi‐experimental studies
(4) Removal of studies where an effect size had been converted from a binary to a continuous outcome

We found that the point estimate and CIs were not sensitive to a range of values of rho (from a very low value of rho of 0.05 up to a very high correlation of 0.80).

However; the results were sensitive to the removal of low confidence studies (−0.21 SD, 95% [−0.59, 0.17] − 6 studies, 17 effect sizes), the removal of quasi‐experimental studies (−0.14 SD, 95% CI [−0.30, 0.02] − 14 studies, 41 effect sizes) and the removal of studies where an effect size had been converted from a binary to a continuous outcome (−0.08 SD, 95% CI [−0.31, 0.15] − 10 studies, 31 effect sizes). This suggests that the findings are sensitive to the inclusion of lower quality studies, although unusually the average effect increases when we remove the lowest confidence studies, as well as the choices we made on how to calculate effect sizes.

As explored in the next section however, we also found that the papers that were rated as low confidence were also more likely to take a harm reduction intervention approach. We therefore cannot be sure whether the intervention approach, the strength of the study approach, or indeed another factor, is contributing to variation in outcomes.

Research questions 2 and 3: What is the effect of abstinence‐based interventions on substance use outcomes in adults who are experiencing homelessness? What is the effect of harm‐based interventions on substance use outcomes in adults who are experiencing homelessness?

#### 5.4.3 Research questions 2 and 3: What is the effect of abstinence‐based interventions on substance use outcomes in adults who are experiencing homelessness? What is the effect of harm‐based interventions on substance use outcomes in adults who are experiencing homelessness?

To answer our second and third research questions, we used the same set of studies that we used to answer RQ1, that is, those studies that compared an intervention in our typology to a TAU control group. All but two of these studies compared an intervention to a TAU condition that we determined to be primarily an abstinence‐based offer of typical services for people experiencing homelessness who were also engaging in problematic substance use. In two cases, we were unable to categorise the control condition as harm reduction or abstinence based because of insufficient information (Essock, 2006; Tucker, 2017).

We were able to combine 45 effect sizes from 15 studies reported in 16 papers, using a CHE meta‐regression with cluster robust estimation. We included the categorisation of the intervention as either harm reduction based or abstinence‐based in the meta‐analysis as a moderating variable. We estimate a no intercept model, so that both harm and abstinence are included in the model as predictor variables. The studies excluded from this analysis are reported in the previous section addressing research question 1.

We caution that these estimates are based on a small number of effect sizes and studies. The studies are also diverse within both the abstinence and harm reduction categories, in terms of intervention and populations. As stated above, most of the harm reduction studies were rated as being of low confidence and the results from these studies are therefore to be treated with more caution. In addition, the most common intervention tested in the harm reduction category was ACT and ICM (six of the nine included studies). Therefore, we interpret the meta‐analysis results that answer research question 3 on the effect of harm reduction interventions compared to treatment as usual with some caution, examined in more detail in the discussion.

**Figure 9:**
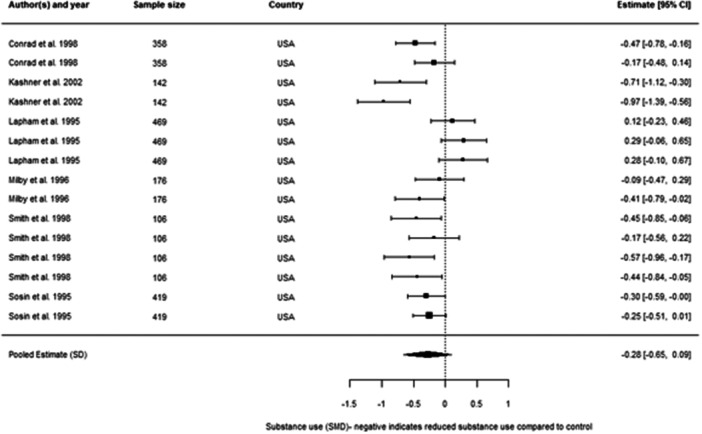
Average effect of abstinence‐based interventions on substance use compared to TAU service provision.

**Figure 10:**
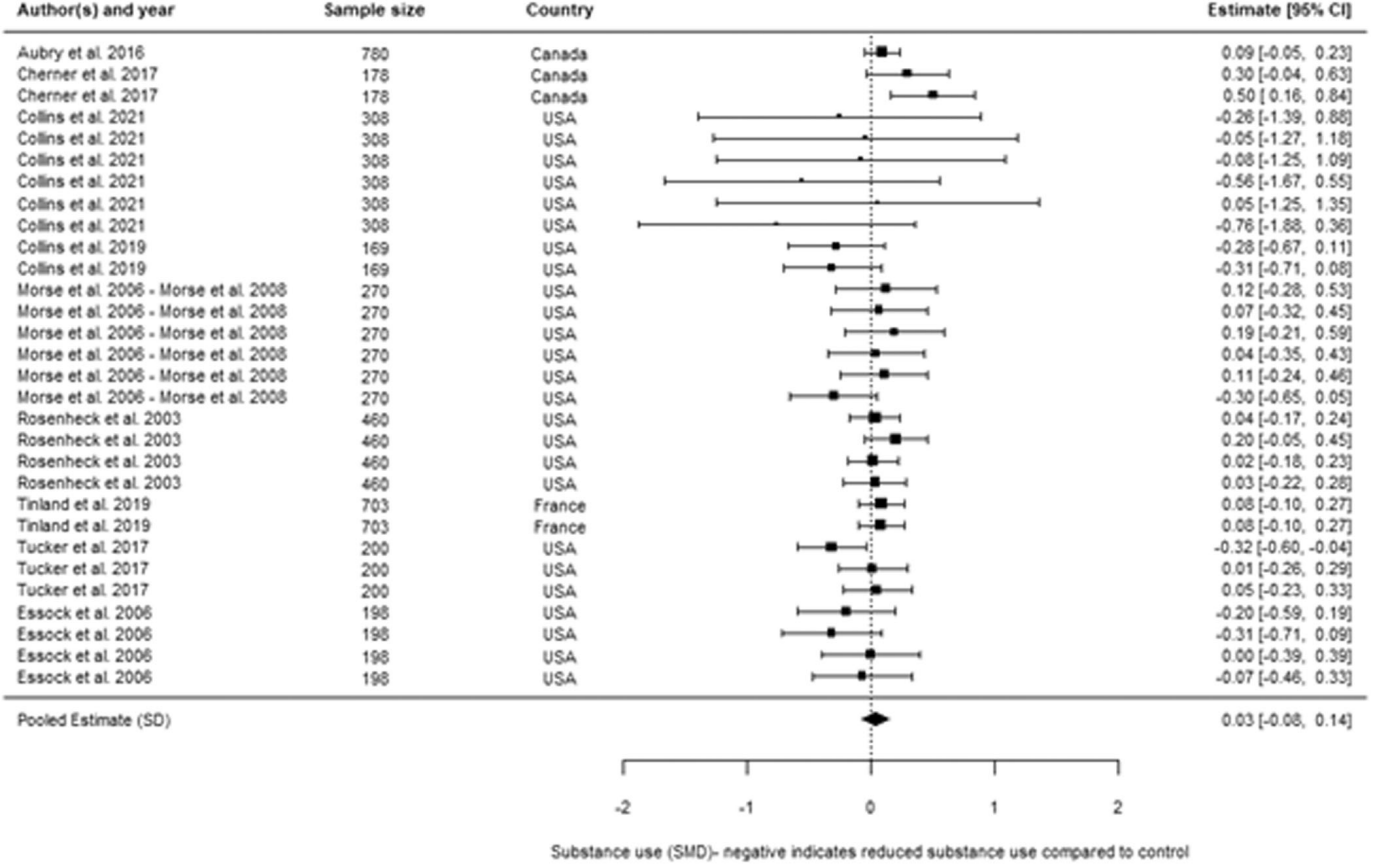
Average effect of harm reduction‐based interventions on substance use compared to TAU service provision.

The average effect for the abstinence‐based interventions included in this study compared to TAU service provision is –0.28 SD (95% −0.65, 0.09) (6 studies, 15 effect sizes), while the average effect for the harm reduction interventions included in this study compared to a TAU service provision is close to 0 at 0.03 SD (95% CI, −0.08, 0.14) (9 studies, 30 effect sizes). The CIs for both estimates are wide and crossing zero. For both of these analyses, the TAU comparison groups in the included studies are for the most part primarily abstinence‐based, with the exception of two studies where the comparison group condition was unclear (Figures 9 and 10).

#### 5.4.4 Sensitivity analysis

We intended to do the following types of sensitivity analysis:

(1) Different values of rho (the correlation among groups of effect sizes)
(2) Removal of low confidence studies
(3) Removal of quasi‐experimental studies
(4) Removal of studies where an effect size had been converted from a binary to a continuous outcome

As we found with the previous analysis, we found that the point estimates and CIs were not sensitive to a range of values of rho (from a very low value of rho of 0.05 up to a very high correlation of 0.80). We found little difference in both point estimates when we removed studies that had been converted from a binary outcome to a continuous outcome (10 studies, 31 effect sizes in the analysis), but found wider CIs (abstinence‐based interventions vs. TAU: −0.30 SD [95% CI: −0.96, 0.36]; harm‐reduction based interventions vs. TAU: 0.05 SD [95% CI: −0.13, 0.23]).

Because of the large number of harm reduction studies that were rated as low confidence we are unable to undertake sensitivity analysis by confidence rating for this set of papers. The overview of included studies in this analysis by confidence rating is shown in Table 4 below. The point estimate for abstinence‐based interventions was not sensitive to the removal of low confidence studies, however the CIs became wider (−0.28 SD [95% CI, −0.96, 0.40]).

**Table 4:** Number of effect estimates (studies) by type of intervention and critical appraisal.

	High or medium confidence	Low confidence	Total
Harm reduction interventions	6 (2)	24 (7)	30 (9)
Abstinance based interventions	11 (4)	4 (2)	15 (6)

The only two quasi‐experimental papers in the analysis were harm reduction‐based (Cherner, 2017; Morse, 2008); that is, all abstinence‐based studies were RCTs. The harm reduction point estimate changed by a very small amount following the removal of the quasi‐experimental estimates from the meta‐analysis (−0.02, 95% CI [−0.14, 0.10]).

#### 5.4.5 Research question 4: Are abstinence‐based interventions more or less effective than harm reduction‐based interventions?

We had intended to answer this research question using studies that made a direct comparison between the introduction of a new abstinence‐based intervention and a new harm reduction intervention. However, only four studies made a direct comparison between a new harm reduction intervention and an abstinence‐based intervention; McHugo (2004), Koffarnus (2011), Tsemberis (2004) and Young (2009). Three of these studies evaluate the impact of ACT compared to another intervention that was abstinence‐based, while Koffarnus (2011) evaluate several approaches to contingency management. Because of the limited number of studies, most of which are restricted to the same type of intervention, we were unable to draw conclusions about whether in general abstinence‐based interventions are more or less effective than harm reduction approaches. These studies are instead discussed in the following section on the effectiveness of ACT and contingency management.

There are 18 papers that compared a harm‐reduction based intervention to an abstinence‐based TAU, as reported in the previous section. However, we do not consider these studies to reasonably answer the question of whether abstinence‐based interventions are more or less effective than harm‐reduction based interventions. Although we determined that the TAU offer was primarily abstinence‐based, it is not clear from many of these papers whether the comparison group participants chose to take‐up any abstinence‐based services. In addition, the abstinence‐based treatment as usual condition often appeared to be a mix of different services, that were not consistently reported, and that may have also included some harm reduction‐based services that were not reported. While take‐up of the interventions in the four studies above comparing new harm‐reduction and new abstinence‐based treatments may have been mixed, in general take‐up of new interventions in these studies was higher. We therefore do not use the results of this set of studies to answer the question of whether abstinence‐based approaches are more or less effective than harm reduction‐based approaches.

#### 5.4.6 Research question 5: What is the effect of individual interventions designed to reduce substance use in adults who are experiencing homelessness, compared to treatment as usual and each other?

So far, we have examined the effectiveness of all substance use interventions compared with treatment as usual, the effectiveness of abstinence based and harm reduction‐based interventions. These analyses provide useful insights, particularly that interventions tend to be more effective than treatment as usual.

In this section, we set out our analysis of the effectiveness of individual interventions. Table 5 gives an overview of the findings for each of the interventions listed in the typology of interventions set out in Table 1. There were nine individual interventions for which we were able to undertake analysis. For three interventions, we were able to undertake meta analysis: for ACT and intensive case management (presented together), and for contingency management. For the other six interventions, we provide narrative synthesis. For all of the other interventions listed in our typology in Table 1, we did not identify any relevant effectiveness studies and were unable to undertake meta‐analysis or narrative synthesis.

**Table 5:** Outcome of analysis by individual intervention.

Intervention	Number of papers	AB/HR	Findings
Assertative Community Treatment (ACT)		HR	Not better than TAU on average. Average effect on substance use of 0.03 SD, 95% CI [−0.07, 0.13].
Contingency Management		AB	On average reduction in substance use. Average effect of –0.47 SD (95% CI, −0.72, −0.21)
Group work		HR	Reduction in use indicated
Harm reduction psychotherapy		HR	Reduction in use indicated
Intensive Case Management (ICM)		HR	Not better than TAU on average. Average effect on substance use of 0.05 SD, 95% CI [−0.28, 0.39]
Motivational Interviewing (MI)		HR	Mixed results indicated
Residential rehabilitation		AB	No better than TAU indicated
Talking Therapies (CBT)		HR	Mixed results indicated
Therapeutic Communities		AB	Reduction in use indicated

It is worth noting again two issues that are relevant when interpreting Table 5. These are: firstly, the focus of this review is the effectiveness of substance use interventions for adults experiencing homelessness. Many of the interventions listed in the typology are used more generally (i.e., with populations other than adults experiencing homelessness), and therefore there might be effectiveness evidence not included here because is not specific to adults experiencing homelessness; secondly, a lack of effectiveness evidence does not indicate that the intervention is ineffective. It simply means that there is no evidence that met the inclusion criteria for this review.

The effectiveness evidence for nine of the interventions listed in our typology is as follows:

#### 5.4.7 ACT and ICM

Assertative Community Treatment was originally developed for patients with severe mental illness, providing personalised, high intensity, holistic and integrated multidisciplinary community care services. In its initial design, this intervention did not include substance use treatment; however, over the past two decades counselling and other approaches to reducing substance use have been integrated into ACT. ACT can include components that are abstinence or harm reduction based, although the latter is more usual. Because of this, we have assumed that intervention studies that cover ACT or ICM are harm reduction based, unless otherwise stated in the primary research. We also included evaluations of ICM, which are similar to ACT but tend to work with people with less severe needs. Given their similar approach, we analyse them together but also undertook moderator analysis to explore whether there is a difference between the two.

We looked at the subset of studies that assessed the impact of ACT or ICM interventions compared to a control group without these interventions offering either TAU or another new intervention. We included 27 papers reporting on 17 studies that assessed the impact of ACT or ICM. We were able to combine 36 effect sizes reported in 15 papers from 14 studies, using a CHE meta‐analysis with cluster robust estimation. We do not includeBraucht 1995 in the analysis as we were only able to calculate a standardised effect size for the outcome that looked at service use, rather than substance use. We do not include Nyamathi (2016) as we were unable to convert the odds ratios to SMDs. However, we present the findings narratively for these two studies below. Fletcher (2008) and O'Connell (2012) are follow‐up papers of studies included in the meta‐analysis (Morse, 2006; Rosenheck, 2003 respectively) and are also presented narratively below.

We included 9 papers that report on the At Home/Chez Soi trial in Canada, an RCT of Housing First combined with ACT or ICM, depending on the needs of participants. It took place across Montreal, Moncton, Toronto, Vancouver and Winnipeg, with delivery in each city including a focus on a different sub‐population of people experiencing homelessness with severe or moderate mental health needs. ‘Housing First’ involves provision of immediate access to subsidised housing without conditions for those experiencing homelessness, alongside accompanying support, in this case either ACT or ICM. We included the results of only one paper reporting on the At Home/Chez soi trial in Canada in the meta‐analysis, Aubry (2016), as this paper presents combined results across all five included cities. However, we also present the results of the other included papers narratively below.

Most of the studies included in the meta‐analysis took place in the USA, with the exception of Tinland (2020), which took place in France, and Aubry (2016) and Cherner (2017) which took place in Canada. Follow‐up ranged from 6 months up to 36 months.

We calculated an average effect of 0.04 SD (95% CI, [−0.07, 0.14]). This indicates that the average effect of ACT or ICM programmes on substance use is close to 0. However, there is some heterogeneity both within and across studies, which can be observed by looking at the forest plots. We calculated prediction intervals of 0.04 SD (−0.23, 0.31), which contain a larger range of treatment effects than the CIs, with a range of values on both sides of the line of no effect (indicating possible reduced or increased substance use as a result of being involved in ACT or ICM programmes). Previous effectiveness research has identified issues with fidelity with some programmes described as ACT, and that greater fidelity is associated with better outcomes (see e.g., a systematic review of the effectiveness of ACT for substance use disorders by Penzenstadler, 2019). It may be that differences in fidelity between studies explain some of the heterogeneity found here. The review team did not include intervention fidelity as an area of interest in this research.

**Figure 11:**
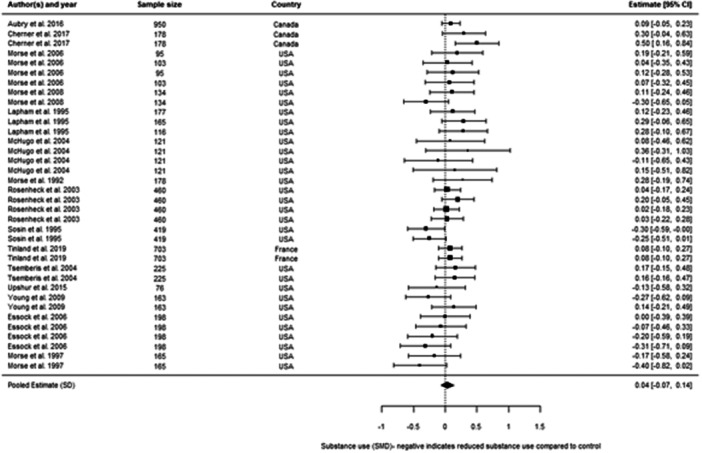
Average effect of ACT and ICM programmes on substance use.

We calculated an *I* ^2^ value of 34%, with 23% due to between‐study heterogeneity and 11% from within‐study heterogeneity (and the remaining 66% from sampling variation) (Figure 11).

When we undertake a moderator analysis to explore the individual effects of ACT or ICM, we see a very similar effect size to the combined average effect for the two types of programmes (ACT = 0.03 SD, 95% CI [−0.07, 0.13], 9 studies, 24 effect sizes; ICM = 0.05 SD, 95% CI [−0.28, 0.39], 5 studies, 12 effect estimates).

#### 5.4.8 Sensitivity analysis – ACT and ICM

We undertook the following types of sensitivity analysis for the meta‐analysis of ICM and ACT studies:

(1) Different values of rho (the correlation among groups of effect sizes)
(2) Removal of low confidence studies
(3) Removal of quasi‐experimental studies
(4) Removal of studies where an effect size had been converted from a binary to a continuous outcome

As we found with the previous analysis, we found that the point estimates and CIs were not sensitive to a range of values of rho (from a very low value of rho of 0.05 up to a very high correlation of 0.80). We found that the results were not sensitive to the removal of studies that had been converted from a binary outcome to a continuous outcome (13 studies, 34 effect sizes included for the analysis).

When we removed low confidence papers from the analysis (Aubry, 2016; Cherner, 2017; Morse, 2006, 2008; Rosenheck, 2003; Young, 2009), again the point estimate remains similar although the CIs become wider (0.00 SD, 95% CI [−0.17, 0.16], 9 studies, 21 effect sizes). Almost all the included studies were RCTs, with the exception of Cherner (2017); Morse (2008); Young (2009). The results were not sensitive to the removal of these three studies (0.03 SD, 95% CI [−0.07, 0.13], 12 studies, 30 effect sizes).

#### 5.4.9 Narrative synthesis – ACT and ICM

We included Braucht 1995 in the review, for which we were only able to calculate a standardised effect size for the outcome measure, number of services that the clients in the ICM group received. They tested an ICM approach which was offered to residents in a rehabilitation facility called Arapahoe House in the Denver area in the USA. Both intervention and control group were offered access to the full array of services offered by Arapahoe House, including detoxification, residential and outpatient services, and substance abuse counselling. The average age of participants was 35.4 years and 85% of the people involved in the study were male. The authors looked at the impact on service use as an outcome at 6 months after the start of the intervention, as well as stating they looked a range of substance use outcomes. The sample size at the beginning of the study was 323 people, with 163 people in the intervention group and 160 in the control group.

While the authors expected that case managers involved in the ICM programme would increase the number of services that the clients in the ICM group received, they did not find this to be the case, with a Hedge's *g* of 0.03 SD (95% CI, –0.20, 0.27). However, we rated this study as low confidence, rated down primarily on attrition.

As described earlier, we include nine papers in the systematic review that report on the At Home – Chez Soi RCT in Canada. We present the results of the papers not included in the meta‐analysis below. The pattern of results across these studies is a lack of a significant impact on substance use at any of the follow‐up points for which we extracted data. There are some effect sizes that indicate a small positive or negative impact on substance use for the intervention group compared to control but almost all have wide CIs that span the line of no effect. Substance use is measured in all papers using the Global Appraisal of Individual Needs (GAIN) Substance Problem Scale (SPS), with the exception of Somers (2015). This lack of impact is consistent with the combined results presented in Aubry (2016), which is included in the meta‐analysis above. None of these studies were rated as being of high confidence.

- Aubry (2019) presents results for Moncton only for ACT participants only at a 21–24‐month follow‐up period. They found a Hedge's *g* effect size of 0.01 SD (95% CI, –0.30, 0.31) on the GAIN‐SPS.
- Chung (2018) present results for all five cities included in the trial but focus on the impacts for older versus younger participants on the GAIN‐SPS. The authors calculate a ‘ratio of rate ratios’, which we present here as we were unable to calculate Hedge's *g* or an Odds Ratio from the presented data. For participants who were aged between 14 and 49, they find a rate ratio of 1.07 (95% CI, 0.93, 1.23). For participants who were aged 50 and over, they find a rate ratio of 0.79 (95% CI, 0.56, 1.11). They suggest there is no significant difference in impacts on substance use between the two groups.
- Kirst (2015) present results for Toronto only, looking at the combined impact for participants receiving both ACT and ICM at 6 months and 24 months. The authors present rate ratios, which we present here, where a rate ratio of less than one indicates lower substance use in the intervention group compared to control. At 6 months, the rate ratio for the GAIN‐SPS was 1.04 (95% CI, 0.83, 1.29). At 24 months, the rate ratio for the GAIN‐SPS was 0.84 (95% CI, 0.65, 1.13).
- Somers (2015) present results for Vancouver only, breaking down results by participants who received ACT and those who received ICM, at 24 months. They report impacts on the outcome ‘Less than daily substance use – derived using items from the Maudsley Addiction Profile’, where an Odds Ratio of more than 1 indicates reduced substance use in the intervention group. For the ACT group, they find an odds ratio of 1.22 (95% CI, 0.61, 2.45), and for the ICM group, an odds ratio of 0.78 (95% CI, 0.37, 1.63). They present both results as providing no evidence that results differed between intervention and control groups at 24 months.
- Somers (2017) present results for Vancouver only, presenting results at 24 months for participants who received ACT only, broken down by whether their housing was ‘Congregate’ Housing First or Scattered site Housing First. For the Congregate Housing First group, the Hedge's *g* effect size is 0.21 SD (95% CI, −0.06, 0.48), and for the Scattered site Housing First a Hedge's *g* effect size of 0.11 SD (95% CI, −0.18, 0.39). They present both results as providing no evidence that substance use differed between intervention and control groups at 24 months, although both effect sizes indicate slightly more substance use in the intervention groups compared to control.
- Stergiopoulos (2015) present results for Toronto only, focusing on the results for only ICM participants. At 6 months, the Hedge's *g* effect size is 0.04 SD (95% CI, −1.8, 1.87). At 24 months, the Hedge's *g* effect size is –0.08 SD (95% CI, −0.28, 0.12).
- Stergiopoulos (2016) present results for Toronto only, focusing on the results for the sub‐set of ICM participants who were an ethnic minority. In Toronto, the Housing First programme was adapted for ethnic minority groups specifically, with ICM services provided by a mental health agency using anti‐racist and anti‐oppressive frameworks of practice. The authors calculate a ‘ratio of rate ratios’, which we present here as we were unable to calculate Hedge's *g* or an odds ratio from the presented data. At 24 months, the ratio of rate ratios was 1 (95% CI, 0.61, 1.64), indicating no difference in substance use as measured by the GAIN‐SPS between the intervention and control groups.
- Stergiopoulos (2019) present results for Toronto, combining results for ACT and ICM participants and looking at a long‐term follow‐up point of 72 months. The Hedge's *g* effect size for this follow‐up period is –0.08 SD (95% CI, −0.14, −0.05), with a rate ratio of 0.92 (95% CI, 0.88, 0.95). They present these results as indicating no difference between the intervention group and control group during the 6‐year follow‐up period.

Fletcher (2008) is a longitudinal follow‐up of the study originally reported in Morse (2006). The authors report on an RCT testing two versions of ACT compared to a TAU comparison group for people experiencing homelessness with both severe mental health and substance use disorders. The authors test a typical version of ACT compared to a version called integrated ACT which offered a team with a substance abuse specialist to provide counselling, treatment groups and outreach and a team trained in integrated treatment principles. At the start of the study, 191 participants were randomised between the three groups. They follow‐up with the study participants at 30 months after the start of the study on a number of outcomes, including two five‐point scales assessing alcohol and drug use abuse severity. The study was rated as low confidence given the high rates of attrition by the end of the study.

The integrated ACT arm had an effect size of 0.23 SD (95% CI, −0.16, 0.63) on the substance abuse rating scale while the ACT had an effect size of 0.12 SD (95% CI, −0.28, 0.52), where a positive number indicates worse substance use severity. However, both effect sizes have large CIs and were not statistically significant and therefore the authors report that the programmes had no group effects on substance use.

Nyamathi (2016) undertook an RCT to evaluate the impact of two interventions compared to usual care, an intensive peer coaching programme and the intensive peer coaching programme combined with intensive nurse case management. Although not described as such by the authors, the intervention appears to be ICM and has been treated as such for this review. The nurse was trained in culturally‐competent case management and spent about 20 min each week with participants on topics including completion of drug treatment, as well as health promotion such as vaccination compliance, and reduction of risky drug and sexual behaviours. The usual care condition involved some limited peer coaching on health promotion and brief nurse counselling, primarily on vaccine uptake. The study took place in California in the USA and worked with 600 male parolees experiencing homelessness. For both treatment arms, the authors were primarily interested in justice related outcomes such as rearrest and reincarceration but also evaluated the impact of the programmes on use of marijuana, stimulants or heroin over the 12‐month observation period.

The authors report that no significant differences were detected on any of the reported drug use outcomes among the two programme groups compared to usual care. For the intensive case management plus peer coaching group, we find odds ratios of 1.16 (95% CI, 0.76, 1.76) for effect on marijuana use, 0.83 (95% CI, 0.54, 1.26) on stimulants use and 0.52 (0.25, 1.09) on heroin use, where an odds ratio of less than 1 indicates a reduction in substance use. For the intensive peer coaching group, we find odds ratios of 1.10 (95% CI, 0.72, 1.66) for the impact on marijuana use, 0.79 (95% CI, 0.52, 1.19) on stimulants use and 0.96 (95% CI, 0.51, 1.78) on heroin use.

O'Connell (2012) is a follow‐up paper to the study originally reported in Rosenheck (2003). The RCT evaluated the U.S. Department of Housing and Urban Development–Veterans Affairs Supported Housing (HUD‐VASH) programme, which offers ICM plus housing subsidy vouchers for veterans with substance abuse issues experiencing homelessness in the USA. Two different versions of the treatments were compared against the TAU offer, ICM plus rent subsidy vouchers and ICM only. At the start of the study, 460 individuals were randomised into the three different groups. This follow‐up study explored how the impacts of these two interventions on the Addiction Severity Index (ASI) for alcohol and drugs varied by sub‐group of veterans, including African American compared to White veterans. The study was rated as low confidence due to high rates of attrition. Comparing ICM and TAU, the authors found an standardised effect size of 0.58 (Hedge's *g*) (95% CI, 0.23, 0.93) in relation to alcohol and 0.12 (Hedge's *g*) (95% CI, −0.23, 0.46) in relation to drugs.

The authors found a significant interaction between race and treatment condition for drug use, specifically that ICM plus rent subsidy vouchers had a larger impact on reducing drug use among African American veterans compared to White veterans. They did not find an interaction between race and treatment condition for severity of alcohol use.

#### 5.4.10 CM

CM is an approach to treatment that maintains that the form or frequency of behaviour can be altered through a planned and organised system of positive and negative consequences. This could include, for example, regular testing and requirements for treatment engagement. CM is an abstinence‐based intervention.

We looked at the subset of studies in our systematic review that assessed the impact of a CM intervention compared to a control group, which is 9 papers reporting on 6 studies. We were able to combine 20 effect sizes from 6 studies reported in 8 papers, using a CHE meta‐analysis with cluster robust estimation. All of these studies took place in the USA. None of the included studies followed‐up over a period of longer than 12 months since the intervention started, with follow‐up ranging between 2 months and 12 months. We do not include Schumacher (2000) in the analysis as they only looked at service use, rather than substance use. However, we present the findings below narratively.

Although we categorised 6 studies as CM, they still evaluated a diverse range of CM interventions that worked with different populations. Koffarnus (2011) randomly assigned adults experiencing homelessness and alcohol‐dependency to 3 groups: either a group requiring abstinence from alcohol to engage in paid job skills training, a group that offered paid job skills training with no abstinence contingencies or a group offering unpaid job skill training with no abstinence contingencies[1]. Kashner (2002) is an evaluation of a US federal compensated work therapy programme that provides financial rewards to participants, homeless, substance‐dependent veterans, who demonstrate continued abstinence. Reback (2010) evaluate a community‐based HIV prevention programme working with homeless men that targeted reduced substance use and increased health‐promoting behaviours. Participants in the CM condition earned points for drug/alcohol abstinence as well as for undertaking health‐promoting behaviours. Milby (1996) combined group‐orientated, abstinence‐based day treatment with CM. After 2 months of the day treatment, participants went on to participate in 4‐months of work therapy which was contingent on drug‐free urine toxicologies. Milby (2000) (also reported in Milby, 2003; Schumacher, 2000, 2003) also test the impact of abstinence‐based day treatment combined with abstinence contingent housing and work therapy. Finally, Smith (1998) combine the provision of the drug Disulfiram and a community‐reinforcement approach with abstinent‐contingent housing.

**Figure 12:**
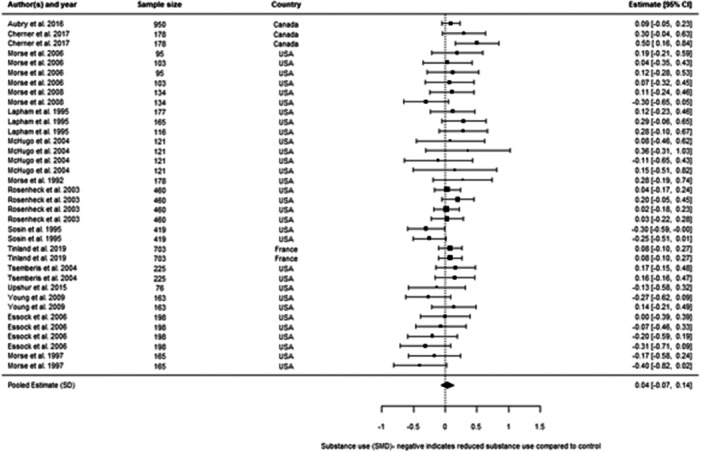
Average effect of contingency management programmes on substance use by adults experiencing homelessness.

We calculated an average effect of –0.47 SD (95% CI, [−0.72, −0.21]), indicating on average a large reduction in substance use for people experiencing homelessness that participate in the included CM programmes compared to a control group not receiving CM. There is some heterogeneity across studies, which can be observed by looking at the forest plot. We calculated prediction intervals, that is, the expected range of true effects in similar studies in future settings (IntHout et al., 2016). We estimated these to be –0.47 SD (−1.09, 0.16), which contain a much larger range of treatment effects than the CIs, although most of the interval falls on the side of the line of no effect that indicates a reduction in substance use as a result of being involved in these types of programmes. We calculated an *I* ^2^ value of 50%, with 12% due to between‐study heterogeneity and 38% from within‐study heterogeneity (and the remaining 50% from sampling variation) (Figure 12).

#### 5.4.11 Sensitivity analysis – CM

We intended to do the following types of sensitivity analysis:

(1) Different values of rho (the correlation among groups of effect sizes)
(2) Removal of low confidence studies
(3) Removal of quasi‐experimental studies
(4) Removal of studies where an effect size had been converted from a binary to a continuous outcome

As we found with the previous analysis, we found that the point estimates and CIs were not sensitive to a range of values of rho (from a very low value of rho of 0.05 up to a very high correlation of 0.80). All of the included studies in the contingency management meta‐analysis were RCTs, and therefore we did not need to undertake sensitivity analysis along this dimension.

The results were sensitive however to inclusion of low confidence studies and inclusion of effect sizes converted from a binary to a continuous outcome:

- After removing the studies rated as low confidence (Koffarnus, 2011; Milby, 1996; Schumacher, 2003), the average effect was −0.54 SD (95% CI −0.94, −0.14) – 4 clusters, 9 effect sizes.
- After removing the studies with an effect size converted from a binary to a continuous outcome, the average effect was −0.66 SD (95% CI, −1.29, −0.05) – 3 studies, 9 effect sizes.

#### 5.4.12 Narrative synthesis – CM

Schumacher (2000) reports on the impact of an abstinence‐based day treatment combined with abstinence contingent housing and work therapy on the number of scheduled sessions of treatment attended (also reported in Milby, 1996, 2000; Schumacher, 2003). People in the treatment group had access to rent‐free housing, if they provided four consecutive drug‐free urine test results during the first 2 months of treatment. Alongside this, they had access to abstinence‐based day treatment. During the next 4 months, they were offered housing for a modest price, again contingent on abstinence from drugs and were also eligible for supervised, paid work therapy. The control group offer was for the abstinent day treatment only. The participants were crack‐cocaine dependent homeless people. The average age of participants was 37.7 years and 72% of the people involved in the study were male. Eighty‐three percent of the sample were African American. The sample size at the beginning of the study was 141 people, with 72 people in the intervention group and 69 in the control group.

The authors found that after 2 months, the intervention group receiving abstinent contingent housing attended a large number of scheduled sessions that the control group, with a Hedge's *g* of 0.92 SD (95% CI, 0.57, 1.26). Although not a focus of this systematic review, the authors found that abstinence was a function of treatment attendance, that is, the more sessions attended, the higher the likelihood of abstinence.

#### 5.4.13 Harm reduction psychotherapy

Harm reduction psychotherapy draws on the concept of harm reduction within a psychotherapeutic approach that integrates cognitive and behavioural interventions with a psychodynamic understanding of substance use as personally meaningful. This approach emerged in the 1990s, challenging the idea that problematic substance use is a disease (Milet, 2021), and with an assumption that individuals might use substances to alleviate psychological, social, and economic distress (Heather, 2018). This intervention assumes that individuals have autonomy, and is non‐judgemental in approach. As its name implies, it is a harm reduction based intervention.

To date, the HaRT‐A programme (Harm Reduction Treatment for Alcohol) is the only harm reduction psychotherapy intervention to be tested in a RCT (Milet, 2021). Developed in Seattle, Washington, using a co‐production approach that involved people experiencing homelessness and problematic use of alcohol – HaRT‐A involves four treatment sessions, using motivational interviewing and therapy, with participants developing their own harm reduction goals (Collins, 2019).

Two papers about this intervention are included in this systematic review, and are synthesised here. Both studies report a reduction in use compared to treatment as usual.

Collins (2019) is the first of the papers, about the effectiveness of this intervention. A total of *n* = 169 participants were recruited between October 2015 and February 2017, of whom *n* = 125 were included at analysis (*n* = 65 receiving the intervention, *n* = 60 not receiving the intervention). The average age of participants was 47.86 years (SD = 9.56), and participants were overwhelmingly male (*n* = 40, 24%, were women). Fifty eight percent of the sample identified as Black/African American, with White/European American representing 22% of the sample. This study examined a number of different outcomes of interest, including two around alcohol use. ‘Peak alcohol use’ was a self‐reported measure of the number of drinks consumed on the heaviest drinking day in the previous 2 weeks. The second measure, and the one included in this systematic review, was self‐reported number of days intoxicated in the previous 2 weeks. This is a measure of frequency from the 5th edition of the Addiction Severity Index (Mclellan et al., 1992), which was turned into a dichotomous rating of the number of days intoxicated.

The researchers reported a difference in means between the treatment and control group at post test and 3 months, with a small difference at 1 month follow up. The researchers report that the treatment effect for this measure was significant, but that time × treatment interaction effect was not significant. We calculated the odds ratio to be 1.77 (95% CI between 0.87 and 3.61), and the Cohen's *d* effect size as 0.31 (i.e., participants in the treatment group were more likely than those in the control group to see a reduction in their alcohol consumption).

The second paper on HaRT‐A was published in 2021 (Collins, 2021). This examined a variation of HaRT‐A, which involves combining it with a pharmacological treatment (naltrexone). Naltrexone is a medication that is used to manage alcohol or opioid disorder by reducing the cravings or feelings of euphoria associated with their use. The study compared this intervention with HaRT‐A + a placebo, HaRT‐A only, and treatment as usual. Between October 2013 and November 2017, *n* = 308 individuals were recruited and randomly assigned to one of the treatment groups, of whom *n* = 190 were included in the analysis. This included *n* = 48 in the HaRT‐A + naltrexone group (*n* = 74 at baseline), *n* = 48 in the HaRT‐A + placebo group (*n* = 78 at baseline), *n* = 54 in the HaRT‐A only group (*n* = 79 at baseline), and *n* = 40 in the TAU control group (*n* = 77 at baseline). The treatment lasted 12 weeks, with follow ups until 36 weeks.

For the *n* = 309 individuals at the start of the trial, the average age ranged between 46.55 years (SD = 10.46) for the HaRT‐A + placebo group, through to 49.27 (SD = 9.11) for the HaRT‐A + naltrexone group. All four groups were mainly men (79% in the TAU group to 87% for the HaRT + placebo group). Black/African America and White/European America were the two largest ethnic groups in each of the treatment groups.

The study examined a number of outcomes. Primary outcomes used self‐reported measures, including peak alcohol quantity, alcohol frequency, alcohol related harm, and measures of physical and mental ill health. Secondary outcomes included treatment adherence, and objective measure of alcohol frequency via blood test. It is this latter measure that we include in this systematic review, measured at two points: 3 months and 9 months. We use the author calculated odds ratio for both of these, as well as our own SMD, for the alcohol frequency via blood test outcome, where an odds ratio of more than 1 and a positive Hedge's *g* indicate a reduction in substance use.

For the main treatment group of interest, HaRT‐A + naltrexone, at 3 months the odds ratio were 2.77 (95% CI, 1.01, 7.58) (Hedge's *g* = 0.56 SD) and at 9 months, odds ratio of 1.60 (95% CI, 0.57 to 4.49) (Hedge's *g* = 0.26 SD). In relation to HaRT‐A + placebo, the effects were 0.91 at 3 months (95% CI, 0.28 to 2.95) (Hedge's *g* = −0.05 SD) and 1.09 (95% CI, 0.36 to 3.33) (Hedge's *g* = 0.05 SD) at 9 months respectively. For the final treatment group of interest, HaRT‐A only, at 3 months the odds ratio was 4.02 (95% CI, 1.46 to 11.11) and the Hedge's effect size at 0.76 SD. At 9 months, the odds ratio was 1.16 (95% CI, 0.40 to 3.34) (Hedge's *g* = 0.08 SD). While these appear to show that those in the treatment groups were more likely than individuals in the control group to see a reduction in their alcohol consumption at 9 months compared to baseline, the difference was not statistically significant.

The researchers report that during the 12‐week period of treatment, the three treatment groups did better than the control, but that after treatment ceased, the three HaRT‐A groups saw outcomes plateau, whereas the treatment as usual group continued to see improvements.

#### 5.4.14 Motivational interviewing

Motivational interviewing is designed to strengthen the personal motivation of the participant, to develop and commit to a specific goal by exploring the individual's own reasons for change. It is non‐judgemental and collaborative in approach, and usually adopts a harm reduction approach. In the analysis presented in this review, we have assumed primary studies of the effectiveness of motivational interviewing interventions are harm reduction‐based, unless otherwise stated in the primary research. Motivational interviewing is used as both a stand‐alone intervention, but also as part of multi‐component interventions, where it is combined with other approaches. (This is the case for HaRT‐A, a multi‐component intervention, i.e., primarily described as harm reduction psychotherapy, but includes motivational interviewing.) It can be done one‐to‐one or in group settings. We identified two studies that assessed the impact of a motivational interviewing intervention.

One of the included studies examines the effectiveness of motivational interviewing in addressing problematic substance use. The AWARE programme is a group motivational intervention aimed at young people aged 18 to 25 experiencing homelessness in Los Angeles County, California, with the aim of reducing alcohol consumption and risky sexual behaviours (Tucker, 2017). The intervention involves four, 45‐minute‐long group sessions, delivered through two drop‐in centres in Hollywood and Venice in Los Angeles.

A sample of *n* = 200 participants were enroled in this RCT, equally split between the treatment and control group (treatment as usual, able to access all services at the drop‐in centre). The sample was mostly men, with just over a quarter (27%) being women. The largest ethnic group was white (31%), followed by African American (24%) and Latino (21%). The mean age was 21.81 (SD = 1.87), with an average age of 16.01 (SD = 3.23 years) when they first left home. The authors state (p5) that participants' most recent period of homelessness had lasted 20.62 months (SD = 29.99 months) (NB as reported by the authors). The researchers report that there were no significant differences between the treatment and control group.

The evaluation used a number of outcome measures, including alcohol and drug use, and sexual behaviours, all of which were measured at baseline and 3 months. We extracted data for three frequency of consumption measures for alcohol, marijuana, and other drugs. Each of these measures was self‐reported. In relation to frequency of alcohol consumption, the authors report standard deviation of means (post intervention) at 2.33 for the intervention group compared to 2.03 for the control group (pooled SD at 2.204). For marijuana consumption, 2.98 for the intervention group compared to 3.02 for the control group (pooled SD at 3.00); and for other drugs 0.59 compared to 0.94 (pooled SD at 0.785). The authors stated that there were positive changes in alcohol consumption (i.e., reduced alcohol consumption), but that there were no significant treatment effects in terms of the two drug consumption outcomes.

The second study by Thompson (2020) evaluated the impact of a brief motivational interviewing intervention combined with the provision of a smartphone application called OnTrack for participants to self‐monitor their substance use and sexual risk behaviours. The intervention was targeted at 18‐ to 21‐year‐olds who were experiencing homelessness. The participants in the intervention group were compared to a TAU comparison group, who were offered usual substance use referral or services at the shelter where the study took place, as well as HIV testing. At the beginning of the study, 60 young people were randomised into the intervention and control group and followed up after 2 weeks, 4 weeks and 6 weeks for the number of times they had drank alcohol each day in the past 2 weeks and how many times per day in the past 2 weeks they smoked marijuana.

The authors found that young people in the OnTrack and motivational interviewing group had significantly lower odds than those receiving TAU services for drinking alcohol (OR of 0.14, 95% CI [0.03, 0.64]). They found an odds ratio of 0.39 (95% CI, 0.065, 2.33) for smoking marijuana.

#### 5.4.15 Residential rehabilitation

Residential rehabilitation is a drugs and alcohol treatment programme delivered in a communal residential, non‐hospital setting, where ‘live in’ treatment is provided. These programmes may include, but usually follow, detoxification. They differ in terms of length of stay, and are usually abstinence‐based. Treatment programmes can draw on different approaches, but usually involve a structured programme including individual and group therapy. Programmes may employ therapeutic communities, 12 step, or cognitive behavioural therapy approaches.

Four of the included studies evaluated residential rehabilitation programmes, three of which were published in the 1990s and one was published in 2011. Two of the studies focused on veterans experiencing homelessness, of which one was about women. Overall, these studies suggest no real difference between residential rehabilitation and treatment as usual in terms of substance use outcomes.

Burnam (1995) evaluated two different interventions compared to treatment as usual for people experiencing homelessness, problematic substance abuse, and mental ill health. One of the treatment interventions was a residential rehabilitation programme, based on a social model recovery approach. This model focuses on peer to peer interactions and individual recovery plans, and is often seen as an alternative to clinically‐orientated approaches. 12 step programmes such as Alcoholics Anonymous are also based on the social model recovery approach. The second treatment intervention used a similar programme, but without a residential component. The evaluation compared these two treatments with treatment as usual, which the authors describe as access to other available community services, such as shelters, clinics, and 12 step programmes. Both treatment interventions involved 3 months of intense treatment (residential or non‐residential), followed by a 3 month non‐residential programme of activities.

Two hundred and seventy six (*n* = 276) met the study's eligibility criteria and were randomly assigned to the residential rehabilitation programme (*n* = 67), non‐residential programme (*n* = 144), or the treatment as usual group (*n* = 65). The average age of the sample was 37 years (no standard deviation provided), and mostly men (84% in total, no standard deviation provided). Fifty eight percent are reported as being White, and 28% Black. Participants had been homeless for just under 5 years on average, and had spent 49 of the previous 60 days experiencing street homelessness.

The researchers measured a number of outcomes, including frequency and severity of drug and alcohol use, a number of symptoms of mental ill health, and several housing stability measures. We were unable to calculate effect sizes from the published data, as the authors did not provide standard deviations or standard errors. The authors report findings from two sets of analysis: treatment versus control (where data from the residential and non‐residential programmes were combined for analysis), and residential versus non‐residential treatments.

The authors report that there were no significant differences between treatment and control groups in terms of substance use outcomes, although they do report that both groups saw improvements in this area. The authors also report no significant differences between the residential and non‐residential treatment groups in terms of substance use outcomes. The paper makes clear that a significant proportion of those assigned to each treatment group failed to attend either programme, with 25% of participants completing the residential programme and 8% completing the non‐residential programme. The research team noted significant exposure effects at 3 months, concluding that longer exposure may results in greater improvements. However, they also noted that this exposure effect was not seen at six or 9 month follow ups.

Kendon Conrad and colleagues (Conrad, 1998) evaluated a 6‐month duration residential rehabilitation programme which included follow up and case management services. Overall, the intervention was intended to last for a year. The intervention was targeted at veterans experiencing homelessness and problematic substance use. The intervention was based in a 30 bed unit, located on hospital grounds. Those participating in the programme undertook inpatient detoxification at the hospital, followed by 3 to 6 months in the residential rehabilitation unit, followed by a period of follow up and case management. The evaluation lasted for 5 years, with a sample of *n* = 358 participants (*n* = 178 in the treatment, and *n* = 180 in the control group). The control group was treatment as usual, which involved inpatient detoxification followed by referral to half‐way house or community based non‐residential support services. All of the sample were men, as only men were eligible for inclusion in the study. The average age of the sample was 40 years, ranging from 25 to 70 years (standard deviation not provided). Most of the sample were African Americans (75%). All of the sample experienced problematic substance use, with two thirds had problems with two or more substances.

The research team utilised a number of outcome measures, including several around frequency and severity of use of alcohol and drugs. We have calculated the odds ratios for the reported composite of six alcohol measures at 0.43 (95% CI between *x* and *y*) (Cohen's *d* = −0.476), and the odds ratio for the reported composite of six drug measures at 0.74 (95% CI between *x* and *y*) (Cohen's *d* = −0.169). The researchers reported that both the treatment and control groups improved over the intervention duration, with the treatment group doing better early on, but the effects decreasing over time. In terms of drug use, again both groups saw improvements compared to baseline, with statistically significant differences between the treatment and control groups at 3, 6 and 9 months. As with the alcohol measure, these effects diminishing over time. The research team stated that they expected the 3 month differences between treatment and control would be greatest at 3 months, because the treatment group would still be in the residential accommodation at this point. They also concluded that the small effects at 12 months were puzzling.

The final residential rehabilitation study from the 1990s included in this systematic review is by Gerald Stahler and colleagues (1995), which compares a residential rehabilitation intervention, a shelter‐based intensive case management intervention, and treatment as usual, based in Philadelphia, Pennsylvania. Both the case management and treatment as usual groups were shelter based. The study lasted for 18 months, and recruited *n* = 722 participants, randomly assigned to the residential rehabilitation treatment (*n* = 222), intensive case management (*n* = 200), and control group (*n* = 302). The study sample only included men. The average age was 32.6 years, 23% were veterans, and 92% are recorded as being Black. On average, the individuals in the sample spent 52 of the previous 60 days experiencing homelessness.

The researchers examined a number of outcomes, including housing stability, drug and alcohol use, employment, and what are described as ‘psychological troubles’. Of interest here are outcome measures around alcohol use and cocaine use, and the study also reported days abstinent over previous 30 days from alcohol and cocaine.

We were unable to calculate effect sizes from the published data, as the authors did not provide means or standard deviations. The research team report stated their assumption that the residential rehabilitation treatment would be more effective than the intensive case management intervention, and both would fare better than the treatment as usual. They report that there were improvements across all outcomes for all three groups, but no significant differences between the two treatment groups, or between the treatment groups and the control group. The authors state that those in the two treatment groups reported higher levels of satisfaction about the services they received.

More recently, in 2011, Ilan Harpaz‐Rotem and colleagues (2011) examined the effectiveness of residential treatment services for women veterans. This study focused on eleven Homeless Women Veteran Programmes, which were established in Veteran Affairs Medical Centres. The research team compared participants recruited between 2000 and 2005 who received at least 30 days of residential treatment with those who did not (i.e., the comparison group included women who had received less than 30 days residential treatment and no residential treatment at all). Women were not randomly assigned to the treatment group in this study. Outcomes were measured at baseline and at 1 year. Outcomes included employment, housing status, and health status. Health outcomes included alcohol and drug composite scales from the Addiction Severity Index, using self‐reported measures of use and days of use over the past 30 days. The authors acknowledge that drug and alcohol use was not objectively observed, and go on to state that this ‘may be particularly problematic because in most cases the client's clinician was the person conducting the research interview’ (p. 897).

The study participants included 217 women in residential treatment, and 234 women in the comparison group. At 12 months follow up, 96 women remained in the treatment group and 119 in the comparator. The average age of the treatment group at baseline was 43.5 (SD 8.9 years) compared to 43.9 years (SD 6.7 years). The authors report the lifetime homelessness of both treatment and comparator groups at 28 months (SD 44 months) and 26 months (SD 31.7 months) respectively. Literal homelessness was also measured, with 16.1% of the treatment group and 22.4% of the comparator group being literally homeless at baseline.

The authors report that on the days of alcohol use measure, the treatment group had higher use on average, and both treatment and comparator groups had significantly decreasing days of use over time. The difference in change of use between the two groups was not significant (*p* = 0.03), controlling for baseline. The treatment group also had significantly higher scores on average for the ASI Drug scale (*p* < 0.001), controlling for baseline, particularly at 3 months (*p* = 0.01) and 6 months (*p* = 0.001). It is also reported that groups also showed significantly decreasing days of drug use over time, but the treatment group did not have a significantly different change in use (*p* = 0.07), controlling for baseline.

The authors make clear that there were differences between the 11 different centres in terms of clinical services and case management approaches. They commented that the differences ranged from ‘from very small professional staffs with primarily peer‐led groups to large professional staffs, and from Alcoholics Anonymous group models to intensive cognitive‐behavioral treatment approaches’. The authors speculate that the provision of stable accommodation during an significant point of transition might be the important ingredient that makes the difference to outcomes, and suggest that more research is needed.

#### 5.4.16 Talking therapies (including cognitive behaviour therapy)

Talking therapies involved the individual or group talking with a trained professional about their thoughts, feelings, or behaviour. Talking therapies might include or be described as counselling, psychological therapies, psychosocial therapies, and including interventions such as cognitive behavioural therapy. (A number of specific therapy interventions, including harm reduction psychotherapy, motivational interviewing, and therapeutic communities, are dealt with separately.) These interventions typically adopt a harm reduction approach.

Two included studies examine the effectiveness of talking therapies, both as part of multi‐component interventions. One considers an intervention that includes dialectic behavioural therapy, and one that examines an intervention called MISSION‐Vet. These findings from these two studies are mixed overall. The first study says that it was effective for drugs only or alcohol only, but not for drugs and alcohol. The second study suggests the intervention was not effective. Taken together, these studies provide no clear insight into whether the interventions are effective.

Dialectic behavioural therapy is based on cognitive behavioural therapy, adapted for individuals who feel emotions intensely. Its main goals are to teach people how to live in the moment, develop healthy ways to cope with stress, regulate their emotions, and improve their relationships with others. An intervention that combined dialectic behavioural therapy and case management, targeting women who were on probation/parole and experiencing homelessness, was evaluated between February 2015 and November 2016 across four sites in California by Adeline Nyamathi and colleagues (Nyamathi, 2017).

This RCT compared this intervention, which was delivered over 6 weekly group‐based sessions and 6 weekly one to one sessions, with a health promotion intervention. The evaluation involved *n* = 130 women aged between 18 and 65 years, with a history of problematic drug use and who were homeless when released from prison. Participants were equally split between the intervention and comparator, with an average age in the treatment group of 38.6 years (SD 11.3) and 39.1 years in the control group (SD 11.5). The authors report that the two largest ethnic groups in both the treatment and control were Black (44.6% and 36.9% respectively) and Latino (40% in each group). They also report that 10.8% of the treatment group and 16.8% of the control group were White. The primary outcome of interest was drug abstinence, with alcohol abstinence also being of interest. Outcomes were measured using self‐reported measures, but also objectively with a urine test.

The authors report that there were improvements in drug abstinence at the 6‐month follow up for both treatment group and control group participants, with a greater increase seen in the treatment group. They also report that participants in the treatment group were more likely to become or remain abstinent from alcohol than those in the control group. In discussing drug and alcohol abstinence, the authors state that ‘the differences in increased odds of substance abstinence (abstinent for both drugs and alcohol) were not significant (i.e., the interaction term was nonsignificant; OR = 2.39, 95% CI [0.92, 6.23], *p* = 0.07)’ (p. 437).

The second paper included in this systematic review that examines talking therapies is by a team of researchers led by David Smelson (2018), which evaluated an intervention called Maintaining Independence and Sobriety Through Systems Integration, Outreach and Networking – Veterans Edition, known as MISSION‐Vet. This intervention was an adapted form of the HUD‐VASH programmes which provides subsidised housing and case management service. The adaptation evaluated in this study included ‘certain enhancements like integrated dual disorders treatment, peer support, supported employment and trauma informed care’ (p. 2) and includes a Critical Time Intervention and Dual Recovery Therapy (p. 3). The study focused on a number of outcomes, including treatment engagement, service utilisation, housing, and drug and alcohol use. It is these last two that are relevant to this systematic review.

A total of *n* = 168 individuals participated in the evaluation, of which *n* = 81 were in the treatment group. The paper provides some demographic information about participants, stating that treatment group participants were significantly older, had been in housing longer, were less likely to need treatment for a medical problem than those in the control group. No information on age, sex, or ethnicity of participants is provided.

The researchers provide results of mixed effects models for drug and alcohol use, with adjusted odds ratios. They do not provide CIs, standard errors, or p‐values. The authors report treatment versus control for changes in alcohol and drug use, stating that ‘neither the main effect of membership in the MISSION GTO group, nor the interaction with time were statistically significant predictors in the other outcome models’ (p. 5). This suggests that the intervention was not effective. The authors provide odds ratios of 0.84 (*p* = 0.19) for drug use, and 0.83 (*p* = 0.11) for alcohol use, for treatment × time versus control.

#### 5.4.17 Therapeutic communities

Therapeutic communities are structured, participatory, group‐based, residential interventions for long term mental ill health, personality disorders, and problematic substance use. Treatment focuses on drug abstinence, coupled with social and psychological change that requires a multidimensional effort involving intensive mutual self‐help typically in a residential setting. Therapeutic communities are often a component in an overall intervention, and there is a body of research evidence around these types of interventions.

None of the included studies used the term therapeutic communities to describe the intervention of interest. Several papers cover interventions that include components or approaches that are consistent with the definition of therapeutic communities, but are not identified as such. In most of these cases, the intervention is multi‐component, where the primary component is one that is included in our typology of interventions, and these are included elsewhere in this systematic review.

One paper, by Gerald Stahler and colleagues (2005), evaluates an intervention that appears to be a therapeutic community. The paper covers an intervention called ‘Bridges to the Community’. This programme was developed to work within a residential rehabilitation service, and involved peer mentors called community anchor persons, and group activities including workshops, training sessions, and cultural and recreational activities. The programme was targeted at African American women with children at risk of homelessness, lasted between 6 and 9 months in a residential programme called Hutchinson Place in Philadelphia, Pennsylvania. The programme was compared with standard treatment, which involve residential rehabilitation described by the researchers as being ‘largely 12‐step orientated’ (p. 174).

The authors make clear that this was a preliminary demonstration project, and that participants were not randomly assigned to either the treatment or control group. *N* = 47 women participated in the treatment group, with an average age of 34.4 years (SD 4.8 years), of whom 95.8% were African American. The researchers report that the treatment group were older than the control group, but that this was not considered to be material. The treatment group reported lower drug and alcohol use in the previous 30 days, which the researchers took into account in their analysis.

The paper includes three outcome measures of interest: (1) average days use of cocaine in the past 30 days; (2) average days use of alcohol in the past 30 days; (3) average days use of more than one substance in the past 30 days. The researchers report the means and standard deviations for the treatment and control group, as well as the F‐value, p‐value, and effect size. The authors state that ‘at follow‐up, 100% of participants in the Bridges group reported that they had abstained from cocaine in the last 30 days compared to the standard treatment group, which reported an average of about 2 days of use’ (p. 176) and that both groups saw reductions in use. Women in the treatment group were also more likely to remain in treatment.

#### 5.4.18 Group work

Group work in the context of substance use treatment can involve a variety of group treatment models to meet client needs during the multiphase process of recovery. This may include skills development groups, cognitive–behavioural/problem solving groups, or support groups.

One of the included studies examined group work as one element of an overall intervention called LEAP (Life Enhancing Alcohol‐management Programme) and published by Seema Clifasefi and Susan Collins (Clifasefi, 2020). The paper includes details on how the intervention was developed through a coproduction approach called community‐based participatory research (CBPR), as well as details on the intervention and how it was tested. All participants were Housing First residents, who were non‐randomly assigned to the treatment group and control group. A total of *n* = 116 individuals participated in the research, of whom *n* = 66 were in the treatment group (a single‐site Housing First programme that offered the intervention) and *n* = 50 in the control (across two single‐site Housing First programmes that did not offer the intervention). All three Housing First sites were in Seattle, Washington.

The authors report that there were not significant differences between the treatment and control groups for alcohol quantity or alcohol‐related harm (*p*'s > 0.06); but there was a significant difference in terms of non‐drinking days (Wald *χ* ^2^(4, *N* = 105) = 23.01; *p* < 0.001) The authors conclude that ‘there was a significant main effect for the intervention group, which indicated lower odds of nondrinking days (OR = 0.25; robust SE = 0.11; *p* = 0.002) at baseline for LEAP versus control participants. The time × group interaction, however, was not significant (*p* = 0.71)’ (p. 769).

#### 5.4.19 Research Question 6: How do participant and study characteristics moderate the effect of interventions designed to reduce substance use in adults who are experiencing homelessness?

##### How does the length of follow up period moderate effectiveness?

As reported in the descriptive findings above, different studies followed‐up over different time periods and many studies reported multiple follow‐up outcome data collection points for the same individuals. Fifteen of the effect sizes in the data set used to answer Research Question 1 were for a short‐term follow‐up (up to 6 months), while 30 were for a long term follow‐up point (from 6 months up to 36 months). When we explored the influence of time on substance use outcomes, by including a variable in the meta‐analysis indicating whether the effect size was for a short‐term follow‐up point or a long‐term follow‐up point, we found a very small difference on average effect size that was not statistically significant (a *p*‐value of 0.74). The average effect size for short term follow‐ups was −0.14 SD (95% CI, −0.38, 0.10) and the average effect size for long term follow‐ups was –0.10 SD (95% CI, −0.27, 0.06).

We did not have a sufficient number of studies or effect sizes to explore variation in outcomes using meta‐regression by:

- Groups of participants with specific needs beyond their experience of homeless and problematic substance use.
- Interventions that targeted different substances.

[1] We only include the impact results for the group requiring abstinence from alcohol in our meta‐analysis below, in comparison to the other two groups.

[1] https://ies.ed.gov/ncee/wwc/docs/referenceresources/wwc_brief_attrition_080715.pdf

## 6 DISCUSSION

### 6.1 Summary of main results

This systematic review aimed to understand the effectiveness of substance use interventions in reducing, stopping, or preventing problematic substance use by adults experiencing homelessness, and to provide insight for policy makers, commissioners, and service providers on which interventions are more effective. We included 48 papers, from 34 unique studies. Of these, 42 of the included papers reported on RCTs. Many of the studies were assessed as presenting a high risk of bias. High attrition rates partially explain this high risk of bias, and should be expected given the population of interest. But several studies also present higher risk of bias due to use of quasi‐experimental methods that may not be able to address selection bias and confounding.

The 48 included papers covered 15,255 participants. They covered a multitude of outcomes above those of interest to this review, including service usage, housing stability, general health, mental health, risky behaviours, and crime. Many different measures were used, over several different time frames. The focus of this review was on substance use outcomes. These outcomes are measured using different measurement variables covering both objective measures and self reported measures, in different ways and over various time frames.

The 48 included papers covered 7 primary interventions. Many of these interventions involved multiple components, were variations of previously evaluated interventions, or were linked to a housing intervention. Despite the huge debate in the wider theoretical and practitioner literature around the comparative efficacy of abstinence or harm reduction approaches, few of the included intervention studies explicitly identified the intervention of interest in this way, and fewer provided theoretical justification for their chosen approach. Overall, the findings here suggest that the substance use interventions being evaluated were more effective than treatment as usual in reducing problematic substance use. The effect size is small and there is a chance that these interventions make no discernible difference (the overall effect calculated at –0.11 SD (95% CI, [−0.27, 0.05], 15 studies). This analysis covered a number of different interventions of interest, including ACT and ICM, CM, Residential Rehabilitation, and Motivational Interviewing techniques combined with a range of other interventions. Given this range of interventions, it is not surprising there is a large degree of heterogeneity in the data.

The key policy focus of this review related to the effectiveness of abstinence requirements and harm reduction approaches in substance use interventions for adults experiencing homelessness. Abstinence‐based approaches have dominated service provision historically, but since the 1980s a significant movement towards harm reduction has taken place. In relation to people experiencing homelessness, much of this change has taken place because of the introduction of Housing First as a philosophy for service provision, based on the values of facilitating individual's agency and choice, providing immediate, non‐contingent housing, and separating accommodation for support and treatment services.

This review found that the abstinence‐based interventions tested in the studies included in this review were on average more effective than treatment as usual (RQ2) in terms of stopping or reducing problematic substance use by people experiencing homelessness, with a moderate average effect size of –0.28 SD (95% CI, −0.65, 0.09) (6 studies). Note that these abstinence‐based interventions were all compared against a treatment as usual service offer that was primarily abstinence‐based service.

The review found little difference between the average effect for the harm reduction interventions tested in the studies included in this review compared to treatment as usual (RQ3), with the average effect size close to 0 at 0.03 SD (95% CI, −0.08, 0.14) (9 studies). Note that these harm‐reduction interventions were usually compared against an abstinence‐based treatment as usual offer (7 studies) or control conditions that could not be categorised as either abstinence‐based on harm reduction‐based (2 studies).

These results need to be interpreted with some caution. The estimates are based on a small number of effect sizes and studies and the interventions being tested and populations targeted are also diverse within both the abstinence and harm reduction categories. Most of the harm reduction studies were rated as being of low confidence. Of the seven harm reduction studies included in this part of the analysis, six were assessed as being of low quality (*n* = 6/7) and only one was of high quality. This is in contrast with the abstinence based studies: four of the six studies included in this part of the analysis were assessed as being high or medium quality (*n* = 4/6) and two of low quality (*n* = 2/6).

In addition, the most common intervention tested in the harm reduction category was ACT and ICM (six of the nine included studies). There is therefore an argument that this analysis is for the most part answering the question of whether ACT and ICM are effective compared to treatment as usual, rather than whether harm reduction interventions in general are effective compared to treatment as usual. There are a range of other harm reduction interventions in our framework that have not been tested using impact evaluation methods for people experiencing homelessness, that may have different impacts on substance use outcomes.

Finally, it is worth stressing that the primary objective of abstinence‐based interventions is to stop or reduce substance use, and that abstinence is a requirement of continued engagement in these treatments. In contrast, while all of the included harm reduction studies measured substance use as an outcome of interest, stopping or reducing substance use is not the primary objective of, or requirement for, receipt of these interventions. Given this, it is perhaps not surprising that the statistical analysis found little difference between treatment and control for harm reduction interventions.

We also searched for studies that made a direct comparison between a new harm‐reduction and a new abstinence based approach (RQ4), in order to attempt to answer the question of whether abstinence‐based interventions appear to be more or less effective than harm reduction‐based interventions. However, because of the limited number of studies that made this direct comparison, most of which were restricted to the same type of intervention, we are unable to draw conclusions about whether in general abstinence‐based interventions are more or less effective than harm reduction approaches.

Over and above the issues identified with our analysis of harm reduction versus treatment as usual, abstinence versus treatment as usual, and harm reduction versus abstinence, our analysis suggests that these different approaches make little real difference to the outcomes achieved. Our analysis does suggest, though, that some individual interventions are more effective than others (RQ5). We were able to perform meta‐analysis in relation to two of the individual interventions covered in the 48 included studies, ACT and ICM, and CM (an abstinence based approach that includes a number of different interventions). For those interventions covered by included studies but for which were unable to undertake meta‐analysis, we synthesised narratively. Interventions for which narrative synthesis of the evidence base was taken were: harm reduction psychotherapy, motivational interviewing, therapeutic communities, and residential rehabilitation.

Of the two interventions where we were able to undertake meta‐analysis, CM was found to be effective on average in reducing, stopping, or preventing problematic substance use by adults experiencing homelessness. Contingency management is an abstinence‐based group of interventions, which used positive or negative consequences (typically vouchers or exclusion from housing or services) to alter the form and frequency of an individual's behaviour. We find that these interventions are effective on average: we calculated an average effect of –0.47 SD [95% CI, −0.72, −0.21]), indicating on average a large reduction in substance use for people experiencing homelessness receiving a contingency management intervention compared to treatment as usual or a non‐contingency management intervention. There is some heterogeneity in these data, and it is worth stressing that all of the included studies were from the USA.

One of the ongoing questions about the effectiveness of contingency management interventions is around their long‐term effectiveness. This is because these interventions provide an immediate and tangible reinforcement (either positive or negative) for reduced substance use behaviours. But what happens when the reinforcers are removed? While some recent studies appear to address this question in relation to contingency management interventions in general (i.e., not specific to people experiencing homelessness) (see, e.g., Ginley et al., 2022), the studies included in this review do not allow us to address this issue. None of the included studies followed‐up over a period of longer than 12 months since the intervention started, with follow‐up ranging between 2 months and 12 months.

The second intervention where we were able to conduct meta‐analysis is that of ACT and ICM. Since the 1980s, various case management interventions have been developed, of which ACT and ICM are two. These approaches typically involve outreach, assessment, planning, onward referral to relevant services, and advocacy. ACT and ICM are related interventions, both of which combine case management with direct delivery of specific services, include substance use treatments. These interventions are often combined with permanent supportive housing, or (in the case of the At Home/Chez Soi programme in Canada) with Housing First. (Of the 47 papers included in this review overall, 9 relate to the At Home/Chez Soi programme, although only one of these was included in the meta analysis conducted in relation to ACT/ICM.)

Most of the studies included in the meta‐analysis took place in the USA. Three did not: Tinland (2020) took place in France, and Aubry (2016) and Cherner (2017) took place in Canada. Follow‐up ranged from 6 months up to 36 months.

We calculated an average effect of 0.04 SD (95% CI, [−0.07, 0.14]). This indicates that the average effect of ACT or ICM programmes on substance use is close to 0. As the CIs include values on both sides of the line of no effect, it is possible that substance use might reduce or increase as a result of being involved in ACT or ICM programmes. As there can sometimes be a difference in the assessed needs of individuals accessing these two services, we also included whether the programme is ACT or ICM as a moderating variable. We found that there is no difference in the average effect between the two types of programme.

Assertative Community Treatment and ICM are both described as being evidence‐based interventions in the wider literature. It is therefore worth stressing that our analysis here is focused only on substance use outcomes of these interventions, and not their effectiveness in terms of other interventions such as symptoms of mental ill health, or housing stability. Our findings here are consistent with those in other reviews (see, e.g., de Vet, 2013; Fries, 2011; Penzenstadler, 2019), namely that these interventions have small or no effect on substance use outcomes. We undertook a narrative synthesis of the eight At Home/Chez Soi papers that were not included in the meta analysis presented here. Taken together, these studies show a lack of a significant impact on substance use. While some do include some effect sizes that indicate a small positive or negative impact on substance use for the intervention group compared to control, almost all have wide CIs that span the line of no effect.

### 6.2 Overall completeness and applicability of evidence

The included studies provide a good representation of the types of interventions used to address problematic substance use by adults experiencing homelessness. There is more evidence in relation to some interventions than others, with a dearth of effectiveness evidence for some interventions included in our intervention typology (in particular, several harm reduction interventions are not included in this review, either in meta analyses or in narrative syntheses). The evidence base is focused on more extreme forms of homelessness, usually focused on people experiencing or at risk of street homelessness, those living in shelters or homeless hostels, and veterans.

Men are disproportionately represented in the included studies. Three of the included studies – Upshur (2015), Harpaz‐Rotem (2011), and Nyamathi (2017) – specifically focus on women's outcomes. Five papers did not provide details on the split between men and women. For the remaining studies, the percentage of the study participants reported as being male ranged from 47.6% to 100%, with an average across these group of studies at 77% (SD 12.54). This overrepresentation of men in the included studies is in part because men are more likely to experience the more visible and extreme forms of homelessness, and it is these forms of homelessness that are often the focus of research in the field. In the UK, men account for 80% of those adults experiencing street homelessness or who are sleeping rough (MHCLG, 2021), though most people experiencing homelessness are women, who are often in non‐visible forms of homelessness such as temporary accommodation, bed and breakfast, or concealed housing (such as floors or sofas in friends or relatives' homes). Indeed, women make up 60% of the group of people experiencing homelessness and living in temporary accommodation, which is a much larger group than those experiencing street homelessness or sleeping rough.

The overrepresentation of men in the effectiveness evidence is also because women are also less likely to engage in problematic substance use. Women make up around a third of all adults entering drug treatment services in England in 2020/21 (OHID, 2021), and a fifth of those entering treatment across the European Union (EMCDDA, 2021). We are not the first to comment on this gap – there is a substantive body of literature that is highly critical of the way in which research ignores women's homelessness and the homeless experiences of women (see, e.g., Mayock, 2020; Bretherton, 2017). But as women experience homelessness differently, and often become homeless for different reasons (domestic violence being a significant driver of women's homelessness), to men, and are likely to face different barriers when they try to access interventions aimed at reducing, stopping, or preventing problematic substance use (EMCDDA, 2021), a significant gap in the evidence base is around the effectiveness of substance use interventions for women.

The evidence base is overwhelmingly from the United States. This presents several challenges when interpreting and using the evidence in a country such as the UK, where there are differences in both the socio‐demographic background of people experiencing homelessness, and the context within which substance use interventions might be accessed by them. In particular, the relatively better access to publicly funded healthcare services for people experiencing homelessness in the UK compared to the United States may reduce the difference between intervention of interest and treatment as usual if the intervention were implemented in the UK. (Though people experiencing homelessness in the UK are still less likely to be able to access primary and community health services, and be more dependent on emergency services, compared to the general population.)

Finally, we found that the interventions were generally undertheorised in the included studies, with few explanations of how doing *x* might result in outcome *y*. While a small number of the studies did include details of the expected causal mechanisms through which interventions were expected to work, most relied on referencing previous studies, or did not provide any theoretical justification for the intervention's design. This is particularly significant given that many of the interventions included were multi‐component interventions, or were variations on previously evaluated interventions, and because people experiencing homelessness face a number of challenges over and above their problematic substance use.

### 6.3 Quality of the evidence

The evidence base suffers from a number of important methodological limitations. Although the evidence base is made up of mostly RCTs, many suffer from high rates of attrition, including differential attrition. While this is unsurprising for this population, it can both reduce the sample size of a study, making it more difficult to detect a difference between groups, as well as introduce bias when there is differential attrition between the intervention and comparison groups. In addition, the vast majority of papers did not report that they used masking/blinding of outcome assessors or masking/blinding of the team to the intervention allocation during analysis. This is important where there is an element of judgement on the part of the assessor during the collection of outcome data or in the analysis of data, as they may behave in ways that differentially affect the outcomes in different treatment groups. Few studies reported that they used power calculations to determine their sample size, and many of the studies had small sample sizes. This limits the ability of the studies to detect a difference between groups, particularly if the expected effect size for this sort of intervention is small.

We found a number of cases in our included studies where the authors primarily considered the statistical significance of a result to determine whether there was an effect of the intervention of interest on substance use, that is, whether or not they found a p‐value of less than 0.05 for their effect estimate. This is in contrast to considering the magnitude of the effect on substance use alongside discussion of statistical uncertainty around that effect. Therefore, in some cases we found a moderate positive or negative effect size with CIs crossing zero, despite the original authors reporting no effect on substance use. We suggest that future studies should consider both the practical significance of an effect size and statistical uncertainty around that effect, alongside other factors that influence strength of conclusions such as internal validity and the plausibility of results compared to the existing evidence base.

### 6.4 Potential biases in the review process

There were several limitations to the review process, as well as limitations to our conclusions that stemmed from the underlying evidence base.

This review was based on searches not undertaken by the review team. While the team did undertake hand searches of relevant journals, completed a call for evidence, and unpacked relevant systematic reviews, the primary source of studies subjected to title and abstract and full review was an existing EGM. The searches for assessments of eligibility for inclusion, and risk of bias assessments were undertaken by a team from the Campbell Collaboration for the Centre for Homelessness Impact, to existing and published standards. The authors were unable to undertake full independent critical appraisals and effect size data extraction and calculation by two reviewers. Instead, one member undertook the critical appraisals of studies that had notbeen included in the EGM, as well as the effect size data extraction and the results were checked by another member of the team.

Despite contacting authors for missing data, there were also several included studies that did not contain the information for us to calculate standardised effect sizes and so were not included in our meta‐analysis. In addition, our goal had been to categorise each study by whether they promoted a harm reduction or abstinence‐based approach and what was offered to comparison group participants. Because of a lack of information in the included studies, we were unable to categorise all studies.

The authors were unable to extract data and calculate effect sizes for every substance use measure presented in the included studies and therefore needed to follow the decision rules laid out above in the section Criteria for determination of independent findings. However, we did ensure that for every paper that reported outcome results for both a self‐reported and objective measure of substance use, we extracted an effect estimate for both.

Because of an insufficient number of studies and effect sizes, we were not able to include all the moderating variables in one analysis, and therefore had to undertake single characteristic meta‐regression where possible. Any conclusions drawn from meta‐regression analysis should always be cautious and exploratory given that these relationships are typically observational in nature and based on a small number of effects. With single characteristic meta‐regression, the identified moderators may be related to one another and the analysis can therefore be misleading.

### 6.5 Agreements and disagreements with other studies or reviews

There are two existing systematic reviews relevant to problematic substance use by people experiencing homelessness. Our review complements these two reviews.

Magwood (2020) completed a review of reviews of the effectiveness of specific harm reduction interventions, including two pharmacological harm reduction interventions, safe consumption rooms, and managed alcohol programmes. The authors report that they did not find any studies specific to people experiencing homelessness, and therefore their included studies covered the general population. (Magwood and colleagues make clear, the studies included in their review included a large number of people experiencing homelessness and indeed the underlying evidence in relation to managed alcohol programmes almost completed related to this population.) The Magwood review does not cover any interventions that are included in our review. Given this, its different methodology (review of reviews), and its wider scope in terms of population of interest, our review complements rather than agrees or disagrees with the review by Magwood et al. (2020).

The second relevant review is that by Aliza Moledina and colleagues, published in June 2021 (Moledina, 2021). This review examined a range of different interventions aimed at people experiencing homelessness, including housing, income assistance, case management, mental ill health, and problematic substance use interventions. As such, the scope of the review in terms of interventions of interest is wider than our review. The Moledina review also considered the costs and benefits of these different interventions, which was not a consideration of our review.

Moledina and colleagues focused on a small number of interventions aimed at addressing problematic substance use. Each of these interventions adopts a harm reduction approach. The reviewers found no evidence of the effectiveness of these interventions. Two of the Moledina interventions – supervised consumption rooms and opiod antogist therapy – were included in our typology of interventions. We also found no effective evidence in relation to these interventions, and our review therefore agrees with the findings of the Moledina review in this respect.

The Moledina review also examined the effectiveness evidence in relation to ACT, and ICM, both of which they classed as mental health interventions rather than substance use interventions. (This reflects the historic development of these interventions, both of which have roots in mental health services.) The reviewers considered these two interventions separately, whereas we considered them as a single intervention and also separately. They were also interested in a number of different outcomes, whereas we were focused only on substance use outcomes. Moledina and colleagues did not undertake meta‐analysis of their included studies for these two interventions, but rather a narrative synthesis. Their findings are similar to ours for both ACT and ICM, in that the found little evidence of any significant effect of these interventions on substance use outcomes compared to controls.

## 7 AUTHORS' CONCLUSIONS

### 7.1 Implications for practice

#### 7.1.1 Implications from the review for policy, commissioning, and practice

This systematic review aimed to understand the effectiveness of substance use interventions in reducing, stopping, or preventing problematic substance use by adults experiencing homelessness, and to provide insight for policy makers, commissioners, and service providers on which interventions are more effective. Our review points to the potential benefits of individual interventions designed to reduce substance use. Nevertheless, high rates of attrition in many studies and small average effect size make it difficult to have high confidence in the interventions. The evidence points to the need for commissioners and service providers to be realistic and expect high attrition rates given the often complex and chaotic nature of this population.

#### 7.1.2 Using incentives can be beneficial

Although our analysis of harm reduction versus treatment as usual, abstinence versus treatment as usual, and harm reduction versus abstinence suggests that these different approaches make little real difference to the outcomes achieved, the findings suggest that some individual interventions are more effective than others. The meta‐analysis of CM, an abstinence‐based group of interventions, which used positive or negative consequences (typically vouchers or exclusion from housing or services) to alter the form and frequency of an individual's behaviour, was found to be most effective in reducing, stopping, or preventing problematic substance use by adults experiencing homelessness. On average, our analysis identified a large reduction in substance use for people experiencing homelessness receiving a contingency management intervention compared to treatment as usual or a non‐contingency management intervention.

#### 7.1.3 Case management does not significantly impact on substance use

Our analysis of case management interventions – ACT and ICM – was less positive. These approaches typically involve outreach, assessment, planning, onward referral to relevant services, and advocacy. Despite these interventions gaining popularity in recent years in policies to tackle homelessness such as Housing First and permanent supportive housing, we found that these interventions lacked any significant impact on substance use. Hence, while they are supported by the evidence base in terms of positive housing and mental health outcomes, they appear less successful in reducing substance use.

#### 7.1.4 Evidence based policy gaps

It is vital that policy makers, commissioners, and service providers have access to high quality evidence about which substance use interventions work – and which do not work – for this population. However, our review found that the existing evidence base available for policy makers, commissioners and services to inform service development is lacking in several areas. There is a clear need for a more robust evidence based to be developed beyond North America. This evidence needs to include the often‐lacking voices of people with lived experience of homelessness in the research design and should also focus specific attention on the effectiveness of substance use interventions for women.

We found that almost all of the interventions were generally undertheorised and lacked explanations such as the expected casual mechanisms through which interventions were expected to work to reduce, stop, or prevent problematic substance use by people experiencing homelessness. More consideration should be given by policy makers, commissioners and service providers to developing the theoretical justification for the chosen approach around what works.

The key policy focus of this review related to the effectiveness of abstinence requirements and harm reduction approaches in substance use interventions for adults experiencing homelessness. Yet no studies that met the inclusion criteria involved a comparison between an abstinence‐based treatment with a harm reduction control. A particular evidence gap relates to harm reduction focused interventions. Many of the harm reduction focused interventions lack evaluated outcome measures in adults who are experiencing homelessness. For example, we found no studies that focused on overdose prevention centres.

### 7.2 Implications for research

The review team has identified four key implications for research from this review.

As with any meta‐analyses, the quality and usefulness of findings is dependent on the rigour and breadth of the underlying included studies. We found that many of the studies were of low methodological quality, and the interventions of interest were generally undertheorised. While there are clearly unique challenges posed with undertaken research in this field, it is nevertheless the case that more can be done to improve the methodological rigour of research in this area. It is also difficult to draw conclusions from studies that do not provide insight into what the intervention of interest is, what it involves, how it is expected to work, and how doing *x* is expected to lead to outcome *y*. It makes it difficult for other researchers, policy makers and practitioners, to draw useful knowledge from the underlying research, which can affect their ability to make improvements to services and interventions aimed at reducing, stopping, and preventing problematic substance use and related harms for people experiencing homelessness.

Secondly, there is a clear gap in the geographical spread of the existing evidence base, which is overwhelmingly from the United States. Primary effectiveness research from other countries could bring unique perspectives to the evidence base, and also likely increase the extent to which evidence can be adapted to the specific contexts of other countries. In particular, the lack of effectiveness studies from the UK is significant and needs to be addressed. More research is need in the UK that would allow policy makers and practitioners to focus on interventions that can be demonstrated to work.

There is also a clear gender bias in the underlying research. While it is the case that men are more likely to experience the more visible and extreme forms of homelessness, and are also more likely to enter treatment for problematic substance use, we would still argue that more research is needed about the experiences of, and what interventions are effective for, women experiencing homelessness. Finally, some of the language used in the included studies can be dehumanising. Given the significant barriers that some who are experiencing homelessness can face when accessing public services, it is important that researchers do not add to or reinforce these barriers by using dehumanising language.

## CONTRIBUTIONS OF AUTHORS

Chris O'Leary is the lead reviewer and is responsible for the overall delivery of the review. He is a public policy specialist, with a specific expertise in providing evidence based advice to policy makers, particularly in relation to interventions designed to prevent and reduce homelessness. He sits on the Manchester Homelessness Partnership, a collaboration between statutory and voluntary agencies in Manchester aimed at reducing homelessness, and improving and quality and effectiveness of homelessness services. His published research on homelessness includes empirical research and evidence reviews. His article reviewing the evidence around homelessness and recidivism (O'Leary, 2013) is rated by Altmetrics as in the top 25% of all research for policy impact, and his 2018 research on homelessness from the private rented sector in England was launched in the UK House of Commons by two All Party Parliamentary groups of Members of Parliament.

Rob Ralphs is a substance use specialist with over 20 years' experience in conducting research on substance users, with a particular focus on drug treatment and policy. Since 2016, he has been the principal investigator on seven research projects that have focused on homelessness, substance use, service development and service user engagement. Through this portfolio of research, he has developed unrivalled experience and understanding of contemporary substance use trends and the challenges of engaging people with lived experience of homelessness into treatment. His recent 2020 paper on the motivations of synthetic cannabinoid use amongst people with lived experience of homelessness and the policy and practice responses is one of the most read articles in the leading international journal Addiction, Research and Theory. His current research focuses on the impact of COVID‐19 on access to drugs and emerging drug trends amongst those experiencing homeless in Greater Manchester. He currently sits on a number of advisory roles including: the Advisory Council for the Misuse of Drugs (ACMD) Novel Benzodiazepines and New Psychoactive Substances Monitoring Committee; the Greater Manchester Drug Alert Panel; the Manchester Professional Advisory Group for Drugs and Alcohol; the Manchester Needle and Syringe Provision Steering Group; the Greater Manchester Combined Authority COVID‐19 Homeless Hotel Substance Use Harm Reduction Advisory Group.

Andrew Smith was responsible for the day to day operation of the review until February 2022. Jennifer Stevenson was responsible for the day to day operation from February 2022 and will be responsible for the meta‐analysis. This review will be supported by specialist statistical methods input from Zsolt Kiss alongside research support from senior research assistant Jordan Harrison and Harry Armitage.

## DECLARATIONS OF INTEREST

### Declarations of interest

No conflicts of interest.

### Preliminary timeframe

Review to be completed by the end of June 2022.

### Plans for updating this review

Dependant on additional funding.

## SOURCES OF SUPPORT

### Internal sources

None, Other.

None provided.

### External sources

Centre for Homelessness Impact, UK.

Funding for systematic review.

## DIFFERENCES BETWEEN PROTOCOL AND REVIEW

In the typology of interventions outlined in Table 1 in the protocol, we listed self help programmes as an abstinence‐based intervention. We listed SMART Recovery as a form of self help programme. We were subsequently contacted by the SMART programme leaders, who explained that it is not abstinence based. We have therefore separately listed SMART Recovery programmes in the typology of interventions. This makes no difference to the findings or analysis as we did not find any effectiveness studies that met our inclusion criteria in relation to this intervention.

## Supporting information

Supporting information.

Supporting information.
