# Supplementary material for: The effectiveness of abstinence‐based and harm reduction‐based interventions in reducing problematic substance use in adults who are experiencing homelessness in high income countries: A systematic review and meta‐analysis: A systematic review
Source: Campbell Syst Rev. 2024 Apr 21;20(2):e1396. doi: 10.1002/cl2.1396 (PMC11032639; doi:10.1002/cl2.1396)
Supplement: Supplementary file 2 — Supporting information. [file CL2-20-e1396-s001.docx]

Characteristics of studies

Characteristics of included studies [ordered by study ID]

[Aubry 2016](#STD-Aubry-2016)

| ***Study characteristics*** | | |
| --- | --- | --- |
| Study design | Two-arm parallel RCT (individually randomised) | |
| Harm reduction or abstinence-based? | HR | |
| Primary intervention | ACT | |
| Longest intervention follow-up (months, N) | 21-24 months; n = 411 | |
| Control group | TAU | |
| Measure of interest | Self reported 7-item Global Assessment of Individual Need – Global Assessment of Individual Needs Short Screener–Substance Problem Scale. | |
| Source | EGM | |
| Notes |  | |
|  | | |
| **Item** | **Authors' judgement** | **Support for judgement** |
| 1a. Study design, end of intervention (Potential confounders taken into account) | Yes | RCT, RDD, ITS, instrumental variable |
| 2. Masking or blinding | No | Unblinded or no mention of blinding |
| 3. Power calculation | Yes | Mention of power calculation |
| 4a. Losses to follow up are presented and acceptable (End of intervention) | No | Attrition not reported, OR falls well outside WWC acceptable combined levels* |
| 5. Definition of intervention are clearly defined | Yes | Intervention clearly and fully described |
| 6. Outcome measures are clearly defined and reliable | Yes | Outcome measure clearly and fully described, preferably with reference to validation |
| 7. Baseline balance (N.A. for before versus after) | Yes | RCT or baseline balance report and satisfactory (imbalance on 2 or less measures) |
| Overall confidence in study (end of intervention) | No | Low confidence on any one of items 1a, 4a, 6 and 7 |
| Overall confidence- short | No | Low confidence |

[Aubry 2019](#STD-Aubry-2019)

| ***Study characteristics*** | | |
| --- | --- | --- |
| Study design | Two-arm parallel RCT (individually randomised) | |
| Harm reduction or abstinence-based? | HR | |
| Primary intervention | ACT | |
| Longest intervention follow-up (months, N) | 21-24 months; n = 90 | |
| Control group | TAU | |
| Measure of interest | Self reported Global Assessment of Individual Need – Global Assessment of Individual Needs Short Screener–Substance Problem Scale | |
| Source | EGM | |
| Notes |  | |
|  | | |
| **Item** | **Authors' judgement** | **Support for judgement** |
| 1a. Study design, end of intervention (Potential confounders taken into account) | Yes | RCT, RDD, ITS, instrumental variable |
| 1b. Study design, longest follow up if applicable (Potential confounders taken into account) | Yes | RCT, RDD, ITT, instrumental variable |
| 2. Masking or blinding | No | Unblinded or no mention of blinding |
| 3. Power calculation | Yes | Mention of power calculation |
| 4a. Losses to follow up are presented and acceptable (End of intervention) | No | Attrition not reported, OR falls well outside WWC acceptable combined levels* |
| 4b. Losses to follow up are presented and acceptable (Longest follow up, if applicable) | Yes | Overall and differential attrition within WWC combined levels* |
| 5. Definition of intervention are clearly defined | Yes | Intervention clearly and fully described |
| 6. Outcome measures are clearly defined and reliable | Yes | Outcome measure clearly and fully described, preferably with reference to validation |
| 7. Baseline balance (N.A. for before versus after) | Yes | RCT or baseline balance report and satisfactory (imbalance on 2 or less measures) |
| Overall confidence in study (end of intervention) | No | Low confidence on any one of items 1a, 4a, 6 and 8 |
| Overall confidence- short | No | Low confidence |

[Braucht 1995](#STD-Braucht-1995)

| ***Study characteristics*** | | |
| --- | --- | --- |
| Study design | Two-arm parallel RCT (individually randomised) | |
| Harm reduction or abstinence-based? | AB | |
| Primary intervention | ICM | |
| Longest intervention follow-up (months, N) | 6 months; n = 143 | |
| Control group | TAU | |
| Measure of interest | Range of substance use outcomes | |
| Source | Unpacked from SR | |
| Notes | Sample: Estimated based on: We were unable to track 10% and 13% of the control group at Times 2 and 3, respectively, and 4% and 12% of the experimental (ICM) group at these time points. Rounded down for analysis. Unable to calculate effect size. | |
|  | | |
| **Item** | **Authors' judgement** | **Support for judgement** |
| 1a. Study design, end of intervention (Potential confounders taken into account) | Yes | RCT, RDD, ITS, instrumental variable |
| 2. Masking or blinding | No | Unblinded or no mention of blinding |
| 3. Power calculation | Unclear | Medium: No discussion of power calculation |
| 4a. Losses to follow up are presented and acceptable (End of intervention) | Yes | Overall and differential attrition within WWC conservative levels* |
| 4b. Losses to follow up are presented and acceptable (Longest follow up, if applicable) | No | Attrition not reported, OR falls well outside WWC acceptable combined levels* |
| 5. Definition of intervention are clearly defined | Yes | Intervention clearly and fully described |
| 6. Outcome measures are clearly defined and reliable | Yes | Outcome measure clearly and fully described, preferably with reference to validation |
| 7. Baseline balance (N.A. for before versus after) | Yes | RCT or baseline balance report and satisfactory (imbalance on 2 or less measures) |
| Overall confidence in study (end of intervention) | No | Low confidence: Low on any one of items 1a, 4a, 6 and 6 |
| Overall confidence- short | No | Low confidence |

[Burnam 1995](#STD-Burnam-1995)

| ***Study characteristics*** | | |
| --- | --- | --- |
| Study design | Multi-arm parallel RCT (individually randomised) | |
| Harm reduction or abstinence-based? | AB | |
| Primary intervention | Group A; Residential rehabilitation + Abstinence based day programmes. Group B; Intensive Case Management + Abstinence based day programmes. | |
| Longest intervention follow-up (months, N) | Group A: 9 months; n = 45; Group B; 9 months; n = 110 | |
| Control group | TAU | |
| Measure of interest | Days used alcohol in last 30 days; Days used drugs in last 30 days | |
| Source | Unpacked from SR | |
| Notes | Unable to calculate effect size | |
|  | | |
| **Item** | **Authors' judgement** | **Support for judgement** |
| 1a. Study design, end of intervention (Potential confounders taken into account) | Yes | RCT, RDD, ITS, instrumental variable |
| 1b. Study design, longest follow up if applicable (Potential confounders taken into account) | Yes | RCT, RDD, ITS, instrumental variable |
| 2. Masking or blinding | No | Unblinded or no mention of blinding |
| 3. Power calculation | Unclear | Medium: No discussion of power calculation |
| 4a. Losses to follow up are presented and acceptable (End of intervention) | No | Attrition not reported, OR falls well outside WWC acceptable combined levels* |
| 4b. Losses to follow up are presented and acceptable (Longest follow up, if applicable) | No | Attrition not reported, OR falls well outside WWC acceptable combined levels* |
| 5. Definition of intervention are clearly defined | Yes | Intervention clearly and fully described |
| 6. Outcome measures are clearly defined and reliable | Yes | Outcome measure clearly and fully described, preferably with reference to validation |
| 7. Baseline balance (N.A. for before versus after) | Yes | RCT or baseline balance report and satisfactory (imbalance on 2 or less measures) |
| Overall confidence in study (end of intervention) | No | Low confidence: Low on any one of items 1a, 4a, 6 and 7 |
| Overall confidence- short | No | Low confidence |

[Cherner 2017](#STD-Cherner-2017)

| ***Study characteristics*** | | |
| --- | --- | --- |
| Study design | QED - DID | |
| Harm reduction or abstinence-based? | HR | |
| Primary intervention | ICM + OST | |
| Longest intervention follow-up (months, N) | 24 months; n = 78 | |
| Control group | TAU | |
| Measure of interest | Alcohol Use Disorders Identification Test (AUDIT) for alcohol use; Drug Abuse Screening Tool (DAST-10) for drug use - both self report at least weekly | |
| Source | Unpacked from SR | |
| Notes |  | |
|  | | |
| **Item** | **Authors' judgement** | **Support for judgement** |
| 1a. Study design, end of intervention (Potential confounders taken into account) | Unclear | Medium confidence: DiD with matching, PSM |
| 1b. Study design, longest follow up if applicable (Potential confounders taken into account) | Unclear | Medium confidence: DiD with matching, PSM |
| 2. Masking or blinding | No | Unblinded or no mention of blinding |
| 3. Power calculation | Yes | Mention of power calculation |
| 4a. Losses to follow up are presented and acceptable (End of intervention) | No | Attrition not reported, OR falls well outside WWC acceptable combined levels* |
| 4b. Losses to follow up are presented and acceptable (Longest follow up, if applicable) | No | Attrition not reported, OR falls well outside WWC acceptable combined levels* |
| 5. Definition of intervention are clearly defined | Yes | Intervention clearly and fully described |
| 6. Outcome measures are clearly defined and reliable | Yes | Outcome measure clearly and fully described, preferably with reference to validation |
| 7. Baseline balance (N.A. for before versus after) | No | No baseline balance test (except RCT) OR reported and significant differences on more than five measures. PSM without establishing common support |
| Overall confidence in study (end of intervention) | No | Low confidence: Low on any one of items 1a, 4a, 6 and 7 |
| Overall confidence- short | No | Low confidence |

[Chung 2018](#STD-Chung-2018)

| ***Study characteristics*** | | |
| --- | --- | --- |
| Study design | Two-arm parallel RCT (individually randomised) | |
| Harm reduction or abstinence-based? | HR | |
| Primary intervention | ACT or ICM | |
| Longest intervention follow-up (months, N) | 24 months; n = 1158 | |
| Control group | TAU | |
| Measure of interest | (GAIN-SS) Self reported five-item Substance Disorder Screener of the Global Assessment of Individual Needs Short Screener (GAIN) was used to assess the severity of substance use problems by counting the number of substance-related problems in the pastmonth (range 0–5, higher values indicating greater problem severity). GAIN-SS counts the number of substance-related problems in the past month; as a result, these values represent changes in rates not means | |
| Source | EGM | |
| Notes | Intervention split into 2 age groups for effect size calculation, but presented here as one combined group (14-49 years; Intervention n = 905, Control n = 773; >50 years; Intervention n = 253, Control n = 217) | |
|  | | |
| **Item** | **Authors' judgement** | **Support for judgement** |
| 1a. Study design, end of intervention (Potential confounders taken into account) | Yes | RCT, RDD, ITS, instrumental variable |
| 2. Masking or blinding | No | Unblinded or no mention of blinding |
| 3. Power calculation | Unclear | Medium: No discussion of power calculation |
| 4a. Losses to follow up are presented and acceptable (End of intervention) | Unclear | Medium: Overall and differential attrition within WWC liberal levels* |
| 5. Definition of intervention are clearly defined | Yes | Intervention clearly and fully described |
| 6. Outcome measures are clearly defined and reliable | Yes | Outcome measure clearly and fully described, preferably with reference to validation |
| 7. Baseline balance (N.A. for before versus after) | Yes | RCT or baseline balance report and satisfactory (imbalance on 2 or less measures) |
| Overall confidence in study (end of intervention) | Unclear | Medium confdience: Medium on any one of items 1a, 4a, 6 and 7 AND NOT LOW |
| Overall confidence- short | Unclear | Medium confidence |

[Clifasefi 2020](#STD-Clifasefi-2020)

| ***Study characteristics*** | | |
| --- | --- | --- |
| Study design | QED - other | |
| Harm reduction or abstinence-based? | HR | |
| Primary intervention | Group Work | |
| Longest intervention follow-up (months, N) | 6 months; n = 48 | |
| Control group | TAU | |
| Measure of interest | Alcohol Quantity and Use Assessment. Modified version of the Addiction Severity Index to assess for frequency. Short Inventory of Problems a 15‐item Likert‐scale questionnaire that yields a summary score reflecting participants’ past‐month experience of social, occupational,and psychological harm related to alcohol use. | |
| Source | EGM | |
| Notes | Unable to calculate effect size | |
|  | | |
| **Item** | **Authors' judgement** | **Support for judgement** |
| 1a. Study design, end of intervention (Potential confounders taken into account) | Unclear | Medium confidence: DiD with matching, PSM |
| 2. Masking or blinding | No | Unblinded or no mention of blinding |
| 3. Power calculation | Unclear | Medium confidence: No discussion of power calculation |
| 4a. Losses to follow up are presented and acceptable (End of intervention) | No | Attrition not reported, OR falls well outside WWC acceptable combined levels* |
| 5. Definition of intervention are clearly defined | Yes | Intervention clearly and fully described |
| 6. Outcome measures are clearly defined and reliable | Yes | Outcome measure clearly and fully described, preferably with reference to validation |
| 7. Baseline balance (N.A. for before versus after) | Unclear | Medium confidence: imbalance on no more than 5 measures |
| Overall confidence in study (end of intervention) | No | Low confidence: Low on any one of items 1a, 4a, 6 and 7 |
| Overall confidence- short | No | Low confidence |

[Collins 2019](#STD-Collins-2019)

| ***Study characteristics*** | | |
| --- | --- | --- |
| Study design | Two-arm parallel RCT (individually randomised) | |
| Harm reduction or abstinence-based? | HR | |
| Primary intervention | HRP + MI | |
| Longest intervention follow-up (months, N) | 3 months; n = 65 | |
| Control group | TAU | |
| Measure of interest | Number of days drinking to intoxication in last two weeks | |
| Source | Hand search | |
| Notes |  | |
|  | | |
| **Item** | **Authors' judgement** | **Support for judgement** |
| 1a. Study design, end of intervention (Potential confounders taken into account) | Yes | RCT, RDD, ITS, instrumental variable |
| 2. Masking or blinding | No | Unblinded or no mention of blinding |
| 3. Power calculation | Yes | Mention of power calculation |
| 4a. Losses to follow up are presented and acceptable (End of intervention) | No | Attrition not reported, OR falls well outside WWC acceptable combined levels* |
| 5. Definition of intervention are clearly defined | Yes | Intervention clearly and fully described |
| 6. Outcome measures are clearly defined and reliable | Yes | Outcome measure clearly and fully described, preferably with reference to validation |
| 7. Baseline balance (N.A. for before versus after) | Yes | RCT or baseline balance report and satisfactory (imbalance on 2 or less measures) |
| Overall confidence in study (end of intervention) | No | Low confidence: Low on any one of items 1a, 4a, 6 and 7 |
| Overall confidence- short | No | Low confidence |

[Collins 2021](#STD-Collins-2021)

| ***Study characteristics*** | | |
| --- | --- | --- |
| Study design | Multi-arm parallel RCT (individually randomised) | |
| Harm reduction or abstinence-based? | HR | |
| Primary intervention | Group A: Harm reduction psychotherapy + Motivational Interviewing (MI) + Prescripting to prevent relapse; Group B: Harm reduction psychotherapy + Motivational Interviewing (MI) (+ placebo); Group C: Harm reduction psychotherapy + Motivational Interviewing (MI) | |
| Longest intervention follow-up (months, N) | Group A: 9 months; n = 48; Group B: 9 months; n = 48; Group C: 9 months; n = 54 | |
| Control group | TAU | |
| Measure of interest | Self reported alcohol use - the number of standard (11·671 g) alcoholic drinks consumed on the heaviest drinking day in the past month; | |
| Source | EGM | |
| Notes | Same control group for all treatment groups | |
|  | | |
| **Item** | **Authors' judgement** | **Support for judgement** |
| 1a. Study design, end of intervention (Potential confounders taken into account) | Yes | RCT, RDD, ITS, instrumental variable |
| 1b. Study design, longest follow up if applicable (Potential confounders taken into account) | Yes | RCT, RDD, ITT, instrumental variable |
| 2. Masking or blinding | Yes | EITHER masking of of outcome measurement OR masking for analysis (or both) |
| 3. Power calculation | Yes | Mention of power calculation |
| 4a. Losses to follow up are presented and acceptable (End of intervention) | No | Attrition not reported, OR falls well outside WWC acceptable combined levels* |
| 4b. Losses to follow up are presented and acceptable (Longest follow up, if applicable) | No | Low: Attrition not reported, OR falls well outside WWC acceptable combined levels* |
| 5. Definition of intervention are clearly defined | Unclear | Medium: Brief description of intervention |
| 6. Outcome measures are clearly defined and reliable | Yes | Outcome measure clearly and fully described, preferably with reference to validation |
| 7. Baseline balance (N.A. for before versus after) | Yes | RCT or baseline balance report and satisfactory (imbalance on 2 or less measures) |
| Overall confidence in study (end of intervention) | No | Low confidence: Low on any one of items 1a, 4a, 6 and 7 |
| Overall confidence- short | No | Low confidence |

[Conrad 1998](#STD-Conrad-1998)

| ***Study characteristics*** | | |
| --- | --- | --- |
| Study design | Two-arm parallel RCT (individually randomised) | |
| Harm reduction or abstinence-based? | AB | |
| Primary intervention | Residential rehabilitation | |
| Longest intervention follow-up (months, N) | 24 month; n = 178 | |
| Control group | TAU | |
| Measure of interest | 6 items from Addiction severity Index (ASI) Alcohol AND 6 items from Addiction severity Index (ASI) Drug | |
| Source | EGM | |
| Notes |  | |
|  | | |
| **Item** | **Authors' judgement** | **Support for judgement** |
| 1a. Study design, end of intervention (Potential confounders taken into account) | Yes | RCT, RDD, ITS, instrumental variable |
| 1b. Study design, longest follow up if applicable (Potential confounders taken into account) | Yes | RCT, RDD, ITT, instrumental variable |
| 2. Masking or blinding | No | Unblinded or no mention of blinding |
| 3. Power calculation | Unclear | Medium: No discussion of power calculation |
| 4a. Losses to follow up are presented and acceptable (End of intervention) | No | Attrition not reported, OR falls well outside WWC acceptable combined levels* |
| 4b. Losses to follow up are presented and acceptable (Longest follow up, if applicable) | No | Attrition not reported, OR falls well outside WWC acceptable combined levels* |
| 5. Definition of intervention are clearly defined | Yes | Intervention clearly and fully described |
| 6. Outcome measures are clearly defined and reliable | Yes | Outcome measure clearly and fully described, preferably with reference to validation |
| 7. Baseline balance (N.A. for before versus after) | Yes | RCT or baseline balance report and satisfactory (imbalance on 2 or less measures) |
| Overall confidence in study (end of intervention) | No | Low confidence: Low on any one of items 1a, 4a, 6 and 7 |
| Overall confidence- short | No | Low confidence |

[Essock 2006](#STD-Essock-2006)

| ***Study characteristics*** | | |
| --- | --- | --- |
| Study design | Two-arm parallel RCT (individually randomised) | |
| Harm reduction or abstinence-based? | HR | |
| Primary intervention | ACT | |
| Longest intervention follow-up (months, N) | Site 1: 24 month; n = 50; Site 2: 24 month; n = 49 | |
| Control group | TAU | |
| Measure of interest | Substance Abuse Treatment Scale (SATS) - 8- point scale that indicates progressive movement toward treatment involvement, remission, and recovery from substance use disorders | |
| Source | EGM | |
| Notes |  | |
|  | | |
| **Item** | **Authors' judgement** | **Support for judgement** |
| 1a. Study design, end of intervention (Potential confounders taken into account) | Yes | RCT, RDD, ITS, instrumental variable |
| 2. Masking or blinding | No | Unblinded or no mention of blinding |
| 3. Power calculation | Unclear | Medium: No discussion of power calculation |
| 4a. Losses to follow up are presented and acceptable (End of intervention) | Yes | Overall and differential attrition within WWC conservative levels* |
| 5. Definition of intervention are clearly defined | Yes | Intervention clearly and fully described |
| 6. Outcome measures are clearly defined and reliable | Yes | Outcome measure clearly and fully described, preferably with reference to validation |
| 7. Baseline balance (N.A. for before versus after) | Yes | RCT or baseline balance report and satisfactory (imbalance on 2 or less measures) |
| Overall confidence in study (end of intervention) | Yes | High confidence: High on all of items 1a, 4a, 6 and 7 |
| Overall confidence- short | Yes | High confidence |

[Fletcher 2008](#STD-Fletcher-2008)

| ***Study characteristics*** | | |
| --- | --- | --- |
| Study design | Multi-arm parallel RCT (individually randomised) | |
| Harm reduction or abstinence-based? | HR | |
| Primary intervention | Treatment A: Integrated Assertive Community Therapy (IACT) Treatment B: Assertive Community Therapy (ACT) | |
| Longest intervention follow-up (months, N) | Treatment A: 20 months; n = 53; Treatment B: 20 months; n = 47 | |
| Control group | TAU | |
| Measure of interest | Substance Abuse Rating | |
| Source | Unpacked from SR | |
| Notes | Same control group for all treatment groups | |
|  | | |
| **Item** | **Authors' judgement** | **Support for judgement** |
| 1a. Study design, end of intervention (Potential confounders taken into account) | Yes | RCT, RDD, ITS, instrumental variable |
| 1b. Study design, longest follow up if applicable (Potential confounders taken into account) | Yes | RCT, RDD, ITS, instrumental variable |
| 2. Masking or blinding | No | Unblinded or no mention of blinding |
| 3. Power calculation | Unclear | Medium: No discussion of power calculation |
| 4a. Losses to follow up are presented and acceptable (End of intervention) | No | Attrition not reported, OR falls well outside WWC acceptable combined levels* |
| 4b. Losses to follow up are presented and acceptable (Longest follow up, if applicable) | Yes | Overall and differential attrition within WWC conservative levels* |
| 5. Definition of intervention are clearly defined | Yes | Intervention clearly and fully described |
| 6. Outcome measures are clearly defined and reliable | Yes | Outcome measure clearly and fully described, preferably with reference to validation |
| 7. Baseline balance (N.A. for before versus after) | Yes | RCT or baseline balance report and satisfactory (imbalance on 2 or less measures) |
| Overall confidence in study (end of intervention) | No | Low confidence: Low on any one of items 1a, 4a, 6 and 7 |
| Overall confidence- short | No | Low confidence |

[Harpaz-Rotem 2011](#STD-Harpaz_x002d_Rotem-2011)

| ***Study characteristics*** | | |
| --- | --- | --- |
| Study design | QED - other | |
| Harm reduction or abstinence-based? | AB | |
| Primary intervention | Residential rehabilitation | |
| Longest intervention follow-up (months, N) | 12 months; n = 96 | |
| Control group | Other intervention - No or less than 30 days RT (NRT) group | |
| Measure of interest | Addiction severity Index (ASI) Drugs AND Addiction severity Index (ASI) Alcohol | |
| Source | EGM | |
| Notes |  | |
|  | | |
| **Item** | **Authors' judgement** | **Support for judgement** |
| 1a. Study design, end of intervention (Potential confounders taken into account) | Unclear | Medium confidence: DiD with matching, PSM |
| 2. Masking or blinding | No | Unblinded or no mention of blinding |
| 3. Power calculation | Unclear | Medium: No discussion of power calculation |
| 4a. Losses to follow up are presented and acceptable (End of intervention) | No | Attrition not reported, OR falls well outside WWC acceptable combined levels* |
| 5. Definition of intervention are clearly defined | Yes | Intervention clearly and fully described |
| 6. Outcome measures are clearly defined and reliable | Yes | Outcome measure clearly and fully described, preferably with reference to validation |
| 7. Baseline balance (N.A. for before versus after) | Unclear | Medium confidence: imbalance on no more than 5 measures |
| Overall confidence in study (end of intervention) | No | Low confidence: Low on any one of items 1a, 4a, 6 and 7 |
| Overall confidence- short | No | Low confidence |

[Kashner 2002](#STD-Kashner-2002)

| ***Study characteristics*** | | |
| --- | --- | --- |
| Study design | Two-arm parallel RCT (individually randomised) | |
| Harm reduction or abstinence-based? | AB | |
| Primary intervention | CM | |
| Longest intervention follow-up (months, N) | 12 months; n = 111 | |
| Control group | TAU | |
| Measure of interest | Addiction severity Index (ASI) Drugs AND Addiction severity Index (ASI) Alcohol | |
| Source | EGM | |
| Notes |  | |
|  | | |
| **Item** | **Authors' judgement** | **Support for judgement** |
| 1a. Study design, end of intervention (Potential confounders taken into account) | Yes | RCT, RDD, ITS, instrumental variable |
| 2. Masking or blinding | No | Unblinded or no mention of blinding |
| 3. Power calculation | Unclear | Medium: No discussion of power calculation |
| 4a. Losses to follow up are presented and acceptable (End of intervention) | Yes | Overall and differential attrition within WWC conservative levels* |
| 5. Definition of intervention are clearly defined | Yes | Intervention clearly and fully described |
| 6. Outcome measures are clearly defined and reliable | Yes | Outcome measure clearly and fully described, preferably with reference to validation |
| 7. Baseline balance (N.A. for before versus after) | Yes | RCT or baseline balance report and satisfactory (imbalance on 2 or less measures) |
| Overall confidence in study (end of intervention) | Yes | High confidence: High on all of items 1a, 4a, 6 and 7 |
| Overall confidence- short | Yes | High confidence |

[Kirst 2015](#STD-Kirst-2015)

| ***Study characteristics*** | | |
| --- | --- | --- |
| Study design | Two-arm parallel RCT (individually randomised) | |
| Harm reduction or abstinence-based? | HR | |
| Primary intervention | ACT or ICM | |
| Longest intervention follow-up (months, N) | 24 months; n = 301 | |
| Control group | TAU | |
| Measure of interest | Alcohol problems in 30 days; Drug problems in 30 days; GAIN-SS - The GAIN-SS consisted of five questions to determine participants’ severity of substance use problems (such as getting into fights, problems at work, dealing with withdrawal symptoms) in the ‘past month’, ‘2–12 Months’ or ‘1 or more years.’ | |
| Source | EGM | |
| Notes |  | |
|  | | |
| **Item** | **Authors' judgement** | **Support for judgement** |
| 1a. Study design, end of intervention (Potential confounders taken into account) | Yes | RCT, RDD, ITS, instrumental variable |
| 2. Masking or blinding | No | Unblinded or no mention of blinding |
| 3. Power calculation | Unclear | Medium: No discussion of power calculation |
| 4a. Losses to follow up are presented and acceptable (End of intervention) | No | Attrition not reported, OR falls well outside WWC acceptable combined levels* |
| 5. Definition of intervention are clearly defined | Yes | Intervention clearly and fully described |
| 6. Outcome measures are clearly defined and reliable | Yes | Outcome measure clearly and fully described, preferably with reference to validation |
| 7. Baseline balance (N.A. for before versus after) | Yes | RCT or baseline balance report and satisfactory (imbalance on 2 or less measures) |
| Overall confidence in study (end of intervention) | No | Low confidence: Low on any one of items 1a, 4a, 6 and 7 |
| Overall confidence- short | No | Low confidence |

[Koffarnus 2011](#STD-Koffarnus-2011)

| ***Study characteristics*** | | |
| --- | --- | --- |
| Study design | Multi-arm parallel RCT (individually randomised) | |
| Harm reduction or abstinence-based? | AB | |
| Primary intervention | CM | |
| Longest intervention follow-up (months, N) | 6 months; n = 43 | |
| Control group | Other intervention - Group A: Job skills training (Unpaid) Group B: Paid Training Group | |
| Measure of interest | Percent BAL ≥ 0.004 (MM) - Blood Alocohol level positive for alcohol AND Percent BAL ≥ 0.05 g/dl (which represents a level with more alcohol-related impairment) AND Number of days of heavy drinking (self-report) - he/she reported consuming more than four drinks (females) or five drinks (males) in the 24 h prior to a random assessment or during at least one day in the month prior to a monthly assessment | |
| Source |  | |
| Notes |  | |
|  | | |
| **Item** | **Authors' judgement** | **Support for judgement** |
| 1a. Study design, end of intervention (Potential confounders taken into account) | Yes | RCT, RDD, ITS, instrumental variable |
| 2. Masking or blinding | Yes | EITHER masking of of outcome measurement OR masking for analysis (or both) |
| 3. Power calculation | Yes | Mention of power calculation |
| 4a. Losses to follow up are presented and acceptable (End of intervention) | No | Attrition not reported, OR falls well outside WWC acceptable combined levels* |
| 5. Definition of intervention are clearly defined | Yes | Intervention clearly and fully described |
| 6. Outcome measures are clearly defined and reliable | Yes | Outcome measure clearly and fully described, preferably with reference to validation |
| 7. Baseline balance (N.A. for before versus after) | Yes | RCT or baseline balance report and satisfactory (imbalance on 2 or less measures) |
| Overall confidence in study (end of intervention) | No | Low confidence: Low on any one of items 1a, 4a, 6 and 7 |
| Overall confidence- short | No | Low confidence |

[Lapham 1995](#STD-Lapham-1995)

| ***Study characteristics*** | | |
| --- | --- | --- |
| Study design | Multi-arm parallel RCT (individually randomised) | |
| Harm reduction or abstinence-based? | AB | |
| Primary intervention | Intensive Case Management (ICM) - All treatment groups start with residential detoxification. Group A is described as housing plus case management, which (outlined in another paper by the authors) includes direct provision of substance use treatment. The other two group (B and C) involve housing and peer support, or just housing. | |
| Longest intervention follow-up (months, N) | Group A: 10 months; n = 135; Group B; 10 months; n = 123; Group C; 10 months; n = 74 | |
| Control group | TAU | |
| Measure of interest | Alcohol use days - past 30 days | |
| Source | EGM | |
| Notes |  | |
|  | | |
| **Item** | **Authors' judgement** | **Support for judgement** |
| 1a. Study design, end of intervention (Potential confounders taken into account) | Yes | RCT, RDD, ITS, instrumental variable |
| 1b. Study design, longest follow up if applicable (Potential confounders taken into account) | Yes | RCT, RDD, ITS, instrumental variable |
| 2. Masking or blinding | No | Unblinded or no mention of blinding |
| 3. Power calculation | Unclear | Medium: No discussion of power calculation |
| 4a. Losses to follow up are presented and acceptable (End of intervention) | Yes | Overall and differential attrition within WWC conservative levels* |
| 4b. Losses to follow up are presented and acceptable (Longest follow up, if applicable) | Yes | Overall and differential attrition within WWC conservative levels* |
| 5. Definition of intervention are clearly defined | Yes | Intervention clearly and fully described |
| 6. Outcome measures are clearly defined and reliable | Yes | Outcome measure clearly and fully described, preferably with reference to validation |
| 7. Baseline balance (N.A. for before versus after) | Unclear | Medium confidence: imbalance on no more than 5 measures |
| Overall confidence in study (end of intervention) | Unclear | Medium: Medium on any one of items 1a, 4a, 6 and 7 AND NOT LOW |
| Overall confidence- short | Unclear | Medium confidence |

[McHugo 2004](#STD-McHugo-2004)

| ***Study characteristics*** | | |
| --- | --- | --- |
| Study design | Two-arm parallel RCT (individually randomised) | |
| Harm reduction or abstinence-based? | HR | |
| Primary intervention | ACT | |
| Longest intervention follow-up (months, N) | 18 months; n = see notes | |
| Control group | Other Intervention - Integrated housing program where case management and housing services were provided by teams within a single agency and were closely coordinated. | |
| Measure of interest | Days of illicit drug use in past 6 months AND Days of alcohol use in past 6 months | |
| Source | Unpacked from SR | |
| Notes | Total data recorded varied for outcome measure (Drugs n = 35; Alcohol n = 53) - no clear data about intervention and control particpant distribution | |
|  | | |
| **Item** | **Authors' judgement** | **Support for judgement** |
| 1a. Study design, end of intervention (Potential confounders taken into account) | Yes | RCT, RDD, ITS, instrumental variable |
| 1b. Study design, longest follow up if applicable (Potential confounders taken into account) | Yes | RCT, RDD, ITT, instrumental variable |
| 2. Masking or blinding | No | Unblinded or no mention of blinding |
| 3. Power calculation | Yes | Mention of power calculation |
| 4a. Losses to follow up are presented and acceptable (End of intervention) | Unclear | Medium: Overall and differential attrition within WWC liberal levels* |
| 5. Definition of intervention are clearly defined | Yes | Intervention clearly and fully described |
| 6. Outcome measures are clearly defined and reliable | Yes | Outcome measure clearly and fully described, preferably with reference to validation |
| 7. Baseline balance (N.A. for before versus after) | Yes | RCT or baseline balance report and satisfactory (imbalance on 2 or less measures) |
| Overall confidence in study (end of intervention) | Unclear | Medium: Medium on any one of items 1a, 4a, 6 and 7 AND NOT LOW |
| Overall confidence- short | Unclear | Medium confidence |

[Milby 1996](#STD-Milby-1996)

| ***Study characteristics*** | | |
| --- | --- | --- |
| Study design | Two-arm parallel RCT (individually randomised) | |
| Harm reduction or abstinence-based? | AB | |
| Primary intervention | Contingency Management (CM)+ Abstinence based day programmes | |
| Longest intervention follow-up (months, N) | 12 months; n = 69 | |
| Control group | TAU | |
| Measure of interest | Alcohol use in the last 30 days AND EMIT urine toxicologies - proportion of positive toxologies for cocaine | |
| Source | EGM | |
| Notes |  | |
|  | | |
| **Item** | **Authors' judgement** | **Support for judgement** |
| 1a. Study design, end of intervention (Potential confounders taken into account) | Yes | RCT, RDD, ITS, instrumental variable |
| 2. Masking or blinding | Yes | EITHER masking of of outcome measurement OR masking for analysis (or both) |
| 3. Power calculation | Unclear | Medium: No discussion of power calculation |
| 4a. Losses to follow up are presented and acceptable (End of intervention) | Yes | Overall and differential attrition within WWC conservative levels* |
| 5. Definition of intervention are clearly defined | No | Intervention named but not described, or not named |
| 6. Outcome measures are clearly defined and reliable | Yes | Outcome measure clearly and fully described, preferably with reference to validation |
| 7. Baseline balance (N.A. for before versus after) | No | No baseline balance test (except RCT) OR reported and significant differences on more than five measures. PSM without establishing common support |
| Overall confidence in study (end of intervention) | No | Low confidence: Low on any one of items 1a, 4a, 6 and 7 |
| Overall confidence- short | No | Low confidence |

[Milby 2000](#STD-Milby-2000)

| ***Study characteristics*** | | |
| --- | --- | --- |
| Study design | Two-arm parallel RCT (individually randomised) | |
| Harm reduction or abstinence-based? | AB | |
| Primary intervention | CM | |
| Longest intervention follow-up (months, N) | 6 months; n = 56 | |
| Control group | Other intervention - Behavioral day treatment alone (DT). | |
| Measure of interest | Abstinence: Percentage of negative random urine toxicologies in the past 60 days for all substances tested (percentage of days abstinent in the last 60 days) | |
| Source | EGM | |
| Notes |  | |
|  | | |
| **Item** | **Authors' judgement** | **Support for judgement** |
| 1a. Study design, end of intervention (Potential confounders taken into account) | Yes | RCT, RDD, ITS, instrumental variable |
| 2. Masking or blinding | No | Unblinded or no mention of blinding |
| 3. Power calculation | Unclear | Medium: No discussion of power calculation |
| 4a. Losses to follow up are presented and acceptable (End of intervention) | Yes | Overall and differential attrition within WWC conservative levels* |
| 5. Definition of intervention are clearly defined | Yes | Intervention clearly and fully described |
| 6. Outcome measures are clearly defined and reliable | Yes | Outcome measure clearly and fully described, preferably with reference to validation |
| 7. Baseline balance (N.A. for before versus after) | Yes | RCT or baseline balance report and satisfactory (imbalance on 2 or less measures) |
| Overall confidence in study (end of intervention) | Yes | High confidence: High on all of items 1a, 4a, 6 and 7 |
| Overall confidence- short | Yes | High confidence |

[Milby 2003](#STD-Milby-2003)

| ***Study characteristics*** | | |
| --- | --- | --- |
| Study design | Two-arm parallel RCT (individually randomised) | |
| Harm reduction or abstinence-based? | AB | |
| Primary intervention | CM | |
| Longest intervention follow-up (months, N) | 12 months; n = 57 | |
| Control group | Other intervention - Behavioral day treatment alone (DT) | |
| Measure of interest | Abstinence prevalance: Binary outcome using information from the assessment point urine toxicology tests AND drug use in the last 30 days self-reported on the Addiction Severity Index | |
| Source | EGM | |
| Notes |  | |
|  | | |
| **Item** | **Authors' judgement** | **Support for judgement** |
| 1a. Study design, end of intervention (Potential confounders taken into account) | Yes | RCT, RDD, ITS, instrumental variable |
| 2. Masking or blinding | No | Unblinded or no mention of blinding |
| 3. Power calculation | Unclear | Medium: No discussion of power calculation |
| 4a. Losses to follow up are presented and acceptable (End of intervention) | No | Attrition not reported, OR falls well outside WWC acceptable combined levels* |
| 5. Definition of intervention are clearly defined | Yes | Intervention clearly and fully described |
| 6. Outcome measures are clearly defined and reliable | Yes | Outcome measure clearly and fully described, preferably with reference to validation |
| 7. Baseline balance (N.A. for before versus after) | Yes | RCT or baseline balance report and satisfactory (imbalance on 2 or less measures) |
| Overall confidence in study (end of intervention) | No | Low confidence: Low on any one of items 1a, 4a, 6 and 7 |
| Overall confidence- short | No | Low confidence |

[Morse 1992](#STD-Morse-1992)

| ***Study characteristics*** | | |
| --- | --- | --- |
| Study design | Multi-arm parallel RCT (individually randomised) | |
| Harm reduction or abstinence-based? | HR | |
| Primary intervention | ACT | |
| Longest intervention follow-up (months, N) | 12 months; n = 36 | |
| Control group | Other intervention - Traditional outpatient treatment offered by a mental health clinic | |
| Measure of interest | Alcohol consumption (ounces per week) - Monthly quantity and frequency of alcohol consumption was measured using an index based on a form developed by the National Institute on Alcohol Abuse and Alcoholism | |
| Source | EGM | |
| Notes |  | |
|  | | |
| **Item** | **Authors' judgement** | **Support for judgement** |
| 1a. Study design, end of intervention (Potential confounders taken into account) | Yes | RCT, RDD, ITS, instrumental variable |
| 2. Masking or blinding | No | Unblinded or no mention of blinding |
| 3. Power calculation | Unclear | Medium: No discussion of power calculation |
| 4a. Losses to follow up are presented and acceptable (End of intervention) | Unclear | Medium: Overall and differential attrition within WWC liberal levels* |
| 5. Definition of intervention are clearly defined | Yes | Intervention clearly and fully described |
| 6. Outcome measures are clearly defined and reliable | Yes | Outcome measure clearly and fully described, preferably with reference to validation |
| 7. Baseline balance (N.A. for before versus after) | Yes | RCT or baseline balance report and satisfactory (imbalance on 2 or less measures) |
| Overall confidence in study (end of intervention) | Unclear | Medium confidence: Medium on any one of items 1a, 4a, 6 and 7 AND NOT LOW |
| Overall confidence- short | Unclear | Medium confidence |

[Morse 1997](#STD-Morse-1997)

| ***Study characteristics*** | | |
| --- | --- | --- |
| Study design | Multi-arm parallel RCT (individually randomised) | |
| Harm reduction or abstinence-based? | Unclear | |
| Primary intervention | Group A: Broker case management; Group B: ACT only (ACTO); Group C: Amended version of ACT | |
| Longest intervention follow-up (months, N) | Group A: 18 months; n = Group B: 18 months; n = Group C:18 months; n = | |
| Control group | Comparison of 3 treament groups | |
| Measure of interest | Days abused substances in past month AND ASI Alcohol AND ASI drugs | |
| Source | Unpacked from SR | |
| Notes |  | |
|  | | |
| **Item** | **Authors' judgement** | **Support for judgement** |
| 1a. Study design, end of intervention (Potential confounders taken into account) | Yes | RCT, RDD, ITS, instrumental variable |
| 2. Masking or blinding | No | Unblinded or no mention of blinding |
| 3. Power calculation | Unclear | Medium: No discussion of power calculation |
| 4a. Losses to follow up are presented and acceptable (End of intervention) | Unclear | Medium: Overall and differential attrition within WWC liberal levels* |
| 5. Definition of intervention are clearly defined | Yes | Intervention clearly and fully described |
| 6. Outcome measures are clearly defined and reliable | Yes | Outcome measure clearly and fully described, preferably with reference to validation |
| 7. Baseline balance (N.A. for before versus after) | Yes | RCT or baseline balance report and satisfactory (imbalance on 2 or less measures) |
| Overall confidence in study (end of intervention) | Unclear | Medium confidence: Medium on any one of items 1a, 4a, 6 and 7 AND NOT LOW |
| Overall confidence- short | Unclear | Medium confidence |

[Morse 2006](#STD-Morse-2006)

| ***Study characteristics*** | | |
| --- | --- | --- |
| Study design | Two-arm parallel RCT (individually randomised) | |
| Harm reduction or abstinence-based? | HR | |
| Primary intervention | Group A: Integrated ACT; Group B: ACT only | |
| Longest intervention follow-up (months, N) | Group A: 24 months; n = 46; Group B: 24 months; n = 54 | |
| Control group | TAU | |
| Measure of interest | Severity of both alcohol and drug use with two 5-point scales that have been used in many previous studies (Carey, Coco, & Simmons,1996; Drake, Osher, & Wallach, 1989). Higher scores indicated greater severity with 1 = client has not used alcohol (or drugs), and 5 = meets criteria for severe use plus related problems are so severe that make non-institutional living difficul | |
| Source | EGM | |
| Notes |  | |
|  | | |
| **Item** | **Authors' judgement** | **Support for judgement** |
| 1a. Study design, end of intervention (Potential confounders taken into account) | Yes | RCT, RDD, ITS, instrumental variable |
| 2. Masking or blinding | No | Unblinded or no mention of blinding |
| 3. Power calculation | Unclear | Medium: No discussion of power calculation |
| 4a. Losses to follow up are presented and acceptable (End of intervention) | No | Attrition not reported, OR falls well outside WWC acceptable combined levels* |
| 5. Definition of intervention are clearly defined | Yes | Intervention clearly and fully described |
| 6. Outcome measures are clearly defined and reliable | Yes | Outcome measure clearly and fully described, preferably with reference to validation |
| 7. Baseline balance (N.A. for before versus after) | Yes | RCT or baseline balance report and satisfactory (imbalance on 2 or less measures) |
| Overall confidence in study (end of intervention) | No | Low confidence: Low on any one of items 1a, 4a, 6 and 7 |
| Overall confidence- short | No | Low confidence |

[Morse 2008](#STD-Morse-2008)

| ***Study characteristics*** | | |
| --- | --- | --- |
| Study design | QED - other | |
| Harm reduction or abstinence-based? | HR | |
| Primary intervention | ACT | |
| Longest intervention follow-up (months, N) | 18 months; n = 79 | |
| Control group | TAU | |
| Measure of interest | Carey Rating — for both Alcohol AND drug abuse. These 5-point scales are scored such that higher scores indicate greater severity, with 1 indicating that the client has not used alcohol (or drugs) and 5 indicating that the client meets criteria for severe use and related problems are so severe that noninstitutional living would be difficult. | |
| Source | EGM | |
| Notes | Sample size reported as 85 for NIACT treatment group in abstract, but as 79 in main paper. | |
|  | | |
| **Item** | **Authors' judgement** | **Support for judgement** |
| 1a. Study design, end of intervention (Potential confounders taken into account) | Unclear | Medium confidence: DiD with matching, PSM |
| 2. Masking or blinding | No | Unblinded or no mention of blinding |
| 3. Power calculation | Unclear | Medium: No discussion of power calculation |
| 4a. Losses to follow up are presented and acceptable (End of intervention) | No | Attrition not reported, OR falls well outside WWC acceptable combined levels* |
| 5. Definition of intervention are clearly defined | Yes | Intervention clearly and fully described |
| 6. Outcome measures are clearly defined and reliable | Unclear | Medium: Brief description of outcome |
| 7. Baseline balance (N.A. for before versus after) | No | No baseline balance test (except RCT) OR reported and significant differences on more than five measures. PSM without establishing common support |
| Overall confidence in study (end of intervention) | No | Low confidence: Low on any one of items 1a, 4a, 6 and 7 |
| Overall confidence- short | No | Low confidence |

[Nyamathi 2016](#STD-Nyamathi-2016)

| ***Study characteristics*** | | |
| --- | --- | --- |
| Study design | Multi-arm parallel RCT (individually randomised) | |
| Harm reduction or abstinence-based? | HR | |
| Primary intervention | ICM | |
| Longest intervention follow-up (months, N) | Group A: 12 months; n = 166; Group B: 12 months; n = 177 | |
| Control group | TAU | |
| Measure of interest | 3 measures for drug use; 1. Marijuana - having used marijuana during the 12-month, stimulants - having used this drug during the 12- month observation period AND heroin - having used this drug during the 12-month observation period | |
| Source | EGM | |
| Notes | Intervention described as: Group A: Intensive peer coach and nurse case managed (PC-NCM) program. Group B: Intermediate peer coaching (PC) program with brief nurse counseling. Although not described as such, we have treated as ICM | |
|  | | |
| **Item** | **Authors' judgement** | **Support for judgement** |
| 1a. Study design, end of intervention (Potential confounders taken into account) | Yes | RCT, RDD, ITS, instrumental variable |
| 1b. Study design, longest follow up if applicable (Potential confounders taken into account) | Yes | RCT, RDD, ITS, instrumental variable |
| 2. Masking or blinding | No | Unblinded or no mention of blinding |
| 3. Power calculation | Unclear | Medium: No discussion of power calculation |
| 4a. Losses to follow up are presented and acceptable (End of intervention) | No | Attrition not reported, OR falls well outside WWC acceptable combined levels* |
| 4b. Losses to follow up are presented and acceptable (Longest follow up, if applicable) | No | Attrition not reported, OR falls well outside WWC acceptable combined levels* |
| 5. Definition of intervention are clearly defined | Yes | Intervention clearly and fully described |
| 6. Outcome measures are clearly defined and reliable | Yes | Outcome measure clearly and fully described, preferably with reference to validation |
| 7. Baseline balance (N.A. for before versus after) | Yes | RCT or baseline balance report and satisfactory (imbalance on 2 or less measures) |
| Overall confidence in study (end of intervention) | No | Low confidence: Low on any one of items 1a, 4a, 6 and 7 |
| Overall confidence- short | No | Low confidence |

[Nyamathi 2017](#STD-Nyamathi-2017)

| ***Study characteristics*** | | |
| --- | --- | --- |
| Study design | Two-arm parallel RCT (individually randomised) | |
| Harm reduction or abstinence-based? | Unclear | |
| Primary intervention | Talking therapies (CBT) + Group Work | |
| Longest intervention follow-up (months, N) | 6 months; n = 58 | |
| Control group | Other intervention - Health Promotion sessions | |
| Measure of interest | Drug and alcohol use abstinance (self-report confirmed by urine analysis) - test cup screened for metabolites of mphetamines, cocaine, ethamphetamines, 3,4- ethylenedioxymethamphetamine, opiates, and marijuana. | |
| Source | EGM | |
| Notes |  | |
|  | | |
| **Item** | **Authors' judgement** | **Support for judgement** |
| 1a. Study design, end of intervention (Potential confounders taken into account) | Yes | RCT, RDD, ITS, instrumental variable |
| 2. Masking or blinding | No | Unblinded or no mention of blinding |
| 3. Power calculation | Unclear | Medium: No discussion of power calculation |
| 4a. Losses to follow up are presented and acceptable (End of intervention) | No | Attrition not reported, OR falls well outside WWC acceptable combined levels* |
| 5. Definition of intervention are clearly defined | Yes | Intervention clearly and fully described |
| 6. Outcome measures are clearly defined and reliable | Yes | Outcome measure clearly and fully described, preferably with reference to validation |
| 7. Baseline balance (N.A. for before versus after) | Yes | RCT or baseline balance report and satisfactory (imbalance on 2 or less measures) |
| Overall confidence in study (end of intervention) | No | Low confidence: Low on any one of items 1a, 4a, 6 and 7 |
| Overall confidence- short | No | Low confidence |

[O'Connell 2012](#STD-O_x0027_Connell-2012)

| ***Study characteristics*** | | |
| --- | --- | --- |
| Study design | Multi-arm parallel RCT (individually randomised) | |
| Harm reduction or abstinence-based? | HR | |
| Primary intervention | Group A: ICM plus the HUD-VA Supported Housing (HUDVASH); Group B: Intensive case management (ICM) only. | |
| Longest intervention follow-up (months, N) | Group A: 24 months; n = 119; Group B: 24 months; n = 52 | |
| Control group | TAU | |
| Measure of interest | ASI Drugs AND ASI alcohol | |
| Source | EGM | |
| Notes |  | |
|  | | |
| **Item** | **Authors' judgement** | **Support for judgement** |
| 1a. Study design, end of intervention (Potential confounders taken into account) | Yes | RCT, RDD, ITS, instrumental variable |
| 2. Masking or blinding | No | Unblinded or no mention of blinding |
| 3. Power calculation | Unclear | Medium: No discussion of power calculation |
| 4a. Losses to follow up are presented and acceptable (End of intervention) | No | Attrition not reported, OR falls well outside WWC acceptable combined levels* |
| 5. Definition of intervention are clearly defined | Unclear | Medium: Brief description of intervention |
| 6. Outcome measures are clearly defined and reliable | Unclear | Medium: Brief description of outcome |
| 7. Baseline balance (N.A. for before versus after) | Yes | RCT or baseline balance report and satisfactory (imbalance on 2 or less measures) |
| Overall confidence in study (end of intervention) | No | Low confidence: Low on any one of items 1a, 4a, 6 and 7 |
| Overall confidence- short | No | Low confidence |

[Reback 2010](#STD-Reback-2010)

| ***Study characteristics*** | | |
| --- | --- | --- |
| Study design | Two-arm parallel RCT (individually randomised) | |
| Harm reduction or abstinence-based? | AB | |
| Primary intervention | CM | |
| Longest intervention follow-up (months, N) | 12 months; n = 64 | |
| Control group | Other intervention | |
| Measure of interest | Urine testing for substance use - Proportion of participants that provided drug-free urine samples at a specified time point divided by number of participants randomized to that condition AND breath test for alcohol AND Composite scores - Level 1 TES: cocaine, amphetamines, methamphetamines, PCP, and alcohol metabolite-free. Proportion free of all | |
| Source | EGM | |
| Notes | All participants earned points for participation in group tasks (meetings, follow-up interviews etc) but control group did not receive points for health promtoing behaviours + drug/alcohol abstinence | |
|  | | |
| **Item** | **Authors' judgement** | **Support for judgement** |
| 1a. Study design, end of intervention (Potential confounders taken into account) | Yes | RCT, RDD, ITS, instrumental variable |
| 2. Masking or blinding | No | Unblinded or no mention of blinding |
| 3. Power calculation | Unclear | No discussion of power calculation |
| 4a. Losses to follow up are presented and acceptable (End of intervention) | Yes | Overall and differential attrition within WWC conservative levels* |
| 5. Definition of intervention are clearly defined | Yes | Intervention clearly and fully described |
| 6. Outcome measures are clearly defined and reliable | Yes | Outcome measure clearly and fully described, preferably with reference to validation |
| 7. Baseline balance (N.A. for before versus after) | Yes | RCT or baseline balance report and satisfactory (imbalance on 2 or less measures) |
| Overall confidence in study (end of intervention) | Yes | High confidence: High on all of items 1a, 4a, 6 and 7 |
| Overall confidence- short | Yes | High confidence |

[Rosenheck 2003](#STD-Rosenheck-2003)

| ***Study characteristics*** | | |
| --- | --- | --- |
| Study design | Multi-arm parallel RCT (individually randomised) | |
| Harm reduction or abstinence-based? | HR | |
| Primary intervention | Group A: ICM plus the HUD-VA Supported Housing (HUDVASH); Group B: Intensive case management (ICM) only. | |
| Longest intervention follow-up (months, N) | Group A: 36 months; n = 182; Group B: 36 months; n = 90 | |
| Control group | TAU | |
| Measure of interest | ASI Drugs AND ASI alcohol | |
| Source | EGM | |
| Notes |  | |
|  | | |
| **Item** | **Authors' judgement** | **Support for judgement** |
| 1a. Study design, end of intervention (Potential confounders taken into account) | Yes | RCT, RDD, ITS, instrumental variable |
| 2. Masking or blinding | No | Unblinded or no mention of blinding |
| 3. Power calculation | Unclear | Medium: No discussion of power calculation |
| 4a. Losses to follow up are presented and acceptable (End of intervention) | No | Attrition not reported, OR falls well outside WWC acceptable combined levels* |
| 5. Definition of intervention are clearly defined | Yes | Intervention clearly and fully described |
| 6. Outcome measures are clearly defined and reliable | Yes | Outcome measure clearly and fully described, preferably with reference to validation |
| 7. Baseline balance (N.A. for before versus after) | Yes | RCT or baseline balance report and satisfactory (imbalance on 2 or less measures) |
| Overall confidence in study (end of intervention) | No | Low confidence: Low on any one of items 1a, 4a, 6 and 7 |
| Overall confidence- short | No | Low confidence |

[Schumacher 2000](#STD-Schumacher-2000)

| ***Study characteristics*** | | |
| --- | --- | --- |
| Study design | Two-arm parallel RCT (individually randomised) | |
| Harm reduction or abstinence-based? | AB | |
| Primary intervention | CM + day treatment | |
| Longest intervention follow-up (months, N) | 2 months; n = 72 | |
| Control group | Other intervention - Behavioral Day Treatment. . | |
| Measure of interest | Number of days attended | |
| Source | EGM | |
| Notes |  | |
|  | | |
| **Item** | **Authors' judgement** | **Support for judgement** |
| 1a. Study design, end of intervention (Potential confounders taken into account) | Yes | RCT, RDD, ITS, instrumental variable |
| 2. Masking or blinding | No | Unblinded or no mention of blinding |
| 3. Power calculation | Unclear | No discussion of power calculation |
| 4a. Losses to follow up are presented and acceptable (End of intervention) | Yes | Overall and differential attrition within WWC conservative levels* |
| 5. Definition of intervention are clearly defined | Yes | Intervention clearly and fully described |
| 6. Outcome measures are clearly defined and reliable | Yes | Outcome measure clearly and fully described, preferably with reference to validation |
| 7. Baseline balance (N.A. for before versus after) | Yes | RCT or baseline balance report and satisfactory (imbalance on 2 or less measures) |
| Overall confidence in study (end of intervention) | Yes | High confidence: High on all of items 1a, 4a, 6 and 7 |
| Overall confidence- short | Yes | High confidence |

[Schumacher 2003](#STD-Schumacher-2003)

| ***Study characteristics*** | | |
| --- | --- | --- |
| Study design | Two-arm parallel RCT (individually randomised) | |
| Harm reduction or abstinence-based? | AB | |
| Primary intervention | CM + day treatment (DT) | |
| Longest intervention follow-up (months, N) | 6 months; n = 45 | |
| Control group | Other intervention - Behavioral Day Treatment. | |
| Measure of interest | Positive DSM outcome, "good or improved" (all drugs - alcohol, cocaine, marijuana) AND Continuous abstinence (consecutive weeks of abstinence by means of assay over 6 months) | |
| Source | EGM | |
| Notes |  | |
|  | | |
| **Item** | **Authors' judgement** | **Support for judgement** |
| 1a. Study design, end of intervention (Potential confounders taken into account) | Yes | RCT, RDD, ITS, instrumental variable |
| 2. Masking or blinding | No | Unblinded or no mention of blinding |
| 3. Power calculation | Unclear | Medium: No discussion of power calculation |
| 4a. Losses to follow up are presented and acceptable (End of intervention) | No | Attrition not reported, OR falls well outside WWC acceptable combined levels* |
| 4b. Losses to follow up are presented and acceptable (Longest follow up, if applicable) | No | Attrition not reported, OR falls well outside WWC acceptable combined levels* |
| 5. Definition of intervention are clearly defined | Unclear | Medium: Brief description of intervention |
| 6. Outcome measures are clearly defined and reliable | Unclear | Medium: Brief description of outcome |
| 7. Baseline balance (N.A. for before versus after) | Yes | RCT or baseline balance report and satisfactory (imbalance on 2 or less measures) |
| Overall confidence in study (end of intervention) | No | Low confidence: Low on any one of items 1a, 4a, 6 and 7 |
| Overall confidence- short | No | Low confidence |

[Smelson 2018](#STD-Smelson-2018)

| ***Study characteristics*** | | |
| --- | --- | --- |
| Study design | Two-arm parallel RCT (cluster randomised) | |
| Harm reduction or abstinence-based? | HR | |
| Primary intervention | Talking therapies (CBT) + ACT | |
| Longest intervention follow-up (months, N) | 12 months; n = 81 | |
| Control group | TAU | |
| Measure of interest | Drug and alcohol use - dichotomous measure based on case managers’ clinical assessment of Veterans’ drug or alcohol use as either dependence or severe dependence | |
| Source | EGM | |
| Notes |  | |
|  | | |
| **Item** | **Authors' judgement** | **Support for judgement** |
| 1a. Study design, end of intervention (Potential confounders taken into account) | Yes | RCT, RDD, ITS, instrumental variable |
| 2. Masking or blinding | No | Unblinded or no mention of blinding |
| 3. Power calculation | Unclear | Medium: No discussion of power calculation |
| 4a. Losses to follow up are presented and acceptable (End of intervention) | Unclear | Medium: Overall and differential attrition within WWC liberal levels* |
| 5. Definition of intervention are clearly defined | Yes | Intervention clearly and fully described |
| 6. Outcome measures are clearly defined and reliable | Yes | Outcome measure clearly and fully described, preferably with reference to validation |
| 7. Baseline balance (N.A. for before versus after) | Yes | RCT or baseline balance report and satisfactory (imbalance on 2 or less measures) |
| Overall confidence in study (end of intervention) | Unclear | Medium confidence: Medium on any one of items 1a, 4a, 6 and 7 AND NOT LOW |
| Overall confidence- short | Unclear | Medium confidence |

[Smith 1998](#STD-Smith-1998)

| ***Study characteristics*** | | |
| --- | --- | --- |
| Study design | Multi-arm parallel RCT (individually randomised) | |
| Harm reduction or abstinence-based? | AB | |
| Primary intervention | CM | |
| Longest intervention follow-up (months, N) | 12 months; n = 63 | |
| Control group | TAU | |
| Measure of interest | Peak BAC (Blood Alcohol Content) - peak blood alcohol content (BAC) estimated from the steady drinking pattern reported AND days drinking per week | |
| Source | EGM | |
| Notes | Intervention is Community Reinforcement Approach (CRA) - alcohol treatment with incenntives for attendance | |
|  | | |
| **Item** | **Authors' judgement** | **Support for judgement** |
| 1a. Study design, end of intervention (Potential confounders taken into account) | Yes | RCT, RDD, ITS, instrumental variable |
| 2. Masking or blinding | No | Unblinded or no mention of blinding |
| 3. Power calculation | Unclear | Medium: No discussion of power calculation |
| 4a. Losses to follow up are presented and acceptable (End of intervention) | Yes | Overall and differential attrition within WWC conservative levels* |
| 5. Definition of intervention are clearly defined | Yes | Intervention clearly and fully described |
| 6. Outcome measures are clearly defined and reliable | Yes | Outcome measure clearly and fully described, preferably with reference to validation |
| 7. Baseline balance (N.A. for before versus after) | Yes | RCT or baseline balance report and satisfactory (imbalance on 2 or less measures) |
| Overall confidence in study (end of intervention) | Yes | High confidence: High on all of items 1a, 4a, 6 and 7 |
| Overall confidence- short | Yes | High confidence |

[Somers 2015](#STD-Somers-2015)

| ***Study characteristics*** | | |
| --- | --- | --- |
| Study design | Multi-arm parallel RCT (individually randomised) | |
| Harm reduction or abstinence-based? | HR | |
| Primary intervention | ACT or ICM | |
| Longest intervention follow-up (months, N) | Group A (High needs): 24 months; n = 90; Group B (Moderate needs): 24 months; n = 100 | |
| Control group | TAU | |
| Measure of interest | Less than daily substance use - derived using items from the Maudsley Addiction Profile addressing the frequency of specific substance use including alcohol. | |
| Source | EGM | |
| Notes |  | |
|  | | |
| **Item** | **Authors' judgement** | **Support for judgement** |
| 1a. Study design, end of intervention (Potential confounders taken into account) | Yes | RCT, RDD, ITS, instrumental variable |
| 2. Masking or blinding | No | Unblinded or no mention of blinding |
| 3. Power calculation | Unclear | Medium: No discussion of power calculation |
| 4a. Losses to follow up are presented and acceptable (End of intervention) | Unclear | Medium: Overall and differential attrition within WWC liberal levels* |
| 5. Definition of intervention are clearly defined | Yes | Intervention clearly and fully described |
| 6. Outcome measures are clearly defined and reliable | Yes | Outcome measure clearly and fully described, preferably with reference to validation |
| 7. Baseline balance (N.A. for before versus after) | Yes | RCT or baseline balance report and satisfactory (imbalance on 2 or less measures) |
| Overall confidence in study (end of intervention) | Unclear | Medium confidence: Medium on any one of items 1a, 4a, 6 and 7 AND NOT LOW |
| Overall confidence- short | Unclear | Medium confidence |

[Somers 2017](#STD-Somers-2017)

| ***Study characteristics*** | | |
| --- | --- | --- |
| Study design | Two-arm parallel RCT (individually randomised) | |
| Harm reduction or abstinence-based? | HR | |
| Primary intervention | Assertive Community Therapy (ACT) - Group A: Congregate Housing First. Group B:Scattered site Housing First | |
| Longest intervention follow-up (months, N) | Group A: 24 months; n = 107; Group B: 24 months; n = 90 | |
| Control group | TAU | |
| Measure of interest | Substance use problem past month (GAIN-SPS) | |
| Source | EGM | |
| Notes |  | |
|  | | |
| **Item** | **Authors' judgement** | **Support for judgement** |
| 1a. Study design, end of intervention (Potential confounders taken into account) | Yes | RCT, RDD, ITS, instrumental variable |
| 2. Masking or blinding | No | Unblinded or no mention of blinding |
| 3. Power calculation | Unclear | Medium: No discussion of power calculation |
| 4a. Losses to follow up are presented and acceptable (End of intervention) | Unclear | Medium: Overall and differential attrition within WWC liberal levels* |
| 5. Definition of intervention are clearly defined | Yes | Intervention clearly and fully described |
| 6. Outcome measures are clearly defined and reliable | Yes | Outcome measure clearly and fully described, preferably with reference to validation |
| 7. Baseline balance (N.A. for before versus after) | Yes | RCT or baseline balance report and satisfactory (imbalance on 2 or less measures) |
| Overall confidence in study (end of intervention) | Unclear | Medium confidence: Medium on any one of items 1a, 4a, 6 and 7 AND NOT LOW |
| Overall confidence- short | Unclear | Medium confidence |

[Sosin 1995](#STD-Sosin-1995)

| ***Study characteristics*** | | |
| --- | --- | --- |
| Study design | Multi-arm parallel RCT (individually randomised) | |
| Harm reduction or abstinence-based? | AB | |
| Primary intervention | ICM and ICM + supported housing | |
| Longest intervention follow-up (months, N) | Group A: 12 months; n = 72; Group B: 12 months; n = 111 | |
| Control group | TAU | |
| Measure of interest | Average Days Using Alcohol/Drugs in Past 30 | |
| Source | EGM | |
| Notes | Authors list intervention as: Group A: Case management only intervention . Group B: Case management with supported housing intervention. | |
|  | | |
| **Item** | **Authors' judgement** | **Support for judgement** |
| 1a. Study design, end of intervention (Potential confounders taken into account) | Yes | RCT, RDD, ITS, instrumental variable |
| 1b. Study design, longest follow up if applicable (Potential confounders taken into account) | Yes | RCT, RDD, ITS, instrumental variable |
| 2. Masking or blinding | No | Unblinded or no mention of blinding |
| 3. Power calculation | Yes | Mention of power calculation |
| 4a. Losses to follow up are presented and acceptable (End of intervention) | Yes | Overall and differential attrition within WWC conservative levels* |
| 4b. Losses to follow up are presented and acceptable (Longest follow up, if applicable) | Yes | Overall and differential attrition within WWC conservative levels* |
| 5. Definition of intervention are clearly defined | Yes | Intervention clearly and fully described |
| 6. Outcome measures are clearly defined and reliable | Yes | Outcome measure clearly and fully described, preferably with reference to validation |
| 7. Baseline balance (N.A. for before versus after) | Yes | RCT or baseline balance report and satisfactory (imbalance on 2 or less measures) |
| Overall confidence in study (end of intervention) | Yes | High on all of items 1a, 4a, 6 and 7 |
| Overall confidence- short | Yes | High confidence |

[Stahler 1995](#STD-Stahler-1995)

| ***Study characteristics*** | | |
| --- | --- | --- |
| Study design | Multi-arm parallel RCT (individually randomised) | |
| Harm reduction or abstinence-based? | AB | |
| Primary intervention | Group A: Residential Rehabilitation. Group B: ICM | |
| Longest intervention follow-up (months, N) | Group A: 12 months; n = 187 ; Group B: 12 months; n = 170 | |
| Control group | TAU | |
| Measure of interest | ASI Cocaine AND ASI alcohol | |
| Source | Unpacked from SR | |
| Notes | Treatment groups: 85% retention rate - rounded up for analysis; Control group: 69% retention rate - rounded up for analysis | |
|  | | |
| **Item** | **Authors' judgement** | **Support for judgement** |
| 1a. Study design, end of intervention (Potential confounders taken into account) | Yes | RCT, RDD, ITS, instrumental variable |
| 1b. Study design, longest follow up if applicable (Potential confounders taken into account) | Yes | RCT, RDD, ITS, instrumental variable |
| 2. Masking or blinding | No | Unblinded or no mention of blinding |
| 3. Power calculation | Unclear | No discussion of power calculation |
| 4a. Losses to follow up are presented and acceptable (End of intervention) | Yes | Overall and differential attrition within WWC conservative levels* |
| 4b. Losses to follow up are presented and acceptable (Longest follow up, if applicable) | Yes | Overall and differential attrition within WWC conservative levels* |
| 5. Definition of intervention are clearly defined | Yes | Intervention clearly and fully described |
| 6. Outcome measures are clearly defined and reliable | Yes | Outcome measure clearly and fully described, preferably with reference to validation |
| 7. Baseline balance (N.A. for before versus after) | Yes | RCT or baseline balance report and satisfactory (imbalance on 2 or less measures) |
| Overall confidence in study (end of intervention) | Yes | High confidence: High on all of items 1a, 4a, 6 and 7 |
| Overall confidence- short | Yes | High confidence |

[Stahler 2005](#STD-Stahler-2005)

| ***Study characteristics*** | | |
| --- | --- | --- |
| Study design | QED - other | |
| Harm reduction or abstinence-based? | AB | |
| Primary intervention | TC | |
| Longest intervention follow-up (months, N) | 18 months; n = 40 | |
| Control group | TAU | |
| Measure of interest | Addiction Severity Index - mean days of reported substance use in previous 30 days for cocaine AND alcohol AND more than one substance | |
| Source | Unpacked from SR | |
| Notes |  | |
|  | | |
| **Item** | **Authors' judgement** | **Support for judgement** |
| 1a. Study design, end of intervention (Potential confounders taken into account) | Unclear | Medium confidence: DiD with matching, PSM |
| 2. Masking or blinding | No | Unblinded or no mention of blinding |
| 3. Power calculation | Unclear | Medium: No discussion of power calculation |
| 4a. Losses to follow up are presented and acceptable (End of intervention) | Unclear | Medium: Overall and differential attrition within WWC liberal levels* |
| 5. Definition of intervention are clearly defined | Yes | Intervention clearly and fully described |
| 6. Outcome measures are clearly defined and reliable | Yes | Outcome measure clearly and fully described, preferably with reference to validation |
| 7. Baseline balance (N.A. for before versus after) | Yes | RCT or baseline balance report and satisfactory (imbalance on 2 or less measures) |
| Overall confidence in study (end of intervention) | Unclear | Medium confidence: Medium on any one of items 1a, 4a, 6 and 7 AND NOT LOW |
| Overall confidence- short | Unclear | Medium confidence |

[Stergiopoulos 2015](#STD-Stergiopoulos-2015)

| ***Study characteristics*** | | |
| --- | --- | --- |
| Study design | Two-arm parallel RCT (individually randomised) | |
| Harm reduction or abstinence-based? | HR | |
| Primary intervention | ICM | |
| Longest intervention follow-up (months, N) | 24 months; n = 204 | |
| Control group | TAU | |
| Measure of interest | Substance Use Problem Severity (GAIN-SS) | |
| Source | EGM | |
| Notes |  | |
|  | | |
| **Item** | **Authors' judgement** | **Support for judgement** |
| 1a. Study design, end of intervention (Potential confounders taken into account) | Yes | RCT, RDD, ITS, instrumental variable |
| 2. Masking or blinding | No | Unblinded or no mention of blinding |
| 3. Power calculation | Yes | Mention of power calculation |
| 4a. Losses to follow up are presented and acceptable (End of intervention) | Yes | Overall and differential attrition within WWC conservative levels* |
| 5. Definition of intervention are clearly defined | Yes | Intervention clearly and fully described |
| 6. Outcome measures are clearly defined and reliable | Yes | Outcome measure clearly and fully described, preferably with reference to validation |
| 7. Baseline balance (N.A. for before versus after) | Yes | RCT or baseline balance report and satisfactory (imbalance on 2 or less measures) |
| Overall confidence in study (end of intervention) | Yes | High confidence: High on all of items 1a, 4a, 6 and 7 |
| Overall confidence- short | Yes | High confidence |

[Stergiopoulos 2016](#STD-Stergiopoulos-2016)

| ***Study characteristics*** | | |
| --- | --- | --- |
| Study design | Two-arm parallel RCT (individually randomised) | |
| Harm reduction or abstinence-based? | HR | |
| Primary intervention | ICM | |
| Longest intervention follow-up (months, N) | 24 months; n = 135 | |
| Control group | TAU | |
| Measure of interest | Substance Use Problem Severity (GAIN-SS) AND Number of days in past 30 experienced alcohol problems | |
| Source | EGM | |
| Notes |  | |
|  | | |
| **Item** | **Authors' judgement** | **Support for judgement** |
| 1a. Study design, end of intervention (Potential confounders taken into account) | Yes | RCT, RDD, ITS, instrumental variable |
| 1b. Study design, longest follow up if applicable (Potential confounders taken into account) | Yes | RCT, RDD, ITS, instrumental variable |
| 2. Masking or blinding | No | Unblinded or no mention of blinding |
| 3. Power calculation | Yes | Mention of power calculation |
| 4a. Losses to follow up are presented and acceptable (End of intervention) | No | Attrition not reported, OR falls well outside WWC acceptable combined levels* |
| 5. Definition of intervention are clearly defined | Yes | Intervention clearly and fully described |
| 6. Outcome measures are clearly defined and reliable | Yes | Outcome measure clearly and fully described, preferably with reference to validation |
| 7. Baseline balance (N.A. for before versus after) | Yes | High: RCT or baseline balance report and satisfactory (imbalance on 2 or less measures) |
| Overall confidence in study (end of intervention) | No | Low confidence: Low on any one of items 1a, 4a, 6 and 7 |
| Overall confidence- short | No | Low confidence |

[Stergiopoulos 2019](#STD-Stergiopoulos-2019)

| ***Study characteristics*** | | |
| --- | --- | --- |
| Study design | Two-arm parallel RCT (individually randomised) | |
| Harm reduction or abstinence-based? | HR | |
| Primary intervention | ACT or ICM | |
| Longest intervention follow-up (months, N) | 72 months; n = 206 | |
| Control group | TAU | |
| Measure of interest | GAIN-SS number of substance use-related problems over past month - the five-item Global Appraisal of Individual Needs–Short Screener (GAIN-SS) - with higher values denoting higher severity | |
| Source | EGM | |
| Notes |  | |
|  | | |
| **Item** | **Authors' judgement** | **Support for judgement** |
| 1a. Study design, end of intervention (Potential confounders taken into account) | Yes | RCT, RDD, ITS, instrumental variable |
| 2. Masking or blinding | No | Unblinded or no mention of blinding |
| 3. Power calculation | Unclear | Medium: No discussion of power calculation |
| 4a. Losses to follow up are presented and acceptable (End of intervention) | Unclear | Medium: Overall and differential attrition within WWC liberal levels* |
| 5. Definition of intervention are clearly defined | Yes | Intervention clearly and fully described |
| 6. Outcome measures are clearly defined and reliable | Yes | Outcome measure clearly and fully described, preferably with reference to validation |
| 7. Baseline balance (N.A. for before versus after) | Yes | RCT or baseline balance report and satisfactory (imbalance on 2 or less measures) |
| Overall confidence in study (end of intervention) | Unclear | Medium confidence: Medium on any one of items 1a, 4a, 6 and 7 AND NOT LOW |
| Overall confidence- short | Unclear | Medium confidence |

[Thompson 2020](#STD-Thompson-2020)

| ***Study characteristics*** | | |
| --- | --- | --- |
| Study design | Two-arm parallel RCT (individually randomised) | |
| Harm reduction or abstinence-based? | HR | |
| Primary intervention | MI | |
| Longest intervention follow-up (months, N) | 1.5 months, n=20 | |
| Control group | TAU | |
| Measure of interest | Number of drinks consumed in last two weeks. Times used marijuana in last two weeks | |
| Source | EGM | |
| Notes |  | |
|  | | |
| **Item** | **Authors' judgement** | **Support for judgement** |
| 1a. Study design, end of intervention (Potential confounders taken into account) | Yes | RCT, RDD, ITS, instrumental variable |
| 2. Masking or blinding | No | Unblinded or no mention of blinding |
| 3. Power calculation | Unclear | Medium: No discussion of power calculation |
| 4a. Losses to follow up are presented and acceptable (End of intervention) | Unclear | Medium: Overall and differential attrition within WWC liberal levels* |
| 5. Definition of intervention are clearly defined | Yes | Intervention clearly and fully described |
| 6. Outcome measures are clearly defined and reliable | Yes | Outcome measure clearly and fully described, preferably with reference to validation |
| 7. Baseline balance (N.A. for before versus after) | Yes | RCT or baseline balance report and satisfactory (imbalance on 2 or less measures) |
| Overall confidence in study (end of intervention) | Unclear | Medium confidence: Medium on any one of items 1a, 4a, 6 and 7 AND NOT LOW |
| Overall confidence- short | Unclear | Medium confidence |

[Tinland 2020](#STD-Tinland-2020)

| ***Study characteristics*** | | |
| --- | --- | --- |
| Study design | Two-arm parallel RCT (individually randomised) | |
| Harm reduction or abstinence-based? | HR | |
| Primary intervention | ACT | |
| Longest intervention follow-up (months, N) | 24 months; n = 353 | |
| Control group | TAU | |
| Measure of interest | Presence or absence of diagnoses of dependence - Alcohol - using sections K and J of the Mini International Neuropsychiatric Interview (MINI) | |
| Source | EGM | |
| Notes |  | |
|  | | |
| **Item** | **Authors' judgement** | **Support for judgement** |
| 1a. Study design, end of intervention (Potential confounders taken into account) | Yes | RCT, RDD, ITS, instrumental variable |
| 2. Masking or blinding | No | Unblinded or no mention of blinding |
| 3. Power calculation | Unclear | Medium: No discussion of power calculation |
| 4a. Losses to follow up are presented and acceptable (End of intervention) | Yes | Overall and differential attrition within WWC conservative levels* |
| 5. Definition of intervention are clearly defined | Yes | Intervention clearly and fully described |
| 6. Outcome measures are clearly defined and reliable | Yes | Outcome measure clearly and fully described, preferably with reference to validation |
| 7. Baseline balance (N.A. for before versus after) | Yes | RCT or baseline balance report and satisfactory (imbalance on 2 or less measures) |
| Overall confidence in study (end of intervention) | Yes | High confidence: High on all of items 1a, 4a, 6 and 7 |
| Overall confidence- short | Yes | High confidence |

[Tsemberis 2004](#STD-Tsemberis-2004)

| ***Study characteristics*** | | |
| --- | --- | --- |
| Study design | Two-arm parallel RCT (individually randomised) | |
| Harm reduction or abstinence-based? | HR | |
| Primary intervention | ACT | |
| Longest intervention follow-up (months, N) | 24 months; n = 68 | |
| Control group | Other intervention - Continuum of Care model | |
| Measure of interest | Number of drinks consumed each day AND Drug use variable (measuring the total number of days of drug use) | |
| Source | Unpacked from SR | |
| Notes | Authors report 78% follow-up rate - rounded up for effect size calcs | |
|  | | |
| **Item** | **Authors' judgement** | **Support for judgement** |
| 1a. Study design, end of intervention (Potential confounders taken into account) | Yes | RCT, RDD, ITS, instrumental variable |
| 2. Masking or blinding | Yes | EITHER masking of of outcome measurement OR masking for analysis (or both) |
| 3. Power calculation | Yes | Mention of power calculation |
| 4a. Losses to follow up are presented and acceptable (End of intervention) | Yes | Overall and differential attrition within WWC conservative levels* |
| 5. Definition of intervention are clearly defined | Yes | Intervention clearly and fully described |
| 6. Outcome measures are clearly defined and reliable | Yes | Outcome measure clearly and fully described, preferably with reference to validation |
| 7. Baseline balance (N.A. for before versus after) | Yes | RCT or baseline balance report and satisfactory (imbalance on 2 or less measures) |
| Overall confidence in study (end of intervention) | Yes | High confidence: High on all of items 1a, 4a, 6 and 7 |
| Overall confidence- short | Yes | High confidence |

[Tucker 2017](#STD-Tucker-2017)

| ***Study characteristics*** | | |
| --- | --- | --- |
| Study design | Cross-over RCT | |
| Harm reduction or abstinence-based? | HR | |
| Primary intervention | MI | |
| Longest intervention follow-up (months, N) | 3 months; n = 100 | |
| Control group | TAU | |
| Measure of interest | Frequency of consumption - Alcohol AND Other drugs | |
| Source | Hand search | |
| Notes |  | |
|  | | |
| **Item** | **Authors' judgement** | **Support for judgement** |
| 1a. Study design, end of intervention (Potential confounders taken into account) | Yes | RCT, RDD, ITS, instrumental variable |
| 2. Masking or blinding | No | Unblinded or no mention of blinding |
| 3. Power calculation | Yes | Mention of power calculation |
| 4a. Losses to follow up are presented and acceptable (End of intervention) | No | Attrition not reported, OR falls well outside WWC acceptable combined levels* |
| 5. Definition of intervention are clearly defined | Yes | Intervention clearly and fully described |
| 6. Outcome measures are clearly defined and reliable | Yes | Outcome measure clearly and fully described, preferably with reference to validation |
| 7. Baseline balance (N.A. for before versus after) | Yes | RCT or baseline balance report and satisfactory (imbalance on 2 or less measures) |
| Overall confidence in study (end of intervention) | No | Low confidence: Low on any one of items 1a, 4a, 6 and 7 |
| Overall confidence- short | No | Low confidence |

[Upshur 2015](#STD-Upshur-2015)

| ***Study characteristics*** | | |
| --- | --- | --- |
| Study design | Two-arm parallel RCT (individually randomised) | |
| Harm reduction or abstinence-based? | HR | |
| Primary intervention | ICM + MI | |
| Longest intervention follow-up (months, N) | 6 months; n = 40 | |
| Control group | TAU | |
| Measure of interest | Days used other drugs last 3 months AND No alcohol consumption last 3 months (abstinence) | |
| Source | EGM | |
| Notes |  | |
|  | | |
| **Item** | **Authors' judgement** | **Support for judgement** |
| 1a. Study design, end of intervention (Potential confounders taken into account) | Yes | RCT, RDD, ITS, instrumental variable |
| 2. Masking or blinding | No | Unblinded or no mention of blinding |
| 3. Power calculation | Unclear | Medium: No discussion of power calculation |
| 4a. Losses to follow up are presented and acceptable (End of intervention) | Yes | Overall and differential attrition within WWC conservative levels* |
| 5. Definition of intervention are clearly defined | Yes | Intervention clearly and fully described |
| 6. Outcome measures are clearly defined and reliable | Yes | Outcome measure clearly and fully described, preferably with reference to validation |
| 7. Baseline balance (N.A. for before versus after) | Yes | RCT or baseline balance report and satisfactory (imbalance on 2 or less measures) |
| Overall confidence in study (end of intervention) | Yes | High confidence: High on all of items 1a, 4a, 6 and 7 |
| Overall confidence- short | Yes | High confidence |

[Young 2009](#STD-Young-2009)

| ***Study characteristics*** | | |
| --- | --- | --- |
| Study design | QED - DID | |
| Harm reduction or abstinence-based? | HR | |
| Primary intervention | ACT + residential rehabilitation | |
| Longest intervention follow-up (months, N) | 6 months; n = 53 | |
| Control group | Other intervention - Substance abuse treatment agency and used the CCISC model of care in a residential treatment facility (CCISC-RT) | |
| Measure of interest | Days past-month using alcohol AND Days past-month using illicit drugs AND Abstinent past month Alcohol AND Abstinent past month drugs | |
| Source | Unpacked from SR | |
| Notes |  | |
|  | | |
| **Item** | **Authors' judgement** | **Support for judgement** |
| 1a. Study design, end of intervention (Potential confounders taken into account) | Unclear | Medium confidence: DiD with matching, PSM |
| 1b. Study design, longest follow up if applicable (Potential confounders taken into account) | Unclear | Medium confidence: DiD with matching, PSM |
| 2. Masking or blinding | No | Unblinded or no mention of blinding |
| 3. Power calculation | Unclear | No discussion of power calculation |
| 4a. Losses to follow up are presented and acceptable (End of intervention) | No | Attrition not reported, OR falls well outside WWC acceptable combined levels* |
| 4b. Losses to follow up are presented and acceptable (Longest follow up, if applicable) | No | Attrition not reported, OR falls well outside WWC acceptable combined levels |
| 5. Definition of intervention are clearly defined | Yes | Intervention clearly and fully described |
| 6. Outcome measures are clearly defined and reliable | Yes | Outcome measure clearly and fully described, preferably with reference to validation |
| 7. Baseline balance (N.A. for before versus after) | Unclear | Medium confidence: imbalance on no more than 5 measures |
| Overall confidence in study (end of intervention) | No | Low confidence: Low on any one of items 1a, 4a, 6 and 7 |
| Overall confidence- short | No | Low confidence |

Characteristics of excluded studies [ordered by study ID]

| Study | Reason for exclusion |
| --- | --- |
| [Aldridge 2017](#STD-Aldridge-2017) | Does not measure relevant outcomes |
| [Aldridge 2019](#STD-Aldridge-2019) | Not a relevant intervention |
| [Alunni-Menichini 2020](#STD-Alunni_x002d_Menichini-2020) | Research design |
| [Appel 2012](#STD-Appel-2012) | Research design |
| [Argeriou 1993](#STD-Argeriou-1993) | Research design |
| [Aubry 2015](#STD-Aubry-2015) | Does not measure relevant outcomes |
| [Aubry 2020](#STD-Aubry-2020) | Participants do not meet age criteria |
| [Baer 2007](#STD-Baer-2007) | Participants do not meet age criteria |
| [Ballard 2002](#STD-Ballard-2002) | Researh design |
| [Barrow 1999](#STD-Barrow-1999) | Research design |
| [Bartle-Haring 2012](#STD-Bartle_x002d_Haring-2012) | Research design |
| [Basu 2012](#STD-Basu-2012) | Does not measure relevant outcomes |
| [Beieler 2019](#STD-Beieler-2019) | Does not measure relevant outcomes |
| [Bell 2015](#STD-Bell-2015) | Does not measure relevant outcomes |
| [Bernad 2016](#STD-Bernad-2016) | Not a relevant intervention |
| [Bradford 2005](#STD-Bradford-2005) | Does not measure relevant outcomes |
| [Brown 2010](#STD-Brown-2010) | Does not measure relevant outcomes |
| [Brown 2018](#STD-Brown-2018) | Research design |
| [Caton 2000](#STD-Caton-2000) | Research design |
| [Cauce 1994](#STD-Cauce-1994) | Participants do not meet age criteria |
| [Cheng 2007](#STD-Cheng-2007) | Not a relevant intervention |
| [Chu 2020](#STD-Chu-2020) | Does not measure relevant outcomes |
| [Chum 2020](#STD-Chum-2020) | Not a relevant intervention |
| [Chung 2018a](#STD-Chung-2018a) | Not a relevant intervention |
| [Clark 2003](#STD-Clark-2003) | Not a relevant intervention |
| [Collins 2020](#STD-Collins-2020) | Research design |
| [Collins 2021a](#STD-Collins-2021a) | Does not measure relevant outcomes |
| [Consoli 2016](#STD-Consoli-2016) | Research design |
| [Cox 1998](#STD-Cox-1998) | Not a relevant intervention |
| [Cross 2018](#STD-Cross-2018) | Research design |
| [Daubresse 2017](#STD-Daubresse-2017) | Does not measure relevant outcomes |
| [de Vet 2017](#STD-de-Vet-2017) | Not a relevant intervention |
| [Dionisi 2020](#STD-Dionisi-2020) | Research design |
| [Drake 1998](#STD-Drake-1998) | Does not measure relevant outcome |
| [Ellison 2020](#STD-Ellison-2020) | Not a relevant intervention |
| [Fletcher 2013](#STD-Fletcher-2013) | Research design |
| [Fors 1995](#STD-Fors-1995) | Participants do not meet age criteria |
| [Frisman 2009](#STD-Frisman-2009) | Does not measure relevant outcomes |
| [Gesmond 2017](#STD-Gesmond-2017) | Not a relevant intervention |
| [Grelottia 2017](#STD-Grelottia-2017) | Not a relevant intervention |
| [Gulcur 2003](#STD-Gulcur-2003) | Does not measure relevant outcomes |
| [Hall 2020](#STD-Hall-2020) | Not a relevant intervention |
| [Hatcher 2018](#STD-Hatcher-2018) | Study not complete/protocol only |
| [Hickman 2018](#STD-Hickman-2018) | Research design |
| [Hurlburt 1996](#STD-Hurlburt-1996) | Does not measure relevant outcomes |
| [Hwang 2011](#STD-Hwang-2011) | Not a relevant intervention |
| [Jarvis 2017](#STD-Jarvis-2017) | Does not measure relevant outcomes |
| [Jones 2018](#STD-Jones-2018) | Research design |
| [Karper 2008](#STD-Karper-2008) | Not a relevant intervention |
| [Kasprow 2007](#STD-Kasprow-2007) | Research design |
| [Kennedy 2016](#STD-Kennedy-2016) | Study not complete/protocol only |
| [Kerman 2020](#STD-Kerman-2020) | Not a relevant intervention |
| [Kertesz 2005](#STD-Kertesz-2005) | Does not measure relevant outcomes |
| [Kirby 1999](#STD-Kirby-1999) | Not a relevant intervention |
| [Kirst 2015a](#STD-Kirst-2015a) | Not a relevant intervention |
| [Kisely 2008](#STD-Kisely-2008) | Not a relevant intervention |
| [Korr 1995](#STD-Korr-1995) | Not a relevant intervention |
| [Kozloff 2016](#STD-Kozloff-2016) | Does not measure relevant outcomes |
| [Kozloff 2016a](#STD-Kozloff-2016a) | Does not measure relevant outcomes |
| [Krawczyk 2020](#STD-Krawczyk-2020) | Does not measure relevant outcomes |
| [Kwiatkowski 2000](#STD-Kwiatkowski-2000) | Does not measure relevant outcomes |
| [Lachaud 2021](#STD-Lachaud-2021) | Not a relevant intervention |
| [Larance 2020](#STD-Larance-2020) | Research design |
| [Larimer 2009](#STD-Larimer-2009) | Research design |
| [Latimer 2017](#STD-Latimer-2017) | Not a relevant intervention |
| [Latimer 2020](#STD-Latimer-2020) | Does not measure relevant outcomes |
| [Leaf 1993](#STD-Leaf-1993) | Study not complete/protocol only |
| [Lemoine 2019](#STD-Lemoine-2019) | Not a relevant intervention |
| [LePage 2012](#STD-LePage-2012) | Research design |
| [Lester 2007](#STD-Lester-2007) | Does not measure relevant outcomes |
| [Lim 2018](#STD-Lim-2018) | Not a relevant intervention |
| [Lin 2021](#STD-Lin-2021) | Does not measure relevant outcomes |
| [Lipton 1988](#STD-Lipton-1988) | Published prior to 1990 |
| [Lipton 2000](#STD-Lipton-2000) | Does not measure relevant outcomes |
| [Lowrie 2021](#STD-Lowrie-2021) | Not a relevant intervention |
| [Luong 2021](#STD-Luong-2021) | Not a relevant intervention |
| [Malte 2017](#STD-Malte-2017) | Not a relevant intervention |
| [Mares 2011](#STD-Mares-2011) | Not a relevant intervention |
| [McGlave 2017](#STD-McGlave-2017) | Does not measure relevant outcomes |
| [McGuire 2009](#STD-McGuire-2009) | Does not measure relevant outcomes |
| [Meacham 2018](#STD-Meacham-2018) | Does not measure relevant outcomes |
| [Milby 2005](#STD-Milby-2005) | Not a relevant intervention |
| [Milby 2010](#STD-Milby-2010) | Not a relevant intervention |
| [Moore 2015](#STD-Moore-2015) | Research design |
| [Morse 1997a](#STD-Morse-1997a) | Not a relevant intervention |
| [Morse 2020](#STD-Morse-2020) | Research design |
| [Nelson 2012](#STD-Nelson-2012) | Research design |
| [Nuttbrock 1998](#STD-Nuttbrock-1998) | Research design |
| [Nyamathi 2001](#STD-Nyamathi-2001) | Does not measure relevant outcomes |
| [Nyamathi 2012](#STD-Nyamathi-2012) | Not a relevant intervention |
| [Nyamathi 2016a](#STD-Nyamathi-2016a) | Does not measure relevant outcomes |
| [O'Campo 2016](#STD-O_x0027_Campo-2016) | Does not measure relevant outcomes |
| [O'Connell 2009](#STD-O_x0027_Connell-2009) | Does not measure relevant outcomes |
| [O'Toole 2018](#STD-O_x0027_Toole-2018) | Does not measure relevant outcomes |
| [Orwin 1994](#STD-Orwin-1994) | Not a relevant intervention |
| [Osypuk 2019](#STD-Osypuk-2019) | Does not measure relevant outcomes |
| [Padgett 2006](#STD-Padgett-2006) | Not a relevant intervention |
| [Padgett 2011](#STD-Padgett-2011) | Not a relevant intervention |
| [Parker 2010](#STD-Parker-2010) | Research design |
| [Parpouchi 2018](#STD-Parpouchi-2018) | Not a relevant intervention |
| [Pauly 2019](#STD-Pauly-2019) | Not a relevant intervention |
| [Pearson 2009](#STD-Pearson-2009) | Does not measure relevant outcomes |
| [Pennington 2018](#STD-Pennington-2018) | Research design |
| [Pietrusza 2018](#STD-Pietrusza-2018) | Research design |
| [Poremski 2015](#STD-Poremski-2015) | Does not measure relevant outcomes |
| [Rash 2017](#STD-Rash-2017) | Population not just adults experiencing homelessness |
| [Resnick 2008](#STD-Resnick-2008) | Does not measure relevant outcomes |
| [Rew 2017](#STD-Rew-2017) | Not a relevant intervention |
| [Rich 2005](#STD-Rich-2005) | Not a relevant intervention |
| [Rosenblum 2002](#STD-Rosenblum-2002) | Not a relevant intervention |
| [Rosenheck 2007](#STD-Rosenheck-2007) | Does not measure relevant outcomes |
| [Rotheram-Borus 2003](#STD-Rotheram_x002d_Borus-2003) | Participants do not meet age criteria |
| [Sacks 2004](#STD-Sacks-2004) | Homeless families - not relevant population |
| [Sajatovic 2013](#STD-Sajatovic-2013) | Research design |
| [Santa 2019](#STD-Santa-2019) | Research design |
| [Saxon 2006](#STD-Saxon-2006) | Does not measure relevant outcomes |
| [Schumacher 2002](#STD-Schumacher-2002) | Research design |
| [Schumacher 2007](#STD-Schumacher-2007) | Does not measure relevant outcomes |
| [Shumway 2008](#STD-Shumway-2008) | Not a relevant intervention |
| [Slesnick 2005](#STD-Slesnick-2005) | Participants do not meet age criteria |
| [Slesnick 2007](#STD-Slesnick-2007) | Participants do not meet age criteria |
| [Slesnick 2008](#STD-Slesnick-2008) | Research design |
| [Slesnick 2013](#STD-Slesnick-2013) | Homeless families - not a relevant population |
| [Slesnick 2015](#STD-Slesnick-2015) | Participants do not meet age criteria |
| [Smelson 2018a](#STD-Smelson-2018a) | Research design |
| [Srebnik 2013](#STD-Srebnik-2013) | Not a relevant intervention |
| [Stefancic 2007](#STD-Stefancic-2007) | Research design |
| [Stein 2020](#STD-Stein-2020) | Does not measure relevant outcomes |
| [Stergiopoulos 2018](#STD-Stergiopoulos-2018) | Not a relevant intervention |
| [Stockwell 2013](#STD-Stockwell-2013) | Not a relevant intervention |
| [Toro 1997](#STD-Toro-1997) | Not a relevant intervention |
| [Tsai 2010](#STD-Tsai-2010) | Not a relevant intervention |
| [Tsemberis 2012](#STD-Tsemberis-2012) | Research design |
| [Tucker 2020](#STD-Tucker-2020) | Study not complete/protocol only |
| [Tucker 2021](#STD-Tucker-2021) | Study not complete/protocol only |
| [Urbanoski 2018](#STD-Urbanoski-2018) | Not a relevant intervention |
| [van Kranenburg 2019](#STD-van-Kranenburg-2019) | Does not measure relevant outcomes |
| [van Vugt 2012](#STD-van-Vugt-2012) | Does not measure relevant outcomes |
| [Winn 2013](#STD-Winn-2013) | Does not measure relevant outcomes |
| [Wolitski 2010](#STD-Wolitski-2010) | Does not measure relevant outcomes |
| [Zhang 2018](#STD-Zhang-2018) | Participants did not meet age criteria |
| [Zhang 2018a](#STD-Zhang-2018a) | Not a relevant intervention |

Appendices

Appendix 1. Definitions of substance use interventions set out in Table 1: Typology of interventions

**Abstinence based day programmes** are typically structured day treatment programmes for people with drug and/or alcohol problems. They may offer a range of abstinence-based treatment options which integrate 12-Step Recovery Programmes, Cognitive Behavioural Therapy (CBT) and other appropriate therapies. They may also include personally rewarding structured training, counselling and self-help.

**Agonist pharmacotherapy /blockers** are drugs that bind to and activate receptors. Agonist treatments currently include buprenorphine and methadone (which is the mainstay of UK agonist treatment services), although in some countries or localities prescribed heroin, dihydrocodeine and LAAM (levo-methadol) are used.

**Assertive Community Therapy (ACT)** was originally developed for patients with severe mental illness, providing personalized, high intensity, holistic and integrated multidisciplinary community care services. In its early versions, ACT did not include substance use treatment, but over the past two decades or so most ACT provision has developed to include substance use counselling. ACT can include substance use interventions that are abstinence or harm reduction based, although the latter is more common. Unless otherwise stated, we have assumed ACT intervention studies take a harm reduction approach.

**Assertive Outreach** models recognise that for some drug and alcohol users, their chaotic lifestyle and motivation may lead to non-engagement or missed appointments. Drug and alcohol assertive outreach services aim to increase engagement and improve re-engagement of clients dropping out treatment services. Assertive outreach services will work with clients in a variety of settings to ensure maximum engagement and re-engagement with drug and alcohol treatment services. This may include on the streets or in day centres, hostels and supported accommodation.

**Behaviour Couples Therapy (BCT)** is a form of behaviour therapy originally developed to treat depression, that addresses elements of the couple’s relationship that are known to have a direct effect on the incidence of depression. A key component of this therapy is to focus on improving communication difficulties within the relationship. BCT will aim to specifically improve the overall quality of the relationship.

**Cognitive-Behavioural Therapy (CBT)** is a therapeutic approach that seeks to modify negative or self-defeating thoughts and behaviour. CBT is aimed at both thought and behaviour change—that is, coping by thinking differently and coping by acting differently. Cognitive behavioural therapy is a form of psychotherapy that teaches people strategies to identify and correct problematic associations among thoughts, emotions, and behaviours to enhance self-control and reduce drug use.

**Contingency Management (CM)** is an approach to treatment that maintains that the form or frequency of behaviour can be altered through a planned and organized system of positive and negative consequences. This typically includes regular testing and requirements for treatment engagement. CM assumes that neurobiological and environmental factors influence substance use behaviours and that the consistent application of reinforcing environmental consequences can change these behaviours.

**Drug Consumption Rooms (DCRs)** (also known as safe injecting sites or overdose prevention centres) allow people to take drugs in clean, clinically supervised spaces, where they can also access advice and support. In some cases, these also facilitate non-injecting drug use, which have been shown to reduce overdoses and transmission of HIV.

**Detoxification** involves a clearing of toxins from the body. In substance use treatment, it is a term used to describe the medical and biopsychosocial procedure that assists a person who is dependent on one or more substances to withdraw from dependence on all substances of abuse.

**Eye Movement Desensitization and Reprocessing (EMDR)** is a psychotherapy treatment that was originally designed to alleviate the distress associated with traumatic memories. EMDR therapy facilitates the accessing and processing of traumatic memories and other adverse life experience to bring these to an adaptive resolution. During EMDR therapy the client attends to emotionally disturbing material in brief sequential doses while simultaneously focusing on an external stimulus. Therapist directed lateral eye movements are the most commonly used external stimulus but a variety of other stimuli including hand-tapping and audio stimulation are often used.

**Group work** in the context of substance use treatment can involve a variety of group treatment models to meet client needs during the multiphase process of recovery. This may include skills development groups, cognitive–behavioural/problem solving groups, or support groups.

**Harm reduction-based day centres** provide support to help adults reduce the harm drugs and alcohol can cause and often provide outreach and in-reach support.

**Harm reduction psychotherapy** draws on the concept of harm reduction within a psychotherapeutic approach that integrates cognitive and behavioural interventions with a psychodynamic understanding of substance use as personally meaningful.

**Heroin Assisted Therapy (HAT)** is the prescription of heroin (diamorphine). It is typically used for people who do not respond to OST. This treatment has been shown to improve health outcomes in some of the most dependent of those people who inject drugs.

**Motivational Enhancement Therapy (MET)** is a counselling approach that helps individuals resolve their ambivalence about engaging in treatment and stopping their drug use. This approach aims to evoke rapid and internally motivated change, rather than guide the patient stepwise through the recovery process.

**Motivational Interviewing (MI)** is a client-centred, directive method for enhancing intrinsic motivation to change by exploring and resolving ambivalence.

**Naloxone** is widely used as an effective opioid overdose reversal agent. It is an opioid antagonist that effectively blocks the effects of opioids (heroin, methadone, opium, codeine, morphine and buprenorphine) if they are used.

**Needle exchange**s are community-based prevention programs that can provide a range of services, including linkage to substance use disorder treatment; access to and disposal of sterile syringes and injection equipment; and vaccination, testing for blood borne viruses, and linkage to care and treatment for infectious diseases.

**Opioid Substitution Treatment (OST)** is the prescribing of a replacement drug such as methadone or buprenorphine. This is one of the most evidence-based treatments on offer to reduce illicit opiate use, overdoses and transmission of blood borne viruses such as HIV and viral hepatitis.

**Opioid Agonist Therapy (OAT**) is a treatment for opioid use disorder that uses opioid agonists, such as methadone or buprenorphine, which bind to the same receptors in the brain activated by the drug of misuse, but in a safer and more controlled manner. These medications reduce withdrawal symptoms and cravings.

**Prescripting** to prevent relapse. A number of relapse prevention medications exist. These include Naltrexone, an opioid antagonist that reduces cravings and decreases the pleasure of alcohol consumption. These medications are used in both the early stages of treatment and for long-term recovery.

**The Reduction and Motivation Programme (RAMP)** provides a safe environment for those in active addiction to explore their dependency and its impact on themselves and others and the life changes needed to gain recovery from substances. It is typically run as a 12-week programme with two sessions per week.

**Rapid prescribing** means that once clients have been referred, they can obtain their prescription (e.g. methadone or buprenorphine) within a short space of time e.g. 24 hours, compared to typically waiting several weeks.

**Residential rehabilitation** is typically based on ‘therapeutic community’ treatment for substance users. These abstinence-based programmes are more intensive and expensive interventions.

**Self-help/mutual aid** support service are most commonly associated with 12-step, Alcoholics Anonymous (AA), Narcotics Anonymous (NA), Cocaine Anonymous (CA). However, many other models are available such as Smart Recovery. They may serve as the primary or only source of behaviour change for many, or as aides to formal treatment, or as a form of continuing care and community support following exit from treatment. These groups are highly accessible and free in communities, and serve as important and readily available resources in substance abuse recovery.

**Testing for BBVs** typically involves testing for blood borne viruses that are more prevalent amongst injecting drug users such as hepatitis C and HIV. Testing facilities may also provide vaccinations including for Hep, A, B and C. These services are commonly offered in needle exchanges and by drug treatment services.

**Therapeutic communities (TCs)** refer to a consciously designed social environment or residential treatment setting in which the social and group process is harnessed with therapeutic intent. The TC promotes abstinence from alcohol and illicit drug use, and seeks to decrease antisocial behaviour and to effect a global change in lifestyle, including attitudes and values. The TC employs the community itself as the agent of healing. The TC views drug abuse as a disorder of the whole person, reflecting problems in conduct, attitudes, moods, values, and emotional management. Treatment focuses on drug abstinence, coupled with social and psychological change that requires a multidimensional effort involving intensive mutual self-help typically in a residential setting.

**Trauma therapies are** most commonly associated with Eye Movement Desensitization and Reprocessing (EDMR).

Appendix 2. Data extraction form

| **Bibliographic Information** | |
| --- | --- |
| Authors | FREE TEXT |
| Title | FREE TEXT |
| Year | FREE TEXT |
| Journal | FREE TEXT |
| Type | Journal Article  Dissertation/Thesis  Report  Other |
| Abstract | FREE TEXT |
| Country | United States of America  Australia  Canada  Denmark  United Kingdom  Netherlands  Ireland  France  South Korea |
| Age | Mean age  Age range |
| Complexity of needs (other challenges participants face, other than homelessness and substance misuse) | Discharge from health services  Mental illness  Complex needs/dual diagnosis  Veteran/Ex-services  Drug and alcohol issues  Survivor domestic violence/abuse  Migrant  Ex-prisoner  Indigenous persons  Care leaver |
| Gender *% (actual number)* | Male  Female  Male and female |
| **Typology classification** | Abstinence-based  Harm reduction  Abstinence-based and harm reduction |
| **Intervention classification** | Psychosocial intervention  Treatment through medication  Non-medication intervention |
| **Comparison** | Other intervention  Treatment as usual  Waitlist |
| Comparison intervention | FREE TEXT |
| **Outcomes measures** | FREE TEXT (e.g. substance abuse, recidivism, employment |
| Substance misuse outcome measure | FREE TEXT |
| Total sample size | FREE TEXT |
| Size of treated group | FREE TEXT |
| Size of control group | FREE TEXT |
| Data to calculate effect size | As required – based on ultimate choice of effect size |
| **Campbell’s Critical Appraisal Tool for Primary Studies** | |
| Study design at end of intervention (Potential confounders taken into account) | DiD with matching, PSM  RCT, RDD, ITT, instrumental variable  Other |
| Study design, longest follow up if applicable (Potential confounders taken into account) | DiD with matching, PSM  RCT, RDD, ITT, Instrumental variable  Other |
| Masking or blinding | Unblinded or no mention of blinding  Any blinding or any mention of blinding |
| Power calculation | Any mention of power calculations as basis for sample size  No mention of power calculation |
| Losses to follow up are presented and acceptable, End of intervention | No mention of attrition  Attrition not reported or below accepted level  Overall and differential attrition close to accepted level  Overall and differential attrition within accepted level  N/A (ex-post study) |
| Losses to follow up are presented and acceptable (Longest follow up, if applicable | No mentioned of attrition  Attrition not reported or below accepted level  Overall and differential attrition close to accepted level  Overall and differential attrition within accepted level  N/A (ex-post study) |
| Definition of intervention are clearly defined | Brief description of intervention  Intervention clearly and fully described  Intervention named but not described, or not named |
| Outcome measures are clearly defined and reliable | Brief description of outcome  Outcome measure clearly and fully described  Outcome named but not described, nor not named |
| Baseline balance (N/A for before versus after) | Imbalance on 2 or less measures  Imbalance on no more than 5 measures  No baseline balance test, or imbalance on 5+ measures |
| Overall confidence in primary study | Low  Medium  High |

Appendix 3. Campbell’s Critical Appraisal Tool for Primary Studies (White et al., 2020)

“The tool for primary studies has seven items which relate to 1) study design, 2) blinding, 3) power calculations, 4) attrition, 5) description of the intervention, 6) outcome definition and 7) baseline balance. A fuller description of these items is given in the technical appendix. Each of these seven items is rated as implying high, medium or low confidence in study findings. Overall quality is assessed using the ‘weakest link in the chain’ principle: our confidence in study findings can only be as high as the lowest rating given to any of the critical items (which are numbers 1, 4, 6 and 7).” (White et al., 2020, p.31)

| Item | | Point in time (where applicable) | Rating |
| --- | --- | --- | --- |
| 1a | Study design (Potential confounders taken into account) | End of intervention | High confidence: RCT, RDD, ITT, instrumental variable  Medium confidence: DiD with matching, PSM  Low confidence: other matching |
| 1b | Study design (Potential confounders taken into account) | Longest follow up (if applicable) | Study design may change at post endline follow up, usually loss of RCT as control becomes treated. Same codes as 1a |
| 2 | Masking or blinding (RCTs only) |  | High confidence: any blinding or any mention of blinding  Medium confidence: no blinding  **Low confidence is not used for this item** |
| 3 | Power calculations are reported |  | High confidence: any mention of power calculations as basis for sample size  Medium confidence: no mention of power calculations  **Low confidence is not used for this item** |
| 4a | Losses to follow up are presented and acceptable* | End of intervention | High: attrition within IES bounds  Medium: attrition within IES liberal bounds  Low: attrition not reported or attrition outside IES bounds  N/A for ex post studies |
| 4b | Losses to follow up are presented and acceptable* | Longest follow up (if applicable) | High: attrition within IES bounds  Medium: attrition close to IES bounds  Low: attrition not reported or attrition outside IES bounds  N/A for ex post studies |
| 5 | Intervention if clearly defined |  | High confidence: intervention clearly and fully described  Medium confidence: brief description of intervention  Low confidence: intervention named but not described, or not named |
| 6 | Outcome measures are clearly defined and reliable |  | High confidence: outcome measure clearly and fully described, preferably with reference to validation  Medium confidence: brief description of outcome  Low confidence: outcome named but not described |
| 7 | Baseline balance (N.A. for before versus after) |  | High confidence: RCT or baseline balance report and satisfactory (imbalance on 2 or less measures)  Medium confidence: Imbalance on no more than 5 measures  Low confidence: Baseline balance not reported, or reported and lack of balance on more than 5 measures |
|  |  |  |  |
|  | **Overall confidence in study findings** | **End of intervention** | **Lowest rating across items 1a, 4a, 6 and 7** |
|  | **Overall confidence in study findings** | **Longest follow up (if applicable)** | **Lowest rating across items 1b, 4b, 6 and 7 (N/A if 1b and 4b N/A)** |
|  | * See table 1 <https://homvee.acf.hhs.gov/sites/default/files/2019-06/HomVEE-Attrition-White_Paper-7-2015.pdf> | | |

Appendix 4. Detailed Risk of Bias appraisal for each included study

[Figure 6](#FIG-06)

Appendix 5. Search details from White et al., (2019): PROTOCOL: Studies of the effectiveness of interventions to improve the welfare of those affected by, and at risk of, homelessness in high‐income countries: An evidence and gap map

**Databases**:

1. Academic databases

• Econlit

• The National Bureau of Economic Research (NBER)

• Social Science Research Network (SSRN)

• International Bibliography of Social Sciences (IBSS)

• Applied Social Sciences Index and Abstracts (ASSIA)

• Social Service Abstract

• Embase

• PubMed

• PsycINFO

• MEDLINE

• WHO’s Global Health Library

• CABI’s Global Health

• ERIC

• CINHAL

• SCOPUS

• Web of Science

• EPPI Centre Evaluation Database of Education Research

2. Evidence and Gap Map Database

• 3ie Evidence and Gap Map Repository

• Global Evidence Mapping Initiative

• Evidence‐Based Synthesis Program (Department of Veteran

Affairs)

3. Systematic review databases

• Swedish Agency for Health Technology Assessment and

Assessment of Social Services

• Collaboration for Environmental Evidence

• Cochrane

• Cochrane

• Campbell

• 3ie Systematic Review Database

• Research for Development

• Epistemonikos

4. Trials registries

AEA Social Science RCT Registry https://www.socialscience

registry.org/.

**Grey literature and websites:**

Homeless Hub https://www.homelesshub.ca/

European observatory on homelessness https://www.feantsaresearch.org/en/publications

United State interagency council on homelessness http://www.usich.gov/

EThOS http://ethos.bl.uk/Home.do

WHO ICTRP http://apps.who.int/trialsearch/

Focus on Prevention http://www.preventionfocus.net/

Social Policy and Practice http://www.spandp.net/

10,000 home campaigns https://en.wikipedia.org/wiki/100,000_Homes_Campaign

Anti‐poverty committee https://en.wikipedia.org/wiki/Anti‐Poverty_Committee

Back on my feet https://en.wikipedia.org/wiki/Back_on_My_Feet_(non‐profit_organization)

Feantsa https://www.feantsa.org/

National Coalition Homeless https://nationalhomeless.org/

Homelessness Australia https://www.homelessnessaustralia.org.au/

Mission Australia https://www.missionaustralia.com.au/publications/position‐statements/homelessness

National Alliance to end homelessness https://endhomelessness.org/

Institute of global homelessness https://www.ighomelessness.org/

Homelessness link https://www.homeless.org.uk/

Crisis https://www.crisis.org.uk/about‐us/how‐we‐work/

Housing first https://housingfirsteurope.eu/about‐the‐hub/

Canadian Alliance to end homelessness https://housingfirsteurope.eu/about‐the‐hub/

Social work and policy institutes http://www.socialworkpolicy.org/research/homelessness.html

Association of housing advice services https://www.ahas.org.uk/

Centre point <https://centrepoint.org.uk/>

Homelessness trust funds https://housingtrustfundproject.org/htf‐elements/homeless‐trust‐funds/

Meliville charitable trust https://melvilletrust.org/category/resourcesreports/

Conrad H Hilton foundation https://www.hiltonfoundation.org/priorities/homelessness#resources

Abt Associates https://www.abtassociates.com/

Mathematica https://www.mathematica‐mpr.com/

American Institutes of Research https://www.air.org/

Rand https://www.rand.org/

MDRC https://www.mdrc.org/

**Additional searches using Google and Google Scholar.**

**SAMPLE SEARCH STRING**

Search string/keywords (for ovid medline platform)

**Study design key words**

– (“quasi experiment*” or quasi‐experiment* or “random* control*

trial*” or “random* trial*” or RCT or (random* adj3 allocat*) or

matching or “propensity score” or PSM or “regression discontinuity”

or “discontinuous design” or RDD or “difference in difference*” or

difference‐in‐difference* or “diff in diff” or “case control” or cohort or

“propensity weighted” or propensity‐weighted or “interrupted time

series” or (before adj5 after) or (pre adj5 post) or ((pretest or pre

test) and (posttest or post test)) or “research synthesis” or “scoping

review” or “rapid evidence assessment” or “systematic literature

review” or “Systematic review” or “Meta‐analy*” or Metaanaly* or

“meta analy*” or “Control* evaluation” or “Control treatment” or

“instrumental variable*” or heckman or IV or (quantitative or

“comparison group*” or counterfactual or “counter factual” or

counter‐factual or experiment*) adj3 (design or study or analysis))

or QED or evaluation).ti,ab,kw

– OR

– clinical trial/or clinical trial, phase i/or clinical trial, phase ii/or clinical

trial, phase iii/or clinical trial, phase iv/or controlled clinical trial/or

randomised controlled trial/or pragmatic clinical trial/

– controlled clinical trials as topic/or non‐randomised controlled

trials as topic/or randomised controlled trials as topic/or

pragmatic clinical trials as topic/or case‐control studies/or

retrospective studies/or controlled before‐after studies/or interrupted

time series analysis/or random allocation/or cohort

studies/or follow‐up studies/or longitudinal studies/or prospective

studies/or retrospective studies/or propensity score/or regression

analysis/or evaluation studies/or matched‐pair analysis

– (“quasi experiment*” or quasi‐experiment* or “random* control* trial*”

or “random* trial*” or RCT or (random* adj3 allocat*) or matching or

“propensity score” or PSM or “regression discontinuity” or “discontinuous

design” or RDD or “difference in difference*” or difference‐indifference*

or “diff in diff” or “case control” or cohort or “propensity

weighted” or propensity‐weighted or “interrupted time series” or

(before adj5 after) or (pre adj5 post) or ((pretest or pre test) and

(posttest or post test)) or “research synthesis” or “scoping review” or

“rapid evidence assessment” or “systematic literature review” or

“Systematic review” or “Meta‐analy*” or Metaanaly* or “meta analy*”

or “Control* evaluation” or “Control treatment” or “instrumental

variable*” or heckman or IV or ((quantitative or “comparison group*”

or counterfactual or “counter factual” or counter‐factual or experiment*)

adj3 (design or study or analysis)) or QED).ti,ab,kw.

(“meta regression” or “meta synth*” or “meta‐synth*” or “meta analy*”

or “metaanaly*” or “meta‐analy*” or “metanaly*” or “metaregression” or

“metaregression” or “methodologic* overview” or “pool* analys*” or “pool*

data” or “quantitative* overview” or “research integration”).ti,ab,sh.

OR

(review adj3 (effectiveness or effects or systemat* or synth* or

integrat* or map* or methodologic* or quantitative or evidence or

literature)).ti,ab,sh.

**Homelessness keywords**

– homeless persons/or homeless youth

– (evict* or homeless* or "housing excl*" or “residential stability”

or ((street* or private or improvised or shelter* or emergency or

temporary or insecure or overcrowded or precarious or stable or

marginal*) adj3 (dwell* or house* or housing or accommodation))

or (street adj3 (life or living or lives or youth* or child* or people or

person*)) or runaway* or “Run away from home” or “Running away”

or “Ran away” or “Going missing” or “Bag lady” or Houseless* or

Unhoused or “without a roof” or Roofless or (rough adj3 sleep*) or

Destitut* or “Skid row*” or “sleepers out”).ti,ab,kw.

– (“Housing first” or “Pathways to Housing” or “Homeless Veterans

Reintegration Program” or “Access to Community Care and Effective

Services and Supports” or 'Support* Housing Program” or “Housing

and Urban Development–Veterans Affairs Supported Housing program”

or “HUD‐VASH” or “Sober Transitional Housing and Employment

Project” or “sober house placement*” or “Housing ladders” or

“Staircase housing” or “low threshold housing” or “Critical Time

Intervention”).ti,ab,kw
